# Metabolism-Guided Optimization of Tryptophanol-Derived Isoindolinone p53 Activators

**DOI:** 10.3390/ph16020146

**Published:** 2023-01-19

**Authors:** Valentina Barcherini, Joana B. Loureiro, Ana Sena, Catarina Madeira, Paula Leandro, Lucília Saraiva, Alexandra M. M. Antunes, Maria M. M. Santos

**Affiliations:** 1 Research Institute for Medicines (iMed.ULisboa), Faculty of Pharmacy, Universidade de Lisboa, Av. Prof. Gama Pinto, 1649-003 Lisboa, Portugal; 2 LAQV/REQUIMTE, Laboratório de Microbiologia, Departamento de Ciências Biológicas, Faculdade de Farmácia, Universidade do Porto, 4050-313 Porto, Portugal; 3 Centro de Química Estrutural (CQE), Institute of Molecular Sciences, Departamento de Engenharia Química, Instituto Superior Técnico (IST), Universidade de Lisboa, 1049-001 Lisboa, Portugal

**Keywords:** cancer, p53, metabolic stability, tryptophanol-derived isoindolinones, hit-to-lead optimization

## Abstract

For the first time, the pharmacokinetic (PK) profile of tryptophanol-derived isoindolinones, previously reported as p53 activators, was investigated. From the metabolites’ identification, performed by liquid chromatography coupled to high resolution tandem mass spectrometry (LC-HRMS/MS), followed by their preparation and structural elucidation, it was possible to identify that the indole C2 and C3 are the main target of the cytochrome P450 (CYP)-promoted oxidative metabolism in the tryptophanol-derived isoindolinone scaffold. Based on these findings, to search for novel p53 activators a series of 16 enantiopure tryptophanol-derived isoindolinones substituted with a bromine in indole C2 was prepared, in yields of 62–89%, and their antiproliferative activity evaluated in human colon adenocarcinoma HCT116 cell lines with and without p53. Structural optimization led to the identification of two (*S*)-tryptophanol-derived isoindolinones 3.9-fold and 1.9-fold more active than hit SLMP53-1, respectively. Compounds’ metabolic stability evaluation revealed that this substitution led to a metabolic switch, with the impact of Phase I oxidative metabolism being minimized. Through differential scanning fluorimetry (DSF) experiments, the most active compound of the series in cell assays led to an increase in the protein melting temperature (*T*_m_) of 10.39 °C, suggesting an effective binding to wild-type p53 core domain.

## 1. Introduction

**Figure 1:**
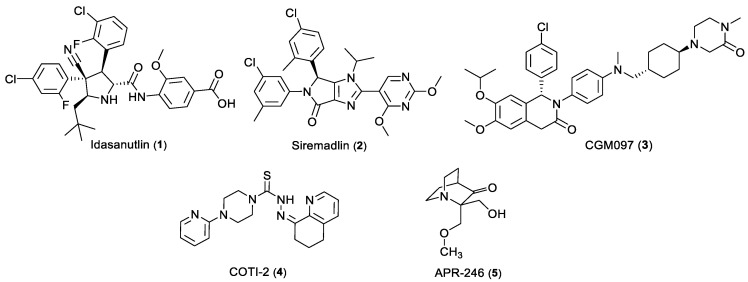
Chemical structure of p53-activating small molecules in clinical development for CRC.

Colorectal cancer (CRC) figures currently as the third most common cancer and it ranks second as cause of cancer-related death globally [1]. Despite the latest progresses made in understanding CRC pathophysiology, poor therapeutic options are still available. To date, systemic treatment is based on combination therapies of DNA-damaging agents 5-fluorouracil (5-FU), oxaliplatin, and irinotecan with targeted drugs such as monoclonal anti-epidermal growth factor receptor (EGFR) antibodies and anti-angiogenic agents targeting the vascular endothelial growth factor (VEGF) pathway [2,3]. These combined treatment options have enhanced the median overall survival from about one year with 5-FU alone to up to three years with current standard therapies [4]. Nevertheless, patients develop resistance to available treatments [5] and chemotherapy-derived side-effects [6]. Therefore, the identification of novel therapeutics, with optimized pharmacological impact and minimized toxicity is urgently needed. Targeted therapies have gained importance in the field, and the tumor protein p53 is considered a relevant therapeutic target in CRC [7,8], continuing to figure as the most inactivated protein in spontaneous human cancers [9,10]. Inactivation of p53 can occur by mutation of the *TP53* gene or by upregulation of its negative regulators MDM2 and/or MDM4 [11,12,13,14]. As guardian of the genome, p53 controls programmed cell death, tightly regulating apoptosis [15,16]. Protein p53 is the center of multiple essential cellular processes and under specific stress signals such DNA damage, oncogene activation and hypoxia, promotes cell cycle arrest coordinating DNA repair, senescence and autophagy [17,18,19,20]. Pharmacological restoration of tumor suppressor p53 function is a promising approach in cancer therapy. Although few small molecules have followed clinical development, p53-based therapy is still not clinically available for cancer treatment. Among the p53-activating small molecules in clinical trials for CRC (Figure 1), Idasanutlin (**1**, NCT03555149), Siremadlin (**2**, NCT03714958), and CGM097 (**3**, NCT01760525) effectiveness is limited only to wild-type (wt) p53 expressing CRC, and small molecule COTI-2 (**4**, NCT02433626) is a mutant p53 activator which mechanism of action results still unclear [21,22]. Another relevant clinical candidate, APR-246 (**5**), retains a p53-independent inhibitory activity in CRC [23]. Consequently, additional research is needed to discover novel p53-binding compounds to defeat CRC.

**Figure 2:**
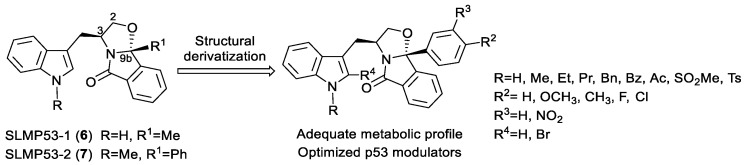
Structural optimization of tryptophanol-derived isoindolinones as p53 activators.

In previous work, enantiopure tryptophanol-derived isoindolinone SLMP53-1 (**6**) was identified as a novel direct reactivator of wt p53 and DNA-contact mutant p53 R280K, having p53-dependent in vivo antitumor activity [24]. Optimization of SLMP53-1 (**6**) led to the identification of SLMP53-2 (**7**), a small molecule that restores wt-like conformation and DNA-binding ability of mutp53-Y220C, by enhancing its interaction with the heat shock protein 70 (Hsp70), in hepatocellular carcinoma [25]. This compound also showed promising therapeutic potential in advanced melanoma and in vitro activity in HCT116 cells [26,27]. Due to the high failure rates in drug development, mainly related to poor prediction of drug metabolism, development of new bioactive molecules with adequate metabolic properties is a basic requirement for preclinical success and development [28,29]. For this reason, a preliminary study of the pharmacokinetic profile of SLMP53-1 (**6**) and SLMP53-2 (**7**) was conducted before proceeding with the optimization of tryptophanol-derived isoindolinones as p53 activators. Determination of Phase I and Phase II metabolites by LC-ESI-HRMS/MS and identification of the possible reactive metabolites allowed us to identify the metabolic liabilities of the tryptophanol-derived isoindolinone scaffold. Biomimetic synthesis [30] of Phase I metabolites, identified in human liver microsomes (HLM) incubations, was conducted and their anti-proliferative activity in HCT116 cells evaluated. Subsequent scaffold structural derivatization was carried out by blocking the 2-position of the indole with bromine (Figure 2). The antiproliferative activity of the novel compounds and contribution to the p53 pathway was evaluated in human colorectal carcinoma HCT116 cell lines. To evaluate the impact on the metabolic stability of the performed structural optimizations, the pharmacokinetic (PK) profile of the most active compounds was also assessed. To assess the putative binding to p53, in vitro thermostability assays with the most promising compounds towards the wt p53 DNA-binding domain (p53DBD) were performed, thus allowing us to extend our knowledge on the mode of action through which the scaffold promotes protein p53 reactivation.

## 2. Results and Discussion

### 2.1. Metabolic Stability and Metabolite Profiling of (S)-Tryptophanol Derivatives SLMP53-1 (***6***), and SLMP53-2 (***7***)

SLMP53-1 (**6**) and SLMP53-2 (**7**) were stable towards plasmatic enzymes for 3 h (Figure S1, SI). The stability of these compounds was also evaluated in HLM incubations (Figures S2 and S3, SI) [31]. The obtained intrinsic clearance (3.67 and 2.75 mL min^−1^ Kg^−1^) and half-lives (138 and 147 min) values suggest that SLMP53-1 (**6**) and SLMP53-2 (**7**) are low clearance compounds [32].

**Figure 3:**
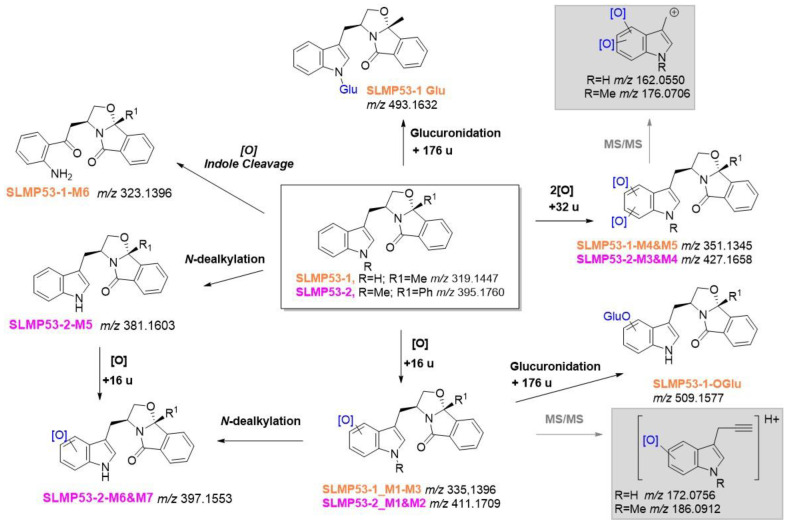
LC-HRMS/MS-based metabolite profile of (*S*)-tryptophanol-derived isoindolinones SLMP53-1 (**6**) (in orange) and SLMP53-2 (**7**) (in pink). In grey boxes are highlighted the diagnostic ions that attested for the metabolic transformation at the indole ring.

Very similar metabolite profiles were identified for these two hit compounds by LC-ESI(+)-HRMS/MS. Similarly to what was observed in our previous PK studies with tryptophanol-derived oxazolopyrrolidone lactams [33], multiple isomeric metabolites stemming from the metabolic incorporation of one or two oxygens were identified (Figure 3) (SI, Figure S4). Whereas the fragmentation pattern displayed by these ions do not allow us to assign the exact location of the metabolic transformation(s), the consistent presence of diagnostic fragment ions (Figure 3 and SI, Figures S5–S13), attests the selective incorporation of 1 or 2 oxygens (mass increase of 16 and 32 u, respectively) at the indole moiety. Additionally, the metabolic product of indole ring cleavage, following initial oxidation of the parent drug, was selectively identified in SLMP53-1 (**6**) incubations (Figure 3 and SI, Figure S7). This suggests that the presence of the methyl group, at the indole nitrogen of SLMP53-2 (**7**), hampers this metabolic transformation. Nonetheless, SLMP53-2 (**7**) is prone to *N*-dealkylation pathways. In fact, three *N*-indole dealkylation products were identified for this hit compound (Figure 3): one product from its direct *N*-dealkylation; and three products, presumably formed upon *N*-dealkylation of metabolites, stemming from one-oxygen incorporation (SI, Figures S12 and S13). Although observed, *N*-dealkylation is a minor metabolic transformation as shown by the plot of the relative intensities of each metabolite over reaction time. In fact, metabolites stemming from the incorporation of one oxygen are consistently the main Phase I metabolic transformation detected for SLMP53-1 (**6**) and SLMP53-2 (**7**) (SI, Figures S14 and S15). Noteworthy is the fact that the same metabolite profile was obtained for SLMP53-1 (**6**) incubations run in S9 rat liver fractions. This result suggests that specie-dependent dimorphic metabolite profiles are not expected for this scaffold, which is relevant since this hit was tested in vivo in a rodent model. Additionally, this result suggests that this scaffold is not prone to degradation by cytosolic metabolic enzymes.

Glucuronidation is a key contributor for decreased drug bioavailability, by excretion [34,35]. To evaluate the ability of (*S*)-tryptophanol-derived isoindolinones to undergo this Phase II pathway, additional incubations were performed in alamethicin induced-HLM in the presence of nicotinamide adenine dinucleotide phosphate (NADPH) and uridine diphosphate glucuronic acid (UDPGA) co-factors [34]. While no Phase II metabolites were identified in SLMP53-2 (**7**) incubations, two glucuronic acid conjugates were identified for SLMP53-1 (**6**), by LC-ESI(-)-HRMS/MS: a Phase II metabolite compatible with direct conjugation of the SLMP53-1 (**6**) *N*-indole with glucuronic acid (SI, Figure S16); and one product formed upon glucuronidation of one of the Phase I metabolites, stemming from one-oxygen incorporation. The presence of the glucuronic acid moiety was attested by the observation of diagnostic neutral losses and fragment ions, [36] in the tandem mass spectra of these Phase II metabolites (SI, Figure S17). The fact that SLMP53-2 (**7**) is not prone to these Phase II pathways, suggests that the presence of the methyl group at the *N*-indole might be beneficial for increased bioavailability.

### 2.2. Synthesis and Antiproliferative Activity of SLMP53-1 Phase I Metabolites

Since SLMP53-1 (**6**) and SLMP53-2 (**7**) showed very similar metabolite profiles, we decided to use SLMP53-1 (**6**) as a model for the subsequent studies involving the evaluation of the activity of the main Phase I metabolites, following their synthesis in a preparative scale. This also constituted an opportunity to perform a full structural characterization of the Phase I metabolites, whose preliminary LC-HRMS-MS characterization only attested the incorporation of one or two oxygens at the indole moiety but did not allow the identification of the exact location of these transformations.

**Scheme 1:**
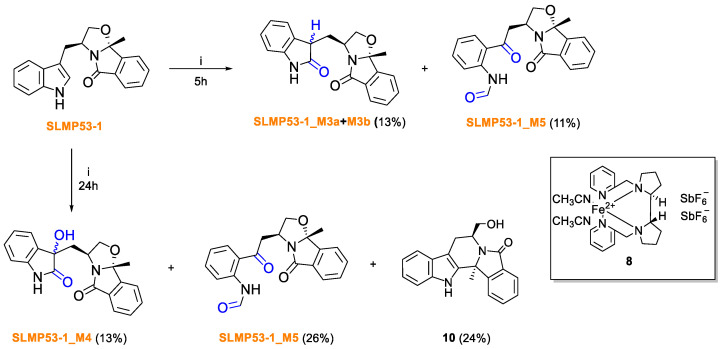
Direct biomimetic oxidation of SLMP53-1 (**6**). i. White–Chen catalyst (**8**) 10 mol%, H_2_O_2_ 0.45 eq. (35% *v*/*v*), AcOH 5.00 eq., ACN at 40 °C.

The synthesis of main Phase I metabolites of the p53 activator SLMP53-1 (**6**) was achieved by biomimetic oxidation catalyzed by the nonheme iron-based complex (2*S*, 2′*S*-(-)-[*N*,*N*′-Bis(2-pyridylmethyl)]-2,2′-bipyrrolidinebis(acetonitrile)iron(II) hexafluoroantimonate, [Fe(II)(*S*,*S-PDP*)(NCCH_3_)_2_] (SbF_6_)_2_, (**8**) (also known as White–Chen catalyst). Reactions were performed in acetonitrile solutions of SLMP53-1 (**6**) at 40 °C, in the presence of hydrogen peroxide, acetic acid and 10 mol% of catalyst (Scheme 1) [30].

LC-ESI(+)-HRMS/MS analysis showed that the product profile obtained from biomimetic oxidation of SLMP53-1 (**6**) is similar to its respective metabolite profile, obtained from HLM incubations. Coherently, all products share compatible retention times and similar tandem HRMS profiles (Figures S18–S25). Whereas very similar results were obtained using *N*,*N′*-Dimethyl-*N*,*N′*-bis(2-pyridylmethyl)ethane-1,2-diamine Iron(II) bis(triflate), [Fe(II)(bpmen)OTf_2_] (**9**) (results not shown), the commercial availability of White-Chen catalyst (**8**) justifies our selection for the scale-up synthesis of SLMP53-1 (**6**) metabolites. While metabolites SLMP53-1_M1, M2 and M6 were identified as reaction products by HRMS analysis (SI, Figures S20–S22) only vestigial amounts of these compounds were formed, which hampered their subsequent isolation. Of note is the fact that **10**, the product of intramolecular aminoalkylation [37], was the main reaction product due to the acidic conditions needed for the catalyst system used. However, for the 5 h reaction the purification method was only focused on the isolation of SLMP53-1_M3a, M3b and M5. Following isolation of SLMP53-1_M3a, M3b, M4 and M5, NMR analysis unequivocally evidenced the metabolic oxidations at positions 2 and 3 of the indole core (SI, Figures S26 and S32). The main Phase I metabolites identified in HLM incubation correspond to the two diastereomeric oxindoles, SLMP53-1_M3 (Scheme 1). The two metabolites stemming from the incorporation of two oxygens (compatible with the 32 u mass increment) correspond to products of a subsequent metabolic hydroxylation at the C-3 indole position to SLMP53-1_M4, followed by the oxidative cleavage of the pyrrole ring which yield a formamide metabolite, SLMP53-1_M5, similar to *L*-formyl kynurenine, a catabolic product of *L*-tryptophan [38].

The antiproliferative activity of SLMP53-1 Phase I metabolites was subsequently evaluated in human colon adenocarcinoma HCT116 cell line expressing wt p53 (HCT116 p53^+/+^). To evaluate the selectivity for the p53 pathway, the metabolites were also screened in HCT116 cells in which p53 has been knocked out (HCT116 p53^−/−^). Growth inhibition values (GI_50_) are listed in Table 1. All tested SLMP53-1 metabolites showed loss of potency and loss of selectivity towards the p53 pathway compared to SLMP53-1 (**6**). SLMP53-1_M4 was not tested due to its instability.

**Figure 4:**
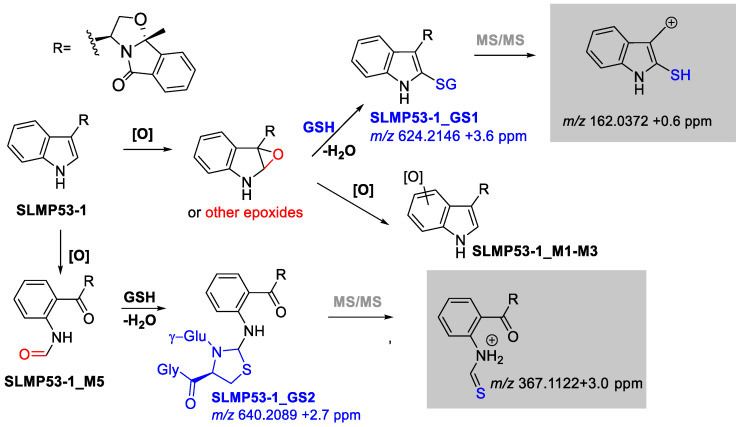
Possible SLMP53-1 (**6**) bioactivation pathways. In grey boxes are highlighted the diagnostic fragment ions that attested for the GSH addition at the indole ring.

With impact on decreased attrition rates [39,40], evaluation of bioactivation risk is considered a key issue during the drug development process. To investigate potential bioactivation pathways, SLMP53-1 (**6**) was incubated with S9 rat liver fraction and HLM in the presence of glutathione (GSH). Two GSH adducts were identified under these conditions (Figure 4): (1) SLMP53-1_GS1, plausibly stemming from the ring-opening of an arene oxide formed in the indole core, which are the expected CYP-mediated metabolic intermediates of the Phase I metabolites SLMP53-1_M1-M3; and (2) SLMP53-1_GS2, compatible with a cyclic adduct which might be formed from the initial thiol nucleophilic addition to the *N*-formyl amide carbonyl group of SLMP53-1_M5, followed by intramolecular addition of the α-nitrogen of the cysteine residue (SI, Figures S33 and S34) [41]. Interestingly, no reaction products were obtained upon *L*-lysine incubation with SLMP53-1_M5, followed by reductive stabilization. Whereas the GSH-derived adducts suggest potential bioactivation pathways, the SLMP53-1 in vivo assays were not associated with toxicity alerts [24]. Therefore, the formation of these adducts is likely to be more associated to excretion rather than toxicity pathways.

### 2.3. Synthesis of Bromine-Enriched Tryptophanol-Derived Isoindolinones

**Scheme 2:**
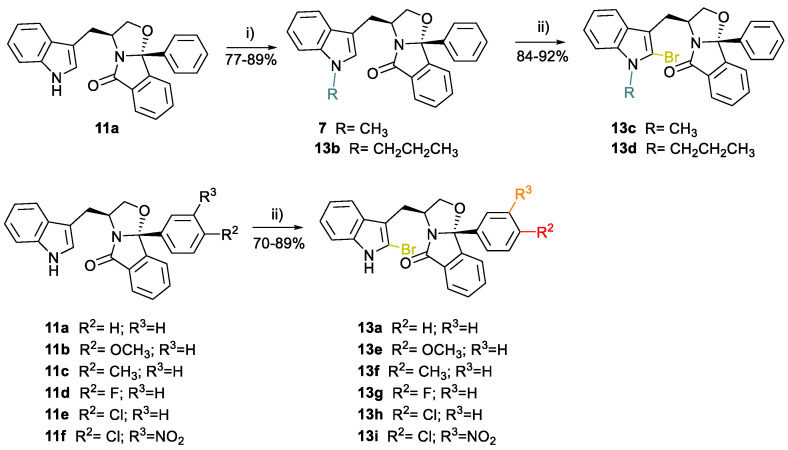
Synthesis of 2-bromo (*S*)-tryptophanol-derived isoindolinones **13a-i**. Reagents and conditions: (i) 1. NaH, 0 °C DMF; 2. MeI, rt (**7**) or 2. PrBr, rt (**13b**); (ii) PyHBr_3_, THF/DCM, 0 °C, instantaneous.

Based on the results obtained from the PK study on compounds SLMP53-1 (**6**) and SLMP53-2 (**7**), we searched for synthetic strategies aiming to optimize the robustness of the tryptophanol-derived isoindolinone scaffold towards CYPs oxidative metabolism. Bromine insertion at the indole C2 may electronically and metabolically promote the indole pyrrole ring stabilization due to its effect as deactivating group and to its steric hindrance [42]. Moreover, indole nitrogen protection was suggested to enhance the metabolic stability of this class of compounds. Therefore, a series of bromine tryptophanol-derived isoindolinones with phenyl or *para*- and *meta*-substituted phenyl groups in position C9b was prepared. Compounds **13c** and **13d** were obtained with yields of 92% and 84%, respectively, by reaction of tryptophanol-derived isoindolinone **11a** with the corresponding alkyl halide in the presence of sodium hydride [26], followed by pyridinium bromide perbromide (PyHBr_3_)-promoted indole bromination (Scheme 2). Compounds **13a**, **13e** to **13i** were prepared by PyHBr_3_-promoted bromination of the corresponding (*S*)-tryptophanol-derived isoindolinones **11a** and **11b**-**f** [26], with yields ranging from 70% to 90% (Scheme 2).

**Scheme 3:**
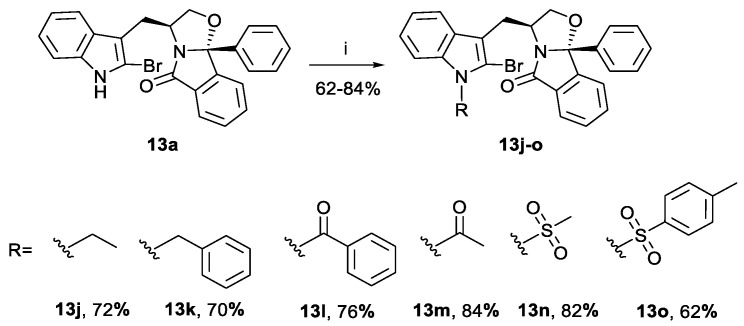
Preparation of bromine-enriched isoindolinones **13j-o**. Reagents and conditions: (i) 1. NaH, 0 °C DMF; 2. RI, rt (**13j**) or RCl, rt (**13k**, **13l**, **13n** and **13o**) or R_2_O, rt (**13m**).

Compounds **13j-o** were prepared by indole nitrogen derivatization of bromine-enriched (*S*)-tryptophanol isoindolinone **13a**, with yields of 62–84% (Scheme 3). The starting material was not totally consumed in the *N*-tosylation reaction, probably due to steric hindrance of the *para*-toluenesulfonate moiety leading to a lower yield of compound **13o**.

Compounds **13a** and **13c-o** were analyzed by ^1^H and ^13^C nuclear magnetic resonance (NMR) experiments (SI, Figures S35–S38). Formation of the C-Br bound was confirmed by the disappearance of the H-2 indole singlet present in the ^1^H NMR of the starting isoindolinones, the C-2 signal between 108.7–109.4 ppm for compounds **13a**, **13e-i**, **13m** and **13n,** and between 113.0–114.1 ppm for compounds **13c**-**d** and **13j**-**k**. The presence of benzoyl and tosyl groups induces a major shift for this signal appearing at chemical shifts 119.3 ppm (compound **13l**) and at 121.6 ppm (compound **13o**), respectively. The CH_2_-indole diastereotopic protons appear as two doublets of doublets at 2.69–2.97 ppm and 3.02–3.28 ppm. The stereogenic center C-3 signal appears in at 55.6–55.7 ppm, except for compounds **13i** and **13o** where this signal appears at chemical shift 57.1 and 54.9 ppm, respectively. The C-9b stereogenic center signal appears for all compounds between 100.0 and 101.2 ppm. These values are in agreement with NMR signals observed for the parent compounds **6** and **7**, confirming the 3,9b-(*S*,*R*)-relationship for all compounds [26].

### 2.4. Structure–Activity Relationship (SAR) Studies

**Figure 5:**
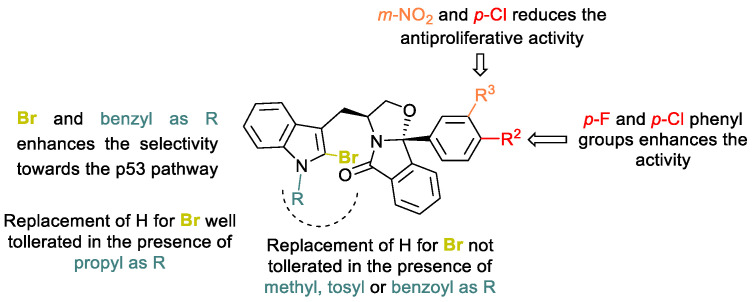
SAR trends for halogen-enriched (*S*)-tryptophanol-derived isoindolinones **13a** and **13c**-**o**.

The antiproliferative activity of bromine-enriched tryptophanol-derived isoindolinones **13a** and **13c-o** was evaluated in human colon adenocarcinoma HCT116 cells (Table 2 and Figure 5). Four compounds were more active than hit SLMP53-2 (**7**).

The insertion of a bromine in the unprotected indole derivatives **13a** and **13i** led to loss of the activity and selectivity toward the p53 pathway (compounds **13c**-**d** and **13j**-**o)**. A similar effect was observed when the bromo derivatives have an ethyl, an acetyl or a mesyl group in the indole nitrogen (compounds **13j**, **13m** and **13n**). Whereas a loss of selectivity is observed for compounds **13j** and **13n**, introduction of an acetyl group (**13m**) promotes 1.7-fold of selectivity in p53-expressing HCT116 cell line. Introduction of a benzoyl group led to a derivative 2.8-fold less active than hit compound SLMP53-2 (**8**) but the derivative retains selectivity for the p53 pathway. Insertion of a bromine atom at the C2-indole moiety of hit compound SLMP53-2 (**7**) resulted in a compound 3-fold less active and less selective for the cell line with p53. The presence of a tosyl, a bulkier electron-withdrawing group, led to a 4.0-fold less active derivative compared with a hydrogen (SLMP53-2 (**7**) vs. **13o**). Introduction of a benzyl moiety at the indole nitrogen and introduction of the bromine at the C-2 indole led to compound **13k** with comparable antiproliferative activity to hit compound SLMP53-2 (**7**). Interestingly, compound **13k** showed 3.8-fold selectivity for the p53 pathway, being 1.8-fold more selective for the p53 pathway than SLMP53-2 (**7**) (3.8 for **13k** versus 2.1 for SLMP53-2 (**7**)). Moreover, although bromine insertion at the C-2 indole led to 1.2- fold and 1.5-fold increase in the antiproliferative activity as in C9b *para*-Fluoro and C9b *para*-Chloro isoindolinones **13g** and **13h**, respectively, such optimization led to loss of selectivity for the p53 pathway. Additionally, the presence of a *para*-chloro and *meta*-nitro substituted phenyl moiety, together with insertion of the bromine at the C2-indole, was not beneficial for the antiproliferative activity and compound selectivity for the p53 pathway. The most promising derivative of this series was C-2-bromine-enriched (*S*)-tryptophanol-derived isoindolinone **13d** with a GI_50_ value of 4.0 µM in HCT116 p53^+/+^, being 2-fold more active than SLMP53-2 (**7**). In addition, compound **13d** showed 1.9-fold selectivity for p53-expressing HCT116 cell line (**13d**, GI_50_ p53^−/−^ = 7.5 µM). To evaluate the impact of stereochemistry on the antiproliferative activity, the enantiomer of **13d**, compound **13d′**, was synthesized and tested against HCT116 cells with and without p53. However, a 3,9b-(*R*, *S*) stereochemistry induced an important loss of antiproliferative activity and selectivity for the p53 pathway (**13d′**, HCT116 p53^+/+^ GI_50_ = 31.1 ± 4.0 µM; HCT116 p53^−/−^ GI_50_ = 25.4 ± 1.2 µM).

### 2.5. Determination of the Metabolic Stability and Metabolite Profiling of 13d and 13k

While **13d** and **13k** can be classified as low clearance compounds [32], **13d** was shown to be slightly less stable towards HLM enzymes than SLMP53-2 (**8**) (Table 3) [32]. Nonetheless, **13k** displayed not only an improvement in the antiproliferative activity but also equivalent metabolic stability, when compared with SLMP53-2 (**7**) (Table 3).

**Figure 6:**
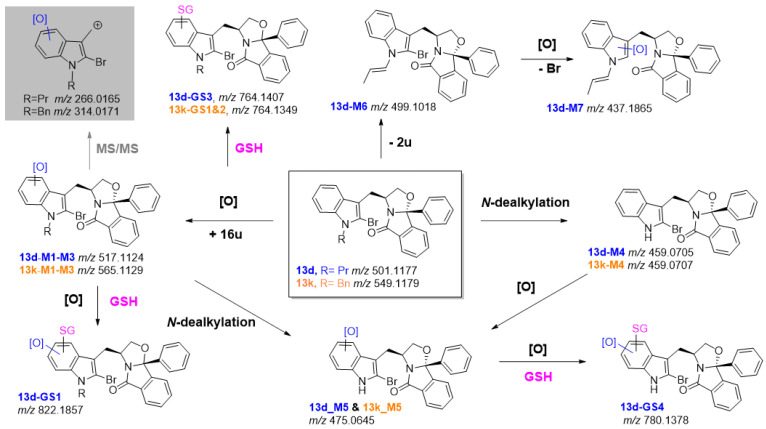
LC-HRMS-MS-based metabolite profile of compounds **13d** (in blue) and **13k** (in orange) and potential bioactivation pathways. In the grey box is the diagnostic fragment ion that attested for the metabolic transformation at the indole ring.

Notably, the C9b-phenyl moieties of **13d** and **13k** were not prone to metabolic degradation. The *N*-dealkylation and indole mono-oxidation were the sole metabolic pathways identified for **13k** (Figure 6, SI, Figures S44–S46). However, for **13d**, in addition to these metabolic pathways, metabolites stemming from reduction in the *N*-propyl substituent followed by hydroxylation with bromine loss were also identified (Figure 6, SI, Figures S47 and S48). Remarkably, metabolites stemming from the pyrrole ring opening were not detected for these compounds. This suggests that the co-existence of a bromine at C-2 and propyl or benzyl substituents at the indole nitrogen protects the indole ring from this catabolic pathway. This might be a relevant issue, since no antiproliferative activity was observed for SLMP53-1 indole-degradation products. However, a metabolic shift towards the formation of potential phenolic metabolites was observed. In fact, the plots of the relative intensities of each metabolite over time (SI, Figures S49 and S50) suggest that these metabolites (**M1**, **M2** and **M3**) are consistently the main metabolic transformation detected for both compounds **13d** and **13k**.

The presence of a bromine substituent at the C-2 position of **13d** and **13k**, led us to investigate if it could constitute a metabolic liability towards the reaction with bionucleophiles. However, this hypothesis was discarded since no products compatible with the addition of GSH, and the concomitant loss of bromine, were detected following incubation of this bionucleophile with **13d** and **13k,** either with or without enzymatic conditions (in S9 rat liver incubations). Nonetheless, GSH adducts compatible with the ring-opening of arene oxides intermediates were identified in the S9 rat liver fraction incubation run in the presence of GSH (SI, Figures S51–S53). It should be noted, however, that **13k** seems to be less prone to this type of bioactivation pathways. In fact, contrasting with **13d,** for this derivative no adducts stemming from the bioactivation of Phase I metabolites were identified. Therefore, despite displaying similar HLM intrinsic clearance to hit SLMP53-2, **13k** shows less ability towards the catabolic pathways involving the indole ring opening (which are associated with activity loss) and more limited ability to yield reactive metabolites, when compared with its analogue **13d**.

### 2.6. Determination of the Thermostability of Wild-Type p53 DNA-Binding Domain by DSF Experiments

We next decided to evaluate whether **13d** binds to protein p53 (Table 4). To this end the wt-p53 core domain (DNA Binding Domain; p53DBD; residues 94–312) [43] were expressed and purified, and using differential scanning fluorimetry (DSF) the protein melting temperature (*T*_m_), in the absence and presence of tested compounds, was determined as an approach to confirm protein binding [44]. Methylene quinuclidinone (MQ) (2 mM) [45] was used as positive control. The assays were performed in the presence of dithiothreitol (DTT) or tris(2-carboxyethyl)phosphine (TCEP). In presence of DTT compound **13d** did not increase the wt p53DBD *T*_m_ (Table 4). However, in presence of the reducing agent TCEP compound **13d** promoted protein thermostability, increasing the *T*_m_ of the wt p53DBD from 40.99 ± 0.07 °C to 51.38 ± 0.38 °C (Δ*T*_m_ = 10.39 °C) (Table 4; SI Figure S54). The *T*_m_ increase obtained in the presence of 2 mM MQ and 1 mM TCEP (Δ*T*_m_ = 2.11 °C) or DTT (Δ*T*_m_ = 2.28 °C) are in agreement with published data, thus validating the assay [45]. This first screening highlights that compound **13d** interacts directly with wt-p53DBD (SI, Figure S54) and that stabilization of the p53 core domain is a putative mechanism for the observed effect in cellular assays.

## 3. Materials and Methods

### 3.1. General Information

All reagents were purchased from commercial suppliers and used without further purification unless otherwise indicated. (*S*)-Tryptophanol-derived isoindolinones SLMP53-1 (**6**), SLMP53-2 (**7**) and compounds **11a**-**f** and **13b** were synthesized as previously reported [24,25,26,46]. (*S*)- and (*R*)-tryptophanol were synthesized by reduction in *L*- and *D*-tryptophan with Lithium Aluminium hydride [47]. Except in the biomimetic reactions, all reaction solvents were distilled from appropriate drying agents prior to use. All reactions were performed under nitrogen atmosphere.

GIBCO^TM^ Human liver microsomes (HLM), GIBCO^TM^ rat S9 liver homogenate and Vivid^®^ regeneration system 100X were obtained from Thermo-Fisher Scientific ((Waltham, USA). (2*S*,2′*S*-(-)-[*N*, *N*′-Bis(2-pyridylmethyl)]-2,2′-bipyrrolidinebis(acetonitrile)iron(II) hexafluoroantimonate, [Fe(II)(*S*,*S-PDP*)(NCCH_3_)_2_] (SbF_6_)_2_, (**8**) catalyst and acetic acid and hydrogen peroxide 35% wt were purchased by Sigma-Aldrich Quimica S.A. (Madrid, Spain). Colon adenocarcinoma HCT116 cell lines expressing wt p53 (HCT116 p53^+/+^) and its p53-null isogenic derivative (HCT116 p53^−/−^) were provided by B. Vogelstein (The Johns Hopkins Kimmel Cancer Center, Baltimore, MD, USA).

The pET15b expression construct to produce the human wt p53 core domain was acquired to Addgene [48] and sypro orange to Invitrogen (5000× stock solution).

### 3.2. In Vitro Stability Assays

#### 3.2.1. Human Plasma Incubations

Human plasma incubations were performed in duplicate, at 37 °C, by standard methodology [49]. Specifically, incubations contained 40 μM of the test compound for a final volume of 1 mL. The mixtures were incubated for a total time of 24 h, and aliquots following 0, 30, 60, 120, 180 min and 24 h and quenched with a cold solution of reserpine 0.1 mM in acetonitrile. Following centrifugation (10,000 rpm, 10 min, RT), the clear supernatants were collected and analyzed by LC-ESI(+)-MS/MS. Additional control assays were conducted using PBS pH 7.4 instead of plasma solution and using procaine as a positive control.

#### 3.2.2. Human Microsomes Incubations

For the generation of Phase I metabolites: tested compounds, at a 10 μM concentration (1 μL of 5 mM DMSO stock solution), were incubated with HLM (1 mg protein/mL), Vivid^®^ regenerating system (5 μL) and NADPH (1mM) in ABIC 50 mM buffer pH 7.4, for a total incubation volume of 500 μL. Incubations were run in duplicate, at 37 °C. Aliquots were collected at 0, 5, 10, 20, 30, 40, 50, 60, 75, 90, 120, 180 min and reactions were stopped by adding and ice-cold solution of reserpine (2.5 μM) in acetonitrile. Additional aliquots were taken at 0 and 120 min and subsequently treated with *L*-lysine (100 µM) and sodium cyanoborohydride (10 µM) or glutathione (100 µM). Following centrifugation (10,000 rpm, 15 min, RT), the clear supernatants were collected and analyzed LC-ESI-HRMS/MS. Control incubations were conducted in the same conditions: (1) using DMSO as negative control, in absence of the test drug; (2) using heat-denatured HLM (90 °C, 15 min); (3) using nevirapine, as a positive control, instead of the tested compound; and (4) in absence of NADPH co-factor.

For the generation of glucuronidation metabolites: HLM were preincubated for 15 min with alamethicin (25 µg/mL). Incubations were then run in duplicate at 37 °C, using the following conditions: test compounds at a concentration of 25 µM (1 μL of DMSO stock solution 5 mM), HLM (1 mg/mL, 10 μL), NADPH (5 mM), MgCl_2_ (2 mM), UDPGA (5 mM) for a final volume of 200 μL, in 50 mM ABIC buffer pH 7.4. Control incubations were conducted in the same conditions using: (1) DMSO as negative control, in absence of the test compound; (2) heat-denatured HLM (90 °C, 15 min); and (3) in absence of UDPGA co-factor. Aliquots (50 μL) were collected at 0 and 2 h and then quenched with 50 μL of a cold 2.5 μM reserpine solution in acetonitrile. All the samples were vortexed and centrifuged for 15 min at 10,000 rpm. The supernatants were collected and stored at −20 °C until further analysis by LC-ESI-HRMS/MS.

#### 3.2.3. S9 Rat Liver Fraction Incubations

Tested compounds at a 10 μM concentration (1 μL of 5 mM DMSO stock solution) were incubated with rat liver S9 fraction (2 mg protein/mL), Vivid^®^ regenerating system (2 μL) and NADPH (1mM) in ABIC 50 mM buffer pH 7.4, for a total incubation volume of 500 μL. Incubations were run in duplicate at 37 °C. Additional incubation was run using the same conditions in the presence of glutathione (1 mM). Incubations were run in duplicate at 37 °C. Aliquots were collected at 0, 5, 10, 20, 30, 40, 50, 60, 75, 90, 120, 180 min and reactions were terminated by adding an ice-cold solution of reserpine (2.5 μM) in acetonitrile. Following centrifugation (10,000 rpm, 15 min, RT) the clear supernatants were collected and analyzed LC-ESI-HRMS/MS. Control incubations were conducted in the same conditions: (1) using DMSO as negative control, in absence of the test drug; (2) using heat-denatured HLM (90 °C, 15 min); (3) using nevirapine, as a positive control, instead of the tested compound; and (4) in absence of NADPH co-factor

### 3.3. In Vitro Reactions with Bionucleophiles

#### 3.3.1. Glutathione Reactions

To a solution of compound **13d** in acetonitrile (1.00 eq., 10.1 mg in 400 µL, 5.04 × 10^−2^ M) reduced glutathione (4.00 eq. 25.2 mg, 82.00 µmol) dissolved in 400 µL of ABIC buffer 50 mM pH 7.4 was added. The reaction mixture was incubated for 24 h at 37 °C and monitored by LC-ESI-HRMS/MS.

To a solution of compound **13k** in acetonitrile (1.00 eq., 4.3 mg in 200 µL, 3.92 × 10^−2^ M) reduced glutathione (4.00 eq. 9.64 mg, 31.36 µmol) dissolved in 200 µL of ABIC buffer 50 mM pH 7.4 was added. The reaction mixture was incubated for 24 h at 37 °C and monitored by LC-ESI-HRMS/MS.

#### 3.3.2. *N*-Acetyl Cysteine Reactions

To a solution of compound **13d** in acetonitrile (1.00 eq., 10.3 mg in 400 µL, 5.14 × 10^−2^ M) *N*-acetyl cysteine (4.00 eq. 14.0 mg, 85.79 µmol) dissolved in 400 µL of ABIC buffer 50 mM pH 7.4 was added. The reaction mixture was incubated for 24 h at 37 °C and monitored by LC-ESI-HRMS/MS.

To a solution of compound **13k** in acetonitrile (1.00 eq., 7.6 mg in 400 µL, 3.46 × 10^−2^ M) *N*-acetyl cysteine (4.00 eq. 9.70 mg, 59.44 µmol) dissolved in 400 µL of ABIC buffer 50 mM pH 7.4 was added. The reaction mixture was incubated for 24 h at 37 °C and monitored by LC-ESI-HRMS/MS.

#### 3.3.3. *L*-Lysine Reactions

**SLMP53-1_M5** (1.00 eq., 2.1 mg, dissolved in 150 µL of ACN) was added to a *L*-lysine (2 eq.) solution in 50 mM ABIC buffer pH 7.4. Following incubation for 24 h at 37 °C a solution of cyanoborohydride (2 eq) was added. The mixture was analyzed by LC-ESI(+)-HRMS/MS.

### 3.4. Pharmacokinetics Determination

#### 3.4.1. Half-Life Determination and Intrinsic Clearance Calculation

The in vitro depletion half-lives of tested compounds *t*_1/2_ were calculated using Equation (1), assuming that testing compounds follow a first-order kinetic trend. The “slope” was determined from linear fitting of the natural logarithm of the concentration of drug remaining plotted against time:

(1) $$t_{1/2}=\frac{ln2}{slope}$$

The intrinsic clearance in HLM (CLint, HLM) was calculated using Equation (2) [32,50]:

(2) $$CL_{int\;HLM\;}=\frac{ln2}{\;t_{\frac{1}{2}}\;}\;x\;\frac{mL\;incubation}{mg\;microsomes}\;x\;\frac{45\;mg\;microsomes}{g\;liver}\;x\;\frac{26\;g\;liver}{Kg\;b.w.}$$

#### 3.4.2. Data Processing and Metabolite Identification

HLM incubations and rat S9 mix fractions incubations were analyzed by LC-HRMS/MS. All spectra corresponding to metabolites were then manually checked. Signals corresponding to ions identifying the appropriate (*S*)-tryptophanol-derived isoindolinone and Phase I and II metabolites were extracted from raw data by use of DataAnalysis program (Bruker Daltoniks, Mannheim, Germany). HPLC-MS signals of identified metabolites were also measured on the basis of chromatographic peak area normalized to the signal from an internal standard. The exact mass of each [M+H]^+^ or [M-H]^-^ precursor and products ions were determined in accordance with a mass deviation from the accurate mass below ±5 ppm. After their detection, structural characterization of the potential metabolites was achieved by processing tandem MS/MS spectra.

### 3.5. General Procedure for the Synthesis of SLMP53-1 Phase I Metabolites

To a solution of (*S*)-tryptophanol-derived oxazoloisoindolinone SLMP53-1 (**6**) (1.00 eq., 100.2 mg, 314.1 μmol) and White–Chen catalyst (**8**) (10% mol, 29.3 mg) in acetonitrile (5 mL) acetic acid (5.00 eq., 89.8 μL, 1.57 mmol) was added. Hydrogen peroxide 35% wt was added (0.45 eq, 141.3 μmol, 12.2 μL) and the reaction mixture stirred at 40 °C. The reaction mixture was treated with sodium bisulfite (5 mL, saturated aqueous solution) and ethyl acetate (2.5 mL). The organic fraction was washed sequentially with an aqueous saturated solution of sodium hydrogen carbonate, water and brine (1 × 5 mL of each solution), dried with anhydrous sodium sulfate and concentrated under reduced pressure [30].

**5h reaction**: Starting with SLMP53-1 (**6**) (1.00 eq., 140.0 mg, 439.73 μmol), two major products were isolated by preparative TLC with dichloromethane/methanol (from 1 to 6% of MeOH): SLMP53-1 (**6**) 11.0 mg, yield 7.8%; **SLMP53-1_M3a** and **SLMP53-1_M3b** 17.6 mg, yield 13%; **SLMP53-1_M5** 15.80 mg, yield 11%.

**24 h reaction**: Starting with SLMP53-1 (**6**) (1.00 eq., 100.2 mg, 314.1 μmol), three major products were isolated by preparative TLC with dichloromethane/methanol (from 2 to 6% of MeOH): SLMP53-1 (**6**) 12.3 mg, yield 12.3%; **SLMP53-1_M4** 12.4 mg, yield: 13%; **SLMP53-1_M5** 25.1 mg, yield 26%; **10** 20.8 mg, yield 24%.

**(3*S*,9b*R*)-9b-methyl-3-(((*S*)-2-oxoindolin-3-yl)methyl)-2,3-dihydrooxazolo[2,3-*a*]isoindol-5(9b*H*)-one** and **(3*S*,9b*R*)-9b-methyl-3-(((*R*)-2-oxoindolin-3-yl)methyl)-2,3-dihydrooxazolo[2,3-*a*]isoindol-5(9b*H*)-one, SLMP53-1_M3a** and **SLMP53-1_M3b.** White powder. ^1^H NMR (500 MHz, CDCl_3_) δ_H_ 7.97 (d, *J* = 7.4 Hz, 1H, H-7 oxind. major and minor), 7.75 (d, *J* = 7.5 Hz, 1H, H-9 major), 7.72 (d, *J* = 7.6 Hz, 1H, H-9 minor), 7.66–7.62 (m, 1H, H-8 major and minor), 7.60 (d, *J* = 7.3 Hz, 1H, H-6 minor), 7.57 (d, *J* = 7.4 Hz, 1H, H-6 major), 7.54 (d, *J* = 7.5 Hz, 1H, H-7 major), 7.51 (d, *J* = 8.1 Hz, 1H, H-7 minor), 7.23 (d, *J* = 7.8 Hz, 1H, H-6 oxind. major and minor), 7.09 (t, *J* = 7.5 Hz, 1H, H-5 oxind. major), 7.03 (t, *J* = 7.5 Hz, 1H, H-5 oxind. minor), 6.90 (d, *J* = 8.0 Hz, 1H, H-4 oxind. major and minor), 4.63–4.56 (m, 1H, H-3 major), 4.52–4.43 (m, 2H, H-3 minor, H-2 minor), 4.40 (dd, *J* = 8.6, 7.5 Hz, 1H, H-2, major), 4.06 (m, 2H, H-2 minor and major), 3.82 (m, 1H, H-3 oxind.), 2.60–2.52 (m, 1H, CH_2_-oxind., major), 2.49–2.41 (m, CH_2_-oxind., minor), 1.95–1.91 (m, CH_2_-oxind., minor), (m, 1H, CH_2_-oxind., major), 1.89 (s, 3H, CH_3_ major), 1.84 (s, 1H, CH_3_ minor). ^13^C NMR (126 MHz, CDCl_3_) δ_C_ 179.58 (C-2 oxind. minor), 179.57 (C-2 oxind. major), 175.2 (C-5 isoind. major), 174.3 (C-5 isoind. minor), 147.7 (C-9a isoind., major), 147.4 (C-9a isoind., minor), 141.7 (C-7a oxind., minor), 141.2 (C-7a oxind., major), 133.7 (C-8 isoind., major), 133.5 (C-8 isoind., minor), 131.4 (C-5a isoind., minor), 131.4 (C-5a isoind., major), 130.4 (C-7 isoind., major), 130.3 (C-7 isoind., minor), 129.9 (C-3a oxind., major), 129.2 (C-3a oxind., minor), 128.3 (C-6 oxind., minor), 128.2 (C-6 oxind., major), 126.1 (C-7 oxind.), 124.5 (C-9 isoind., minor), 124.4 (C-9 isoind., major), 122.8 (C-5 oxind., major), 122.6 (C-5 oxind., minor), 122.4 (C-6 isoind., major), 122.3 (C-6 isoind., minor), 109.9 (C-4 oxind., minor), 109.7 (C-4 oxind., major), 99.3 (C-9b, major), 99.2 (C-9b, minor), 75.2 (C-2 isoind., minor), 75.0 (C-2 isoind., major), 54.3 (C-3, major), 53.6 (C-3, minor), 44.07 (C-3 oxind., minor), 43.97 (C-3 oxind., major), 37.1 (CH_2_-oxind., major), 37.0 (CH_2_-oxind., minor), 23.5 (CH_3_, major), 23.2 (CH_3_, minor). Compounds were separated with preparative HPLC acetonitrile/water 55:45: SLMP53-1_M3a, retention time 59.7 min and SLMP53-1_M3b, retention time 54.2 min. LC-ESI(+)-HRMS calc. for C_20_H_18_N_2_O_3_ [M+H]^+^ *m*/*z* 335.1390. For SLMP53-1_M3a found *m*/*z* 335.1390 +0.0 ppm. Retention time 9.3 min. For SLMP53-1_M3b found *m*/*z* 335.1396 +1.8 ppm, retention time 9.2 min.

**(3*S*,9b*R*) 3-((3-hydroxy-2-oxoindolin-3-yl)methyl)-9b-methyl-2,3-dihydrooxazolo[2,3-*a*]isoindol-5(9b*H*)-one, SLMP53-1_M4.** White solid. ^1^H NMR (500 MHz, CDCl_3_) δ_H_ 8.07 (s, 1H, NH), 7.78 (d, *J* = 7.6 Hz, 1H, H-7 oxind.), 7.76 (d, *J* = 7.4 Hz, 1H, H-9 isoind.), 7.64 (t, *J* = 7.6 Hz, 1H, H-8 isoind.), 7.55 (d, *J* = 7.3 Hz, 2H, H-7, H-6 isoind.), 7.24 (d, *J* = 7.7 Hz, 1H, H-6 oxind.), 7.07 (t, *J* = 7.6 Hz, 1H, H-5 oxind.), 6.88 (d, *J* = 7.9 Hz, 1H, H-4 oxind.), 5.36 (br s,1H, -OH), 4.50 (dd, *J* = 15.4, 6.9 Hz, 1H, H-2), 4.48–4.42 (m, 1H, H-3), 4.08 (dd, J = 8.6, 7.2 Hz, 1H, H-2), 2.56 (dd, *J* = 14.2, 9.5 Hz, 1H, H-10 or CH_2_-oxind.), 1.94 (dd, *J* = 14.3, 3.1 Hz, 1H, H-10 or CH_2_-oxind.), 1.80 (s, 3H, CH_3_); ^13^C NMR (126 MHz, CDCl_3_) δ_C_ 178.8 (C-2 oxind.), 175.7 (C-5 isoind.), 147.3 (C-9a), 140.20 (C-7a oxind.), 133.9 (C-8 isoind.), 131.4 (C-5a isoind.), 131.2 (C-3a oxind.), 130.6 (C-7 isoind.), 129.7 (C-6 oxind.), 125.5 (C-7 oxind.), 124.7 (C-9 isoind.), 123.10 (C-5 oxind.), 122.4 (C-6 isoind.), 110.5 (C-4 oxind.), 99.8 (C-9b), 75.8 (C-3, oxind.), 75.5 (C-2 isoind.), 51.2 (C-3), 42.8 (C-10 or CH_2_-oxind.), 22.5 (CH_3_). LC-ESI(+)-HRMS calc. for C_20_H_18_N_2_O_4_ [M+H]^+^ *m*/*z* 351.1339. Found *m*/*z* 351.1342 + 0.8 ppm. Retention time 8.6 min.

***N*-(2-(2-((3*S*,9b*R*)-9b-methyl-5-oxo-2,3,5,9b-tetrahydrooxazolo[2,3-*a*]isoindol-3-yl)acetyl)phenyl)formamide, SLMP53-1_M5.** White solid. ^1^H NMR (500 MHz, CDCl_3_) δ_H_ 11.47 (br s, 1H, NH), 8.78 (d, *J* = 8.4 Hz, 1H, H-12), 8.51 (s, 1H, CHO), 7.98 (d, *J* = 8.0 Hz, 1H, H-15), 7.77 (d, *J* = 7.5 Hz, 1H, H-9), 7.66–7.52 (m, 4H, H-8, H-7 and H-6 isoind., H-13), 7.20 (t, *J* = 7.6 Hz, 1H, H-14), 4.77 (dd, *J* = 6.9, 2.5 Hz, 1H, H-2), 4.67–4.60 (m, 1H, H-3), 4.11 (dd, *J* = 6.9, 2.5 Hz, 1H, H-2), 3.98 (dd, *J* = 17.6, 3.7 Hz, 1H, CH_2_-ind.), 3.38 (dd, *J* = 17.6, 10.2 Hz, 1H, CH_2_-ind.), 1.79 (s, 3H, CH_3_); ^13^C NMR (126 MHz, CDCl_3_) δ_C_ 202.3 (C=O), 174.3 (C=O), 160.0 (C=O), 147.2 (C-9a isoind.), 140.2 (C-11b), 135.8 (C-13), 133.6 (C-8 isoindol.), 131.5 (C-5a isoind.), 131.1 (C-15), 130.5 (C-7 isoindol.), 127.3 (C-11a), 124.6 (C-9 isoindol.), 123.4 (C-6 isoindol.), 122.4 (C-14), 122.0 (C-12), 98.7 (C-9b), 75.9 (C-2), 52.3 (C-3), 46.2 (C-10), 23.0 (CH_3_). LC-ESI(+)-HRMS calc. for C_20_H_18_N_2_O_4_ [M+H]^+^ *m*/*z* 351.1339. Found *m*/*z* 351.1348 + 2.6 ppm. Retention time: 9.4 min.

**(7*S*,13b*S*)-7-(hydroxymethyl)-13b-methyl-7,8,13,13b-tetrahydro-5*H*-benzo[1,2]indolizino[8,7-*b*]indol-5-one, 10.** Yellow powder. ^1^H NMR (500 MHz, CDCl_3_) δ_H_ 8.20 (s, 1H, NH), 7.88 (d, *J* = 7.5 Hz, 1H, H-1), 7.77 (d, *J* = 7.7 Hz, 1H, H-4), 7.66 (td, *J* = 7.6, 0.9 Hz, 1H, H-3), 7.50 (t, *J* = 7.6 Hz, 1H, H-11), 7.47 (d, *J* = 8.0 Hz, 1H, H-12), 7.37 (d, *J* = 8.1 Hz, 1H, H-9), 7.19 (t, *J* = 7.5 Hz, 1H, H-10), 7.12 (t, *J* = 7.5 Hz, 1H, H-2), 5.15 (m, 1H, H-7), 3.94 (dd, *J* = 7.6, 2.2 Hz, 1H, CH_2_-OH), 3.14 (dd, *J* = 16.0, 7.5 Hz, 1H, H-8), 2.87 (dd, *J* = 16.0, 2.6 Hz, 1H, H-8), 1.92 (s, 3H, CH_3_); ^13^C NMR (126 MHz, CDCl_3_) δ_C_ 170.5 (C-5), 149.1 (C-4a), 136.5 (C-12a), 133.5 (C-4b), 132.8 (C-3), 130.5 (C-13a), 129.0 (C-11), 127.0 (C-8b), 124.8 (C-1), 122.9 (C-10), 121.1 (C-4), 120.3 (C-2), 118.9 (C-12), 111.3 (C-9), 106.7 (C-8a), 64.3 (CH_2_-OH), 62.3 (C-13b), 51.2 (C-7), 28.7 (CH_3_), 22.3 (C-8). LC-ESI(+)-HRMS calc. for C_20_H_18_N_2_O_2_ [M+H]^+^ *m*/*z* 319.1441. Found *m*/*z* 319.1443 + 0.6 ppm. Retention time 8.6 min [37].

### 3.6. Synthesis of Bromo-Enriched Tryptophanol-Derived Isoindolinones ***13***a-o and ***13***a′

Under nitrogen atmosphere and at 0 °C, a solution of pyridinium tribromide (PyHBr_3_) (1.2 to 1.6 eq., 0.15 M) in tetrahydrofuran (THF) was added dropwise to a solution of the appropriate tryptophanol-derived isoindolinone (1.0 eq., 0.15 M) in dichloromethane (DCM). Aqueous saturated solution of sodium thiosulfate (Na_2_S_2_O_3_) and aqueous saturated solution of sodium hydrogen carbonate (NaHCO_3_) (0.6 mL) were added, and the aqueous layer was extracted with DCM. The combined organic extracts were dried, filtered, and concentrated under reduced pressure. Flash chromatography (FC) or preparative thin layer chromatography (PTLC) (*n*-hexane/ethyl acetate) afforded the corresponding 2-bromoindole derivative.

**(3*S*, 9b*R*)-3-((2-bromo-1*H*-indol-3-yl)methyl)-9b-phenyl-2,3-dihydrooxazolo[2,3-*a*]isoindol-5(9b*H*)-one, 13a.** Following the general PyHBr_3_-bromination procedure, PyHBr_3_ (514.6 mg, 1.60 mmol, 1.6 eq.) dissolved in 10.6 mL of anhydrous THF was added dropwise to (3*S*, 9b*R*)-3-((1*H*-indol-3-yl)methyl)-9b-phenyl-2,3-dihydrooxazolo[2,3-*a*]isoindol-5(9b*H*)-one (**11a**) (381.4 mg, 1.00 mmol, 1.0 eq.) dissolved in 10.7 mL of anhydrous DCM. Reaction time: 10 s. Eluent for FC: ethyl acetate/*n*-hexane, 4:6. Recrystallization in DCM/*n*-hexane. Bromo derivative **13a** (409.7 mg, yield 89%) was obtained as yellow light solid. Mp: 207–209 °C. [α]^20^_D_ = +98.0° (c = 0.39, CH_2_Cl_2_). ^1^H NMR (400 MHz, CDCl_3_) δ_H_ 8.70 (br s, 1H, NH), 7.80 (dd, *J* = 5.7, 2.8 Hz, 1H, H-9 isoind.), 7.64 (d, *J* = 6.7 Hz, 2H, H-2 and H-6, 9b-phenyl), 7.45 (dt, *J* = 7.4, 6.8 Hz, 3H, H-7 ind., H-7 and H-8 isoind.), 7.40 (m, 3H, H-3, H-4 and H-5, 9b-phenyl), 7.22 (m, 2H, H-5 ind. and H-6 isoind.), 7.12 (t, *J* = 7.3 Hz, 1H, H-4 ind.), 7.06 (t, *J* = 7.4 Hz, 1H, H-6 ind.), 4.79–4.66 (m, 1H, H-3), 4.33 (t, *J* = 8.2 Hz, 1H, H-2), 4.05 (t, *J* = 8.2 Hz,1H, H-2), 3.20 (dd, *J* = 14.2, 4.9 Hz, 1H, CH_2_-ind.), 2.70 (dd, *J* = 14.2, 10.1 Hz, 1H, CH_2_-ind.); ^13^C NMR (101 MHz, CDCl_3_) δ_C_ 174.8 (C=O), 147.3 (C-9a isoind.), 138.6 (C-1, 9b-phenyl), 136.2 (C-7a ind.), 133.5 (C-8 isoind.), 131.1 (C-5a isoind.), 130.2 (C-7 isoind.), 128.8 (C-3 and C-5, 9b-phenyl), 128.7 (C-4, 9b-phenyl), 127.7 (C-3a ind.), 126.0 (C-2 and C-6, 9b-phenyl), 124.5 (C-9 isoind.), 123.6 (C-6 isoind.), 122.4 (C-5 ind.), 120.2 (C-6 ind.), 118.2 (C-7 ind.), 111.0 (C-3 ind.), 110.7 (C-4 ind.), 109.0 (C-Br), 101.1 (C-9b), 75.7 (C-2), 55.6 (C-3), 29.4 (CH_2_-ind.). MS-ESI(+) C_25_H_19_BrN_2_O_2_ [M+H]^+^. Found *m*/*z* 459. Retention time: 7.5 min.

**(3*S*, 9b*R*)-3-((2-bromo-1-methyl-1*H*-indol-3-yl)methyl)-9b-phenyl-2,3-dihydrooxazolo[2,3-*a*]isoindol-5(9b*H*)-one, 13c.** Following the general PyHBr_3_-bromination procedure, PyHBr_3_ (73.1 mg, 227.85 µmol, 1.6 eq.) dissolved in 1.52 mL of dry THF was added dropwise to small molecule SLMP53-2 (**7**) (53.8 mg, 136.38 µmol, 1.0 eq.) dissolved in 1.00 mL of anhydrous DCM. Reaction time: 10 s. Eluent for flash chromatography: ethyl acetate/*n*-hexane, 4:6. Recrystallization in DCM/*n*-hexane. Bromo derivative **13c** (59.1 mg, 92%) was obtained as off-white solid. Mp: 125–127 °C. [α]^20^_D_ = +91.0° (c = 0.40, CH_2_Cl_2_). ^1^H NMR (300 MHz, CDCl_3_) δ_H_ 7.83–7.76 (m, 1H, H-9 isoind.), 7.61–7.56 (m, 2H, H-2 and H-6, 9b-phenyl), 7.51 (d, *J* = 7.7 Hz, 1H, ArH, H-7 ind.), 7.47 (dd, *J* = 5.6, 3.1 Hz, 2H, H-7 and H-8 isoind.), 7.41–7.35 (m, 3H, ArH, H-3, H-4 and H-5, 9b-phenyl), 7.25–7.16 (m, 3H, ArH, H-6 isoind. and H-4 and H-5 ind.), 7.10 (ddd, *J* = 8.1, 6.4, 1.8 Hz, 1H, H-6 ind.), 4.71 (dtd, *J* = 10.3, 7.3, 4.7 Hz, 1H, H-3), 4.33 (dd, *J* = 8.8, 7.5 Hz, 1H, H-2), 4.05 (dd, *J* = 8.8, 7.2 Hz, 1H, H-2), 3.67 (s, 3H, N-CH_3_), 3.23 (dd, *J* = 14.2, 4.6 Hz, 1H, CH_2_-ind.), 2.77 (dd, *J* = 14.3, 10.2 Hz, 1H, CH_2_-ind.);^13^C NMR (75 MHz, CDCl_3_) δ_C_ 174.8 (C=O), 147.4 (C-9a, isoind.), 138.8 (C-1, C9b-phenyl), 136.9 (C-7a ind.), 133.4 (C-8 isoind.), 131.2 (C-5a isoind.), 130.2 (C-7 isoind.), 128.8 (C-3 and C-5, C9b-phenyl), 128.6 (C-4, C9b-phenyl), 127.3 (C3a ind.), 126.0 (C-2 and C-6, C9b-phenyl), 124.5 (C-9 isoind.), 123.6 (C-6 isoind.), 122.1 (C-5 ind.), 119.9 (C-7 ind.), 118.5 (C-6 ind.), 114.1 (C-Br), 110.2 (C-3 ind.), 109.4 (C-4 ind.), 101.0 (C-9b), 75.6 (C-2), 55.7 (C-3), 31.5 (CH_3_), 29.8 (CH_2_-ind.). MS-ESI(+) C_26_H_21_BrN_2_O_2_ [M+H]^+^. Found *m*/*z* 473. Retention time: 9.7 min.

**(3*S*, 9b*R*)-3-((2-bromo-1-propyl-1*H*-indol-3-yl)methyl)-9b-phenyl-2,3-dihydrooxazolo[2,3-*a*]isoindol-5(9b*H*)-one, 13d.** Following the general PyHBr_3_-bromination procedure, PyHBr_3_ (56.4 mg, 175.70 µmol, 1.6 eq.) dissolved in 1.17 mL of dry THF was added dropwise to a solution of 0.73 mL anhydrous DCM of small molecule (3*S*, 9b*R*)-9b-phenyl-3-((1-propyl-1*H*-indol-3-yl)methyl)-2,3-dihydrooxazolo[2,3-*a*]isoindol-5(9b*H*)-one (**13b**) (46.4 mg, 109.82 µmol, 1.0 eq.). Reaction time: 10 s. Eluent for flash chromatography: ethyl acetate/*n*-hexane, 2:8. Recrystallization in DCM/*n*-hexane. Bromo derivative **13d** (46.1 mg, 84%) was obtained as yellow pale solid. Mp: 45–47 °C; [α]^20^_D_ = +78.9° (c = 0.40, CH_2_Cl_2_). ^1^H NMR (400 MHz, CDCl_3_) δ_H_ 7.88–7.82 (m, 1H, H-9 isoind.), 7.66 (d, *J* = 6.4 Hz, 2H, H-2 and H-6, 9b-phenyl), 7.57 (d, *J* = 7.7 Hz, 1H, H-7 ind.), 7.51–7.46 (m, 2H, H-7 and H-8 isoind.), 7.41 (m, 3H, H-3, H-4 and H-5, 9b-phenyl), 7.26 (m, 2H, H-4 ind. and H-6 isoind.), 7.23–7.18 (m, 1H, H-5 ind.), 7.13 (t, *J* = 7.1 Hz, 1H, H-6 ind.), 4.86–4.68 (m, 1H, H-3), 4.34 (t, *J* = 8.1 Hz, 1H, H-2), 4.10 (d, *J* = 5.4 Hz, 3H, H-2 and NCH_2_), 3.29 (dd, *J* = 14.2, 4.3 Hz, 1H, CH_2_-ind.), 2.81 (dd, *J* = 13.9, 10.7 Hz, 1H, CH_2_-ind.), 1.77 (sextet, *J* = 7.2 Hz, 2H, CH_2_CH_3_), 0.93 (t, *J* = 7.3 Hz, 3H, CH_2_CH_3_).^13^C NMR (101 MHz, CDCl_3_) δ_C_ 174.6 (C=O), 147.3 (C-9a), 138.8 (C-1, 9b-phenyl), 136.2 (C-7a ind.), 133.3 (C-8 isoind.), 131.2 (C-5a isoind.), 130.1 (C-7 isoind.), 128.7 (C-3 and C-5, 9b-phenyl), 128.6 (C-4, 9b-phenyl), 127.4 (C-3a, ind.), 125.9 (C-2 and C-6, 9b-phenyl), 124.4 (C-9 isoind.), 123.5 (C-6 isoind.), 121.9 (C-5 ind.), 119.8 (C-6 ind.), 118.5 (C-7 ind.), 113.5 (C-Br), 110.1 (C-3 ind.), 109.6 (C-4 ind.), 100.9 (C-9b), 75.5 (C-2), 55.7 (C-3), 46.5 (NCH_2_), 29.8 (CH_2_-ind.), 23.2 (CH_2_CH_3_), 11.4 (CH_2_CH_3_). LC-ESI(+)-HRMS calc. for C_28_H_25_BrN_2_O_2_ *m*/*z* 501.1172 [M+H]^+^. Found *m*/*z* 501.1177 + 1.0 ppm. Retention time: 12.7 min.

**(3*S*, 9b*R*)-3-((2-bromo-1*H*-indol-3-yl)methyl)-9b-(4-methoxyphenyl)-2,3-dihydrooxazolo[2,3-*a*]isoindol-5(9b*H*)-one, 13e.** Following the general PyHBr_3_-bromination procedure, PyHBr_3_ (15.0 mg, 46.8 µmol, 1.6 eq.) dissolved in 0.31 mL of dry THF was added dropwise to small molecule (3*S*, 9b*R*)-3-((1*H*-indol-3-yl)methyl)-9b-(4-methoxyphenyl)-2,3-dihydrooxazolo[2,3-*a*]isoindol-5(9b*H*)-one (**11b**) (12.0 mg, 29.23 µmol, 1.0 eq.) dissolved in 0.20 mL of anhydrous DCM. Reaction time: 10 s. Eluent for flash chromatography: ethyl acetate/*n*-hexane, 1:1. Recrystallization in DCM/*n*-hexane. Bromo derivative **13e** (12.8 mg, 90%) was obtained as white solid. Mp: 70–72 °C. [α]^20^_D_ = +145.3° (c = 0.40, CH_2_Cl_2_). ^1^H NMR (300 MHz, CDCl_3_) δ_H_ 7.96 (br s, 1H, NH), 7.67–7.59 (m, 3H, ArH), 7.48–7.37 (m, 2H, ArH), 7.24 (s, 2H, ArH), 7.18 (d, *J* = 6.0 Hz, 2H, ArH), 6.98 (d, *J* = 8.8 Hz, 2H, ArH), 4.55 (t, *J* = 8.2 Hz, 1H, H-2), 3.98–3.86 (m, 1H, H-3), 3.83 (s, 3H, O-CH_3_, 9b-phenyl), 3.79 (dd, *J* = 8.5, 7.5 Hz, 1H, H-2), 2.74 (dd, *J* = 14.4, 9.6 Hz, 1H, CH_2_-ind.), 2.34 (dd, *J* = 14.4, 2.7 Hz, 1H, CH_2_-ind.). ^13^C NMR (75 MHz, CDCl_3_) δ_C_ 174.8 (C=O), 160.0 (C-4, 9b-phenyl), 147.2 (C9a isoind), 135.5 (C7a ind), 133.5 (C-8 isoind), 131.1 (C5a isoind), 130.9 (C-1, C9b-phenyl), 130.3 (C-7 isoind), 127.7 (C-3a ind), 127.2 (C-2 and C-6, 9b-phenyl), 124.8 (C-9 isoind), 123.6 (C-6 isoind), 122.6 (C-5 ind), 120.5 (C-6 ind), 118.91 (C-7 ind), 114.3 (C-3 and C-5, C9b-phenyl), 110.9 (C-3 ind), 110.7 (C-4 ind), 109.5 (C-Br), 100.0 (C-9b), 75.6 (C-2), 55.6 (C-3), 55.4 (OCH_3_), 29.0 (CH_2_-ind). MS-ESI(+) C_26_H_21_BrN_2_O_3_ [M+H]^+^. Found *m*/*z* 489. Retention time: 8.8 min.

**(3*S*, 9b*R*)-3-((2-bromo-1*H*-indol-3-yl)methyl)-9b-(*p*-tolyl)-2,3-dihydrooxazolo[2,3-*a*]isoindol-5(9b*H*)-one, 13f.** Following the general PyHBr_3_-bromination procedure, PyHBr_3_ (61.05 mg, 190.88 µmol) dissolved in 1.30 mL of anhydrous THF was added dropwise to small molecule (3*S*,9b*R*)-3-((1*H*-indol-3-yl)methyl)-9b-(*p*-tolyl)-2,3-dihydrooxazolo[2,3-*a*]isoindol-5(9b*H*)-one (**11c**) (47.06 mg, 119.30 µmol) dissolved in 1.00 mL of anhydrous DCM. Reaction time: 10 s. Eluent for preparative chromatography: ethyl acetate/*n*-hexane, 2:8. Recrystallization in DCM/*n*-hexane. Bromo derivative **13f** (39.5 mg, 70%) was obtained as white solid. Mp: 137–139 °C. [α]^20^_D_ = +152.3° (c = 0.40, CH_2_Cl_2_). ^1^H NMR (400 MHz, CDCl_3_) δ_H_ 8.00 (br s, 1H, NH), 7.78 (dd, *J* = 5.9, 2.7 Hz, 1H), 7.52 (t, *J* = 8.1 Hz, 2H), 7.48–7.45 (m, 2H), 7.44–7.40 (m, 2H), 7.25–7.18 (m, 3H), 7.17–7.13 (m, 1H), 7.14–7.08 (m, 1H), 4.73–4.64 (m, 1H, H-3), 4.34 (t, *J* = 8.0 Hz, 1H, H-2), 4.05 (dd, *J* = 8.7, 7.1 Hz, 1H, H-2), 3.20 (dd, *J* = 14.3, 4.9 Hz, 1H, CH_2_-ind.), 2.71 (dd, *J* = 14.2, 10.1 Hz, 1H, CH_2_-ind.), 2.39 (s, 3H, CH_3_). ^13^C NMR (75 MHz, CDCl_3_) δ_C_ 174.6 (C=O), 147.3 (C9a isoind.), 138.5 (C-1 *p*-CH_3_ phenyl), 136.1 (C-7a ind), 135.8 (C-4 *p*-CH_3_ phenyl), 133.3 (C-8 isoind.), 131.1 (C-5a isoind.), 130.0 (C-7 isoind.), 129.5 (C-3 and C-5 *p*-CH_3_ phenyl), 127.5 (C-3a ind.), 125.7 (C-2 and C-6 *p*-CH_3_ phenyl), 124.3 (C-9 isoind.), 123.4 (C-6 isoind.), 122.1 (C-5 ind.), 119.4 (C-6 ind.), 118.8 (C-7 ind.), 112.0 (C-3 ind.), 111.1 (C-4 ind.), 109.7 (C-Br) 101.0 (C-9b), 76.4 (C-2), 55.6 (C-3), 30.2 (CH_2_-ind.), 21.2 (CH_3_). MS-ESI(+) r C_26_H_21_BrN_2_O_2_ [M+H]^+^. Found *m*/*z* 473 [M+H]^+^. Retention time: 8.1 min.

**(3*S*, 9b*R*)-3-((2-bromo-1*H*-indol-3-yl)methyl)-9b-(4-fluorophenyl)-2,3-dihydrooxazolo[2,3-*a*]isoindol-5(9b*H*)-one, 13g.** Following the general PyHBr_3_-bromination procedure, PyHBr_3_ (64.4 mg, 200.78 µmol, 1.6 eq.) dissolved in 1.34 mL of dry THF was added dropwise to small molecule (3*S*, 9b*R*)-3-((1*H*-indol-3-yl)methyl)-9b-(4-fluorophenyl)-2,3-dihydrooxazolo[2,3-*a*]isoindol-5(9b*H*)-one (**11d**) (50.0 mg, 125.49 µmol, 1.0 eq.) dissolved in 0.83 mL of anhydrous DCM. Reaction time: 10 s. Eluent for flash chromatography: ethyl acetate/*n*-hexane, 3:7. Recrystallization in DCM/*n*-hexane. Bromo derivative **13g** (44.70 mg, 75%) was obtained as white solid. Mp: 183–185 °C; [α]^20^_D_ = +131.7° (c = 0.15, CH_2_Cl_2_). ^1^H NMR (300 MHz, Acetone-d6) δ_H_ 8.00 (br s, 1H, NH), 7.83–7.77 (m, 1H, ArH), 7.55 (dd, *J* = 3.2, 1.4 Hz, 1H, ArH), 7.54–7.50 (m, 2H, ArH), 7.48 (dd, *J* = 5.6, 3.1 Hz, 2H, ArH), 7.26–7.22 (m, 1H, ArH), 7.21–7.09 (m, 3H, ArH), 7.07–7.00 (m, 2H, ArH), 4.70 (dtd, *J* = 9.8, 7.3, 4.6 Hz, 1H, H-3), 4.36 (dd, *J* = 8.9, 7.5 Hz, 1H, H-2), 4.05 (dd, *J* = 8.9, 7.2 Hz, 1H, H-2), 3.16 (dd, *J* = 14.3, 4.6 Hz, 1H, CH_2_-ind.), 2.76 (dd, *J* = 14.3, 9.8 Hz, 1H, CH_2_-ind.). ^13^C NMR (75 MHz, Acetone-d6) δ_C_ 174.7 (C=O), 162.0 (d, *J* = 246.4 Hz, C-F), 148.4 (C-9a, isoind), 137.6 (C-7a, ind), 134.3 (C-1, *p*-F-phenyl), 131.9 (C-5a, isoind), 131.2 (C-7, isoind), 129.0 (d, *J* = 8.5 Hz, C-2 and C-6, *p*-F-phenyl), 128.7 (C-3a, ind), 124.8 (C-8, isoind), 124.5 (C-6, isoind), 122.9 (C-5, ind), 120.6 (C-6, ind), 118.8 (C-7, ind), 116.2 (d, *J* = 21.8 Hz, C-3 and C-5, *p*-F-phenyl), 111.7 (C-4, ind), 111.3 (C-3, ind), 109.8 (C-Br), 101.4 (C-9b), 75.9 (C-2, isoind), 57.1 (C-3), 29.3 (CH_2_-ind). MS-ESI(+) C_25_H_18_BrFN_2_O_2_ [M+H]^+^. Found *m*/*z* 477. HPLC retention time: 8.5 min.

**(3*S*, 9b*R*)-3-((2-bromo-1*H*-indol-3-yl)methyl)-9b-(4-chlorophenyl)-2,3-dihydrooxazolo[2,3-*a*]isoindol-5(9b*H*)-one, 13h.** Following the general PyHBr_3_-bromination procedure, PyHBr_3_ (154.4 mg, 481.3 µmol, 1.6 eq.) dissolved in 2.57 mL of anhydrous THF was added dropwise to small molecule (3*S*, 9b*R*)-3-((1*H*-indol-3-yl)methyl)-9b-(4-chlorophenyl)-2,3-dihydrooxazolo[2,3-*a*]isoindol-5(9b*H*)-one (**11e**) (124.8 mg, 300.80 µmol, 1.0 eq.) dissolved in 1.43 mL of anhydrous DCM. Reaction time: 10 s. Eluent for flash chromatography: ethyl acetate/*n*-hexane, 3:7. Recrystallization in DCM/*n*-hexane. Bromo derivative **13h** (132mg, 89%) was obtained as white solid. Mp: 75–77 °C; [α]^20^_D_ = +145.2° (c = 0.15, CH_2_Cl_2_).^1^H NMR (400 MHz, CDCl_3_) δ_H_ 8.22 (br s, 1H, NH), 7.83–7.78 (m, 1H, H-9 isoind.), 7.54 (d, *J* = 6.8 Hz, 1H, H-7 ind.), 7.50–7.46 (m, 2H, H-7 and H-8 isoind.), 7.46 (d, *J* = 8.6 Hz, 2H, H-3 and H-5, 9b-phenyl), 7.30 (d, *J* = 8.5 Hz, 2H, H-2 and H-6, 9b-phenyl), 7.24 (d, *J* = 7.9 Hz, 2H, H-4 ind.), 7.17 (d, *J* = 7.5 Hz, H-6 isoind.), 7.15 (t, *J* = 6.4 Hz, 1H, H-5 ind.), 7.11 (t, *J* = 7.5 Hz, 1H, H-6 ind.), 4.75–4.63 (m, 1H, H-3), 4.38 (dd, *J* = 8.8, 7.5 Hz, 1H, H-2), 4.06 (dd, *J* = 8.9, 7.3 Hz, 1H, H-2), 3.14 (dd, *J* = 14.3, 4.5 Hz, 1H, CH_2_-ind.), 2.78 (dd, *J* = 14.3, 9.6 Hz, 1H, CH_2_-ind.). ^13^C NMR (101 MHz, CDCl_3_) δ_C_ 174.8 (C=O), 147.1 (C-9a, isoind.), 137.5 (C-1, 9b-phenyl), 136.2 (C-7a ind.), 134.5 (C-Cl, 9b-phenyl), 133.6 (C-8 isoind.), 131.1 (C-5a isoind.), 130.4 (C-7 isoind.), 128.9 (C-2 and C-6, 9b-phenyl), 127.9 (C-3a, ind.), 127.4 (C-3 and C-5, 9b-phenyl), 124.6 (C-9 isoind.), 123.5 (C-6 isoind.), 122.6 (C-5 ind.), 120.4 (C-6 ind.), 118.5 (C-7 ind.), 111.2 (C-3 ind.), 110.6 (C-4 ind.), 109.0 (C-Br), 100.7 (C-9b), 75.6 (C-2), 56.1 (C-3), 29.1 (CH_2_-ind.). MS-ESI(+) C_25_H_18_BrClN_2_O_2_ [M+H]^+^. Found *m*/*z* 493. Retention time: 5.8 min.

**(3*S*, 9b*R*)-3-((2-bromo-1*H*-indol-3-yl)methyl)-9b-(4-chloro-3-nitrophenyl)-2,3-dihydrooxazolo[2,3-*a*]isoindol-5(9b*H*)-one, 13i.** Following the general PyHBr_3_-bromination procedure, PyHBr_3_ (57.0 mg, 177.85 µmol, 1.6 eq.) dissolved in 1.19 mL of anhydrous THF was added dropwise to small molecule (3*S*, 9b*R*)-3-(1*H*-indol-3-yl)methyl)-9b-(4-chloro-3-nitrophenyl)-2,3-dihydrooxazolo[2,3-*a*]isoindol-5(9b*H*)-one (**11f**) (51.1 mg, 111.1 µmol, 1.0 eq.) dissolved in 0.74 mL of anhydrous DCM. Reaction time: 10 s. Eluent for flash chromatography: ethyl acetate/*n*-hexane, 3:7. Recrystallization in DCM/*n*-hexane. Bromo derivative **13i** (47.6 mg, 80%) was obtained as off-white solid. Mp: 75–77 °C. [α]^20^_D_ = +65.1° (c = 0.40, CH_2_Cl_2_). ^1^H NMR (400 MHz, CDCl_3_) δ_H_ 8.09 (br s, 1H, NH), 7.86 (d, *J* = 1.8 Hz, 1H, H-2, 9b-phenyl), 7.84 (d, *J* = 8.0 Hz, 1H, H-9 isoind.), 7.56–7.46 (m, 4H, H-7 ind., H-7 and H-8 isoind, H-6 9b-phenyl), 7.19 (dd, *J* = 8.0, 6.1 Hz, 2H, H-4 ind. and H-5, 9b-phenyl), 7.14 (d, *J* = 6.0 Hz, 1H, H-5 ind.), 7.12–7.05 (m, 2H, H-6 isoind. and ind.), 4.70 (qd, *J* = 7.5, 4.4 Hz, 1H, H-3), 4.53 (dd, *J* = 8.9, 7.7 Hz, 1H, H-2), 4.20 (dd, *J* = 8.8, 7.8 Hz, 1H, H-2), 3.07–2.93 (m, 2H, CH_2_-ind.);^13^C NMR (101 MHz, CDCl_3_) δ_C_ 174.9 (C=O), 147.7 (C-NO_2_ 9b-phenyl), 146.5 (C-9a isoind.), 139.8 (C-1 9b-phenyl), 135.9 (C-7a ind.), 133.9 (C-8 isoind.), 131.7 (C-7 isoind.), 131.1 (C-5a isoind.), 130.9 (C-5, 9b-phenyl), 130.4 (C-6, 9b-phenyl), 128.0 (C-3a indole), 126.7 (C-Cl), 124.9 (C-9 isoind.), 123.5 (C-6 isoind.), 122.69 (C-5 ind.), 122.67 (C-2, 9b-phenyl), 120.6 (C-6 ind.), 118.4 (C-7 ind.), 110.8 (C-3 ind.), 110.6 (C-4 ind.), 109.7 (C-Br), 100.0 (C-9b), 75.3 (C-2), 57.1 (C-3), 28.0 (CH_2_-ind.). MS-ESI(+) C_25_H_17_BrClN_3_O_4_ [M+H]^+^. Found *m*/*z* 538. Retention time: 6.8 min.

**(3*R*, 9b*S*)-3-((2-bromo-1*H*-indol-3-yl)methyl)-9b-phenyl-2,3-dihydrooxazolo[2,3-*a*]isoindol-5(9b*H*)-one, 13a′.** Following the general PyHBr_3_-bromination procedure, Pyridinium tribromide (1.6 eq., 202.4 mg, 630.83 µmol) dissolved in 4.2 mL of dry THF was added dropwise to (3*R*, 9b*S*)-3-((1*H*-indol-3-yl)methyl)-9b-phenyl-2,3-dihydrooxazolo[2,3-*a*]isoindol-5(9b*H*)-one (**11d** enantiomer) (143.3 mg, 376.66 µmol) dissolved in 2.5 mL of anhydrous DCM. Reaction time: 10 s. Eluent for flash chromatography: ethyl acetate/*n*-hexane, 3:7. Bromo derivative **13a′** (149.7 mg, 87%) was obtained as yellow light solid. Recrystallization in DCM/*n*-hexane. [α]^20^_D_ = −92.7° (c = 0.38, CH_2_Cl_2_). The ^1^H NMR spectrum was found identical to the one of compound **13a.** MS-ESI(+) C_25_H_19_BrN_2_O_2_ [M+H]^+^. Found *m*/*z* 459. Retention time: 7.5 min.

**General procedure for indole nitrogen derivatization.** At 0 °C and under nitrogen atmosphere, sodium hydride (1.5 to 2.0 equiv., dry 95%) was added to a stirred solution of the appropriate tryptophanol-derived isoindolinone (0.32 mmol, 1.0 equiv.) in DMF (2 mL to 3 mL). After 30 min the appropriate substituted halide or acetic anhydride (2.0 equiv.) was added, and the reaction was slowly allowed to warm up to room temperature till total consumption of the starting material. Ethyl acetate was added (10 mL), and the organic phase washed with deionized water (6 × 10 mL) with an aqueous saturated solution of NaHCO_3_ (10 mL) and then with a brine solution (10 mL). The organic phase was dried over Na_2_SO_4_. After removal of volatiles under reduced pressure and purification via flash chromatography, subsequent recrystallization afforded the titled product.

**(3*S*, 9b*R*)-3-((2-bromo-1-ethyl-1*H*-indol-3-yl)methyl)-9b-phenyl-2,3-dihydrooxazolo[2,3-*a*]isoindol-5(9b*H*)-one, 13j.** Following the general procedure: (3*S*, 9b*R*)-3-((2-bromo-1*H*-indol-3-yl)methyl)-9b-phenyl-2,3-dihydrooxazolo[2,3-*a*]isoindol-5(9b*H*)-one (**13a**) (51.0 mg, 111.0 µmol, 1 eq.); 1 mL of DMF; sodium hydride (5.3 mg, 222.1 µmol) and ethyl iodide (17.90 µL, 222.1 µmol, d = 1.94 g/mL, 2 eq.). Reaction time: 1 h. Eluent for flash chromatography: 8:2, *n*-hexane/ethyl acetate. Recrystallization in *n*-hexane/DCM. The product was obtained as white light solid (91.2 mg, 72%). Mp: 52–54 °C.; [α]^20^_D_ = +85.1° (c = 0.39, CH_2_Cl_2_). ^1^H NMR (400 MHz, CDCl_3_) δ_H_ 7.83–7.77 (m, 1H, H-9 isoind.), 7.66–7.56 (m, 2H, H-2 and H-6, 9b-phenyl), 7.52 (d, *J* = 8.0 Hz, 1H, H-7 ind.), 7.47 (dd, *J* = 5.7, 3.0 Hz, 2H, H-7 and H-8 isoind.), 7.41–7.34 (m, 3H, H-3, H-4 and H-5 of 9b-phenyl), 7.24–7.19 (m, 2H, H-6 isoind. and H-4 ind.), 7.15 (dd, *J* = 7.4, 1.3 Hz, 1H, H-5 ind.), 7.12–7.06 (m, 1H, H-6 ind.), 4.76–4.62 (m, 1H, H-3), 4.33 (dd, *J* = 18.0, 8.8 Hz, 1H, H-2), 4.17 (dd, *J* = 7.3 Hz, NCH_2_), 4.04 (dd, *J* = 8.6, 6.9 Hz, 1H, H-2), 3.21 (dd, *J* = 15.5, 11.1 Hz, 1H, CH_2_-ind.), 2.74 (dd, *J* = 24.1, 13.5 Hz, 1H, CH_2_-ind.), 1.30 (d, *J* = 7.2 Hz, 3H, CH_3_). ^13^C NMR (101 MHz, CDCl_3_) δ_C_ 174.8 (C=O), 147.5 (C-9a, isoind.), 138.9 (C-1, 9b-phenyl), 135.8 (C-7a ind.), 133.4 (C-8 isoind.), 131.3 (C-5a isoind.), 130.2 (C-7 isoind.), 128.8 (C-3 and C-5, 9b-phenyl), 128.7 (C-4, 9b-phenyl), 127.6 (C-3a, ind.), 126.0 (C-2 and C-6, 9b-phenyl), 124.5 (C-9 isoind.), 123.6 (C-6 isoind.), 122.0 (C-5 ind.), 119.9 (C-6 ind.), 118.7 (C-7 ind.), 113.0 (C-Br), 110.3 (C-3 ind.), 109.4 (C-4 ind.), 101.1 (C-9b), 75.6 (C-2), 55.7 (C-3), 39.9 (NCH_2_), 29.8 (CH_2_-ind.), 15.1 (CH_3_). MS-ESI(+) C_27_H_23_BrN_2_O_2_ [M+H]^+^. Found *m*/*z* 487. Retention time: 8.6 min.

**(3*S*, 9b*R*)-3-((1-benzyl-2-bromo-1*H*-indol-3-yl)methyl)-9b-phenyl-2,3-dihydrooxazolo[2,3-*a*]isoindol-5(9b*H*)-one, 13k.** Following the general procedure: (3*S*, 9b*R*)-3-((2-bromo-1*H*-indol-3-yl)methyl)-9b-phenyl-2,3-dihydrooxazolo[2,3-*a*]isoindol-5(9b*H*)-one (**13a**) (96.2 mg, 209.4 µmol, 1 eq.), 1 mL of anhydrous DMF, sodium hydride (10.0 mg, 416.7 µmol, 2 eq.) and benzyl bromide (49.8 µL, 418.9 mmol, d = 1.44 g/mL, 2 eq.). Reaction time: 1 h. Eluent for flash chromatography: 7:3, *n*-hexane/ethyl acetate. Recrystallization in *n*-hexane/ethyl acetate. The product was obtained as white light solid (80.1 mg, 70%). Mp: 58–60 °C. [α]^20^_D_ = +80.1° (c = 0.40, CH_2_Cl_2_). ^1^H NMR (300 MHz, CDCl_3_) δ_H_ 7.81 (qd, *J* = 5.6, 3.1 Hz, 1H, H-9 isoind.), 7.65–7.60 (m, 2H, H-2 and H-6, 9b-phenyl), 7.55 (ddd, *J* = 3.4, 2.2, 0.6 Hz, 1H, H-7 ind.), 7.48 (dd, *J* = 5.6, 3.1 Hz, 2H, H-7 and H-8 isoind.), 7.43–7.35 (m, 3H, H-3, H-4 and H-5, isoind.), 7.26–7.23 (m, 2H, H-3 and H-5, benzyl), 7.21 (dd, *J* = 4.3, 2.0 Hz, 1H, H-6 isoind.), 7.21–7.17 (m, 1H, H-4 ind.), 7.17–7.13 (m, 2H, H-5, ind. and H-4 benzyl), 7.12–7.09 (m, 1H, H-6 ind.), 7.00 (dd, *J* = 7.7, 1.7 Hz, 2H, H-2 and H-6, benzyl), 5.34 (s, 2H, CH_2_-benzyl), 4.78–4.69 (m, 1H, H-3), 4.34 (dd, *J* = 8.8, 7.5 Hz, 1H, H-2), 4.07 (dd, *J* = 8.8, 7.0 Hz, 1H, H-2), 3.27 (dd, *J* = 14.2, 4.8 Hz, 1H, CH_2_-ind.), 2.80 (dd, *J* = 14.2, 10.2 Hz, 1H, CH_2_-ind.). ^13^C NMR (75 MHz, CDCl_3_) δ_C_ 174.7 (C=O), 147.5 (C-9a, isoind.), 138.9 (C-1, 9b-phenyl), 137.2 (C-1, benzyl), 136.7 (C-7a ind.) 133.4 (C-8 isoind.), 131.3 (C-5a isoind.), 130.2 (C-7 isoind.), 128.9 (C-3 and C-5 9b-phenyl), 128.8 (C-3 and C-5 benzyl), 128.7 (C-4, 9b-phenyl), 127.7 (C-3a ind.), 127.6 (C-4, benzyl), 126.4 (C-2 and C-6, benzyl), 126.1 (C-2 and C-6, 9b-phenyl), 124.5 (C-9 isoind.), 123.6 (C-6 isoind.), 122.4 (C-5 ind.), 120.3 (C-6 ind.) 118.7 (C-7 ind.), 113.9 (C-Br), 111.1 (C-3 ind.), 110.0 (C-4 ind.), 101.1 (C-9b), 75.6 (C-2), 55.7 (C-3), 48.5 (CH_2_-benzyl), 31.6 (CH_2_-ind.). LC-ESI(+)-HRMS *m*/*z* calcd. for C_28_H_25_BrN_2_O_2_ [M+H]^+^: *m*/*z* 549.1172. Found *m*/*z* 549.1179 +1.3 ppm Retention time: 12.5 min.

**(3*S*, 9b*R*)-3-((1-benzoyl-2-bromo-1*H*-indol-3-yl)methyl)-9b-phenyl-2,3-dihydrooxazolo[2,3-*a*]isoindol-5(9b*H*)-one, 13l.** Following the general procedure: (3*S*, 9b*R*)-3-((2-bromo-1*H*-indol-3-yl)methyl)-9b-phenyl-2,3-dihydrooxazolo[2,3-*a*]isoindol-5(9b*H*)-one (**13a**) (51.7 mg, 112.55 µmol, 1 eq.), 1 mL of anhydrous DMF, sodium hydride (4.50 mg, 168.83 µmol, 1.5 eq.) and benzoyl chloride (25.3 µL, 225.1 µmol, d = 1.21 g/mL, 2 eq.). Reaction time: 1 h. Eluent for flash chromatography: 7:3, *n*-hexane/ethyl acetate. Recrystallization in *n*-hexane/DCM. The product was obtained as white light solid (48.2 mg, 76%). Mp: 65–67 °C. [α]^20^_D_ = +42.1° (c = 0.39, CH_2_Cl_2_). ^1^H NMR (300 MHz, CDCl_3_) δ_H_ 7.84–7.78 (m, 1H, H-9, isoind), 7.70–7.65 (m, 2H, H-2 and H-6 benzoyl), 7.65–7.61 (m, 1H, H-4, benzoyl), 7.61–7.55 (m, 3H, H-7 ind., H-2 and H-6, 9b-phenyl), 7.53–7.44 (m, 4H, H-3 and H-5, benzoyl and H-7 and H-8, isoind.), 7.40–7.32 (m, 4H, H-4, ind. and H-3, H-4 and H-5 C9b-phenyl), 7.25–7.15 (m, 3H, H-5 and H-6 ind. and H-6 isoind.), 4.79–4.68 (m, 1H, H-3), 4.46 (dd, *J* = 8.8, 7.5 Hz, 1H, H-2), 4.10 (dd, *J* = 8.8, 7.4 Hz, 1H, H-2), 3.16 (dd, *J* = 14.2, 5.2 Hz, 1H, CH_2_-ind), 2.88 (dd, *J* = 14.2, 9.1 Hz, 1H, CH_2_-ind). ^13^C NMR (75 MHz, CDCl_3_) δ_C_ 175.0 (C=O), 168.7 (C=O), 147.5 (C-9a, isoind), 138.7 (C-1, C9b-phenyl), 137.5 (C-7a, ind), 134.7 (C-1, benzoyl), 133.6 (C-8, isoind and C-4, benzoyl), 131.1 (C-5a, isoind), 130.6 (C-2 and C-6 benzoyl), 130.3 (C-7, isoind), 128.94 (C-3a, ind), 128.84 (C-3 and C-5, 9b-phenyl and C-4, 9b-phenyl), 128.78 (C-3 and C-5, benzoyl), 126.0 (C-2 and C-6, 9b-phenyl), 124.6 (C-9, isoind), 124.5 (C-6, ind), 123.7 (C-6, isoind), 123.2 (C-5, ind), 119.3 (C-Br), 118.9 (C-7, ind), 113.9 (C-4, ind), 110.6 (C-3, ind), 101.2 (C-9b, isoind), 75.3 (C-2), 55.6 (C-3), 29.5 (CH_2_-ind). MS-ESI(+)C_32_H_23_BrN_2_O_3_ [M+H]^+^. Found *m*/*z* 563. Retention time: 10.6 min.

**(3*S*, 9b*R*)-3-((1-acetyl-2-bromo-1*H*-indol-3-yl)methyl)-9b-phenyl-2,3-dihydrooxazolo[2,3-*a*]isoindol-5(9b*H*)-one, 13m.** Following the general procedure: (3*S*, 9b*R*)-3-((2-bromo-1*H*-indol-3-yl)methyl)-9b-phenyl-2,3-dihydrooxazolo[2,3-*a*]isoindol-5(9b*H*)-one (**13a**) (100.0 mg, 253.5 µmol, 1.00 eq.), 2 mL of DMF, sodium hydride (8.0 mg, 333.4 µmol, 1.5 eq.) and acetic anhydride (41.2 µL, 435.4 µmol, 2.0 eq.). Reaction time: 45 min. Eluent for flash chromatography: 1:1, *n*-hexane/ethyl acetate. Recrystallization in *n*-hexane/DCM. Product **13m** was obtained as white light solid (91mg, 84%). Mp: 110–112 °C; [α]^20^_D_ = +70.3° (c = 0.4, CH_2_Cl_2_). ^1^H NMR (400 MHz, CDCl_3_) δ_H_ 8.21 (d, *J* = 8.0 Hz, 1H, H-4 ind.), 7.82–7.76 (m, 1H, H-9 isoind.), 7.51–7.44 (m, 5H, H-8 and H-7 isoind., H-2 and H-6 9b-phenyl, H-7 ind.), 7.32 (dd, *J* = 5.1, 1.6 Hz, 3H, H-3, H-5 and H-6 9b-phenyl), 7.28 (dd, *J* = 3.6, 1.3 Hz, 1H, H-5 ind.), 7.20–7.17 (m, 1H, H-6 ind.), 4.75–4.66 (m, 1H, H-3), 4.43 (dd, *J* = 10.0, 8.0 Hz, 1H, H-2), 4.10 (dd, *J* = 10.0, 7.6 Hz, 1H, H-2), 3.12 (dd, *J* = 14.3, 4.7 Hz, 1H, CH_2_-ind.), 2.90 (dd, *J* = 14.3, 9.2 Hz, 1H, CH_2_-ind.), 2.77 (s, 3H, CH_3_). ^13^C NMR (101 MHz, CDCl_3_) δ_C_ 175.0 (C=O), 169.9 (C=O), 147.4 (C-9a, isoind.), 138.5 (C-1, 9b-phenyl), 137.3 (C-7a ind.), 133.6 (C-8 isoind.), 131.0 (C-5a isoind.), 130.3 (C-7 isoind.), 129.1 (C-3a, ind.), 128.8 (C-5 and C-3, 9b-phenyl), 128.7 (C-4, 9b-phenyl), 125.9 (C-2 and C-6, 9b-phenyl), 125.6 (C-6 ind.), 124.5 (C-9 isoind.), 123.9 (C-5 ind.), 123.7 (C-6 isoind.), 120.7 (C-3 ind.), 118.6 (C-7 ind.), 116.2 (C-4 ind.), 108.7 (C-Br), 101.2 (C-9b), 75.2 (C-2), 55.4 (C-3), 29.5 (CH_2_-ind.), 28.4 (CH_3_). MS-ESI(+) C_27_H_21_BrN_2_O_3_ [M+H]^+^. Found *m*/*z* 501. Retention time: 5.6 min.

**(3*S*, 9b*R*)-3-((2-bromo-1-(methylsulfonyl)-1*H*-indol-3-yl)methyl)-9b-phenyl-2,3-dihydrooxazolo[2,3-*a*]isoindol-5(9b*H*)-one, 13n.** Following the general procedure: (3*S*, 9b*R*)-3-((2-bromo-1*H*-indol-3-yl)methyl)-9b-phenyl-2,3-dihydrooxazolo[2,3-*a*]isoindol-5(9b*H*)-one (**13a**) (50.0 mg, 108.9 µmol), 1 mL of DMF, sodium hydride (3.92 mg, 163.3 µmol) and methanesulfonyl chloride (16.9 µL, 217.7 mmol, d = 1.48 g/mL). Reaction time: 2 h. Eluent for flash chromatography: 7:3, *n*-hexane/ethyl acetate. Recrystallization in *n*-hexane/DCM. The product was obtained as white solid (48.2 mg, 82%). Mp: 71–73 °C. [α]^20^_D_ = +65.3° (c = 0.4, CH_2_Cl_2_). ^1^H NMR (400 MHz, CDCl_3_) δ_H_ 8.08–8.04 (m, 1H, H-4 ind.), 7.76–7.71 (m, 1H, H-9 isoind.), 7.58–7.52 (m, 2H, H-2 and H-6, 9b-phenyl), 7.50–7.44 (m, 3H, H-7 ind., H-7 and H-8 isoind.), 7.41–7.35 (m, 3H, H-3, H-4 and H-5, 9b-phenyl), 7.33–7.27 (m, 2H, H-5 and H-6 ind.), 7.19 (dd, *J* = 5.6, 2.6 Hz, 2H, H-6 isoind.), 4.73–4.61 (m, 1H, H-3), 4.51 (dd, *J* = 8.8, 7.6 Hz, 1H, H-2), 4.08 (dd, *J* = 9.0, 7.6 Hz, 1H, H-2), 3.12 (s, 3H, CH_3_), 3.03 (dd, *J* = 14.2, 6.3 Hz, 1H, CH_2_-ind.), 2.85 (dd, *J* = 14.3, 7.6 Hz, 1H, CH_2_-ind.). ^13^C NMR (101 MHz, CDCl_3_) δ_C_ 174.9 (C=O), 147.4 (C-9a, isoind.), 138.8 (C-1, 9b-phenyl), 136.2 (C-7a ind.), 133.5 (C-8 isoind.), 131.2 (C-5a isoind.), 130.2 (C-7 isoind.), 128.9 (C-3 and C-5, 9b-phenyl), 128.8 (C-4, 9b-phenyl), 127.8 (C-3a, ind.), 126.0 (C-2 and C-6, 9b-phenyl), 125.4 (C-6 ind.), 124.5 (C-5 ind.), 123.6 (C-6 isoind.), 119.1 (C-7 ind.), 115.2 (C-4 ind.), 111.4 (C-3 ind.), 108.9 (C-Br), 101.2 (C-9b), 75.7 (C-2), 55.6 (C-3), 41.5 (CH_3_), 29.5 (CH_2_-ind.). MS-ESI(+)C_26_H_21_BrN_2_O_4_S [M+H]^+^. Found *m*/*z* 537. Retention time: 5.0 min.

**(3*S*, 9b*R*)-3-((2-bromo-1-tosyl-1*H*-indol-3-yl)methyl)-9b-phenyl-2,3-dihydrooxazolo[2,3-*a*]isoindol-5(9b*H*)-one, 13o.** Following the general procedure: (3*S*, 9b*R*)-3-((2-bromo-1*H*-indol-3-yl)methyl)-9b-phenyl-2,3-dihydrooxazolo[2,3-*a*]isoindol-5(9b*H*)-one (**13a**) (47.6 mg, 103.6 µmol), 1 mL of anhydrous DMF, sodium hydride (3.73 mg, 155.4 µmol) and *p*-toluenesulfonyl chloride (41.5 mg, 217.7 mmol). Reaction time: 2.5 h. Eluent for flash chromatography: 8:2, *n*-hexane/ethyl acetate. Recrystallization in *n*-hexane/DCM. The product was obtained as white solid (39.6 mg, 62%). Mp: 192–194 °C. [α]^20^_D_ = +65.3° (c = 0.4, CH_2_Cl_2_). ^1^H NMR (400 MHz, CDCl_3_) δ_H_ 8.24 (d, *J* = 8.3 Hz, 1H, H-4, ind.), 7.77 (dd, *J* = 5.0, 3.4 Hz, 1H, H-9 isoind.), 7.66 (d, *J* = 8.3 Hz, 2H, H-3 and H-5, tosyl), 7.55 (dd, *J* = 6.4, 3.0 Hz, 2H, H-2 and H-6, 9b-phenyl), 7.48 (dd, *J* = 5.5, 3.1 Hz, 2H, H-7 and H-8, isoind.), 7.44 (d, *J* = 8.4 Hz, 1H, H-7 ind.), 7.38 (m, 3H, H-3, H-4 and H-5, 9b-pheny), 7.33 (t, *J* = 7.2 Hz, 1H, H-6 ind.), 7.29 (t, *J* = 7.2 Hz, 1H, H-5 ind.), 7.17 (d, *J* = 8.2 Hz, 2H, H-2 and H-6, tosyl), 4.63–4.54 (m, 1H, H-3), 4.15 (dd, *J* = 8.7, 7.2 Hz, 1H, H-2), 3.90 (dd, *J* = 8.7, 7.2 Hz, 1H, H-2), 3.07 (dd, *J* = 14.1, 4.9 Hz, 1H, CH_2_-ind.), 2.69 (dd, *J* = 14.1, 10.0 Hz, 1H, CH_2_-ind.), 2.32 (s, 3H CH_3_). ^13^C NMR (101 MHz, CDCl_3_) δ_C_ 174.7 (C=O), 147.3 (C-9a isoind.), 145.4 (C-4 tosyl), 138.5 (C-1, 9b-phenyl), 137.6 (C-7a ind.), 135.2 (C-1 tosyl), 133.6 (C-8 isoind.), 131.0 (C-5a isoind.), 130.3 (C-7 isoind.), 129.8 (C-2 and C-6, tosyl), 129.7 (C-3a, ind.), 128.92 (C-3 and C-5, 9b-phenyl), 128.89 (C-4, 9b-phenyl), 127.1 (C-3 and C-5, tosyl), 125.9 (C-2 and C-6, 9b-pheny), 125.4 (C-6 ind.), 124.5 (C-9 isoind.), 123.7 (C-6 isoind.), 121.6 (C-Br), 118.9 (C-7 ind.), 115.7 (C-4, ind.), 110.3 (C-3 ind.), 101.1 (C-9b), 75.1 (C-2), 54.9 (C-3), 29.8 (CH_2_-ind.), 21.8 (CH_3_). MS-ESI(+)C_32_H_25_BrN_2_O_4_S [M+H]^+^. Found *m*/*z* 613. Retention time: 5.6 min.

**(3*R*, 9b*S*)-3-((2-bromo-1-propyl-1*H*-indol-3-yl)methyl)-9b-phenyl-2,3-dihydrooxazolo[2,3-*a*]isoindol-5(9b*H*)-one, 13d′.** Following the general procedure: (3*R*, 9b*S*)-3-((2-bromo-1*H*-indol-3-yl)methyl)-9b-phenyl-2,3-dihydrooxazolo[2,3-*a*]isoindol-5(9b*H*)-one (**13a′**) (150.0 mg, 326.55 µmol, 1 eq.), 2 mL of anhydrous DMF, sodium hydride (15.7 mg, 653.11 µmol 2 eq.) and propyl bromide (32.73 µL, 359.21 µmol, 1.1 eq.). Reaction time: 45 min. Eluent for flash chromatography: 3:7, ethyl acetate/*n*-hexane. Recrystallization in *n*-hexane/DCM. Product **13d′** was obtained as pale yellow solid (146.1 mg, 89%). [α]^20^_D_ = −75.5° (c = 0.39, CH_2_Cl_2_). The ^1^H NMR spectrum was found identical to the one of compound **13d**. MS-ESI(+) C_28_H_25_BrN_2_O_2_ [M+H]^+^. Found *m*/*z* 501. Retention time: 10.7 min.

### 3.7. Determination of Compounds Antiproliferative Activity via Sulforhodamine B SRB Assay

Tumor cells were cultured in RPMI-1640 with UltraGlutamine (Lonza, VWR), supplemented with 10% fetal bovine serum (FBS; Gibco, Alfagene) and maintained in a humidified incubator at 37 °C with 5% CO_2_. Cell lines were seeded in 96-well plates at the cells/well density of 5.0 × 10^3^. Cells were thereafter treated with serial dilutions (3.1–50.0 μM) of tryptophanol-derived isoindolinones for 48 h, and its effect on cell proliferation was analysed by SRB assay with the determination of GI_50_ (concentration that causes 50% of growth inhibition) values. The solvent (DMSO 0.25%) and nutlin-3a (GI_50_ = 3.2 ± 1.0 μM in HCT116 p53^+/+^ and GI_50_ = 24.0 ± 0.7 μM in HCT116 p53^−/−^) were included as controls.

### 3.8. Expression and Purification of Wild-Type p53 Core Domain

Expression of wt p53 core domain. The wt p53DBD was expressed in *E. coli* BL21 (DE3)-[pLys-S] in fusion with an N-terminal 21-residue tag containing the hexa-histidyl peptide (His_6_) and the thrombin recognition sequence, using the pET15b-based expression construct [48].

For protein expression 10 mL of an overnight (ON) culture was used to inoculate 1 L of LB medium supplemented with 1 mL of ampicillin 100 mg/mL and 1mL of zinc sulphate 100 mM. Culture medium was incubated at 37 °C, 140 rpm till an OD_600nm_ of ~0.5. Protein expression was then induced by adding 0.5 mL of isopropyl β-D-1-thiogalactopyranoside (IPTG) 1M (C_final_ = 0.5 mM) and bacterial growth at 27 °C, 120 rpm ON. *E. coli* cells were pelleted by centrifugation for 10 min, 4000 rpm, at 4 °C. The cellular pellet was resuspended in 10 mL of Lysis buffer (Sodium phosphate 50 mM; NaCl 300 mM; glycerol 10%, pH 7.4) supplemented with 1mg/mL Lysozyme (Biochemica Applichem Panreac), 1 mM of phenylmethanesulfonyl fluoride (PMSF) (Merck), 5 µg of DNAse I (Panreac applichem) and 0.8% of Triton X-100 (Merck). After a first step of chemical lysis (30 min incubation, at 4 °C), cells were further disrupted by four cycles of sonication, at 4 °C, during 60 s at 50% duty free cycle on a Vibra Cell (Media Cibernetics). The lysate was subsequently cleared by centrifugation at 10,000 rpm, 40 min, at 4 °C and the supernatant containing the soluble proteins were collected.

Purification of human wt p53DBD and His_6_-tag removal. Immobilized Metal Affinity Chromatography (IMAC) was performed to purify the wt p53DBD using a nickel-nitrilotriacetic acid (Ni-NTA) agarose resin (QIAGEN). Briefly, 1 mL of Ni^2+^ resin was pre-equilibrated using MilliQ water (3 × 1 mL) and Lysis buffer containing 10 mM imidazole (3 × 1 mL). The soluble fraction of cellular lysate was supplemented with 6.8 µL of β-mercaptoethanol and 33.2 µL of imidazole 3 M to obtain the final concentrations of 10 µM and 10 mM, respectively. The lysate was added to the pre-equilibrated resin and protein binding was performed by incubation on an orbital shaker for one hour at 4 °C. The suspension was added to a 9 cm polypropylene conical column (Poly-Prep columns; BioRad) and protein purification was performed by gravity flow with an imidazole gradient consisting of three washing steps (2 × 5 mL of Lysis buffer, 20 mM imidazole; 2 × 5 mL of Lysis buffer, 50 mM imidazole; and 1 × 2.5 mL Lysis buffer, 75 mM imidazole) and an elution step with 5 × 500 µL of Lysis buffer, 250 mM imidazole. Protein eluted fractions were assessed by SDS-PAGE and Coomassie staining. Size exclusion chromatography was performed on the pooled protein eluted fractions, using an AKTA prime plus system (GE Healthcare) equipped with a UV detector (280 nm), a HiLoad 16/60, Superdex 200 prep grade column (GE Healthcare) and SEC buffer (25 mM potassium phosphate buffer, 150 mM NaCl, pH 7.5). The fractions corresponding to the wt p53DBD monomers were collected and treated with thrombin (8 units of thrombin/mg wt p53DBD). The thrombin cleavage was conducted for 18 h at rt at pH 7.4 in the assay buffer recommended by the supplier. Before and after digestion protein concentration was determined through Bradford method.

### 3.9. Differential Scanning Fluorimetry (DSF)

Thermostability of wt p53DBD core domain assessed by differential scanning fluorimetry (DSF). An amount of 5 μg of thrombin-cleaved wt p53DBD was incubated with testing compounds (2.5 mM) in 25 mM potassium phosphate buffer and 150 mM NaCl, pH 7.5 in a final volume of 50 µL containing 2 µL of 1 mM DTT or 1 mM TCEP to promote p53 core domain redox control, and Sypro Orange at 2.5x final concentration. The fluorescence was assessed on a C1000 Touch thermal cycler equipped with a CFX96 optical reaction module (BioRad) and the FRET channel (λexc 450–490 nm and λem 560–580 nm). Temperature was ramped from 20 to 90 °C with a rate of 0.2 °C/12 sec. The *T*_m_ values were calculated using Origin 8 Software. Control assays included: (1) wt p53DBD in SEC buffer, in the absence of the compounds; (2) wt p53DBD in SEC buffer and in presence of DMSO 5%; (3) wt p53DBD in SEC buffer an in the presence of 1mM DTT or TCEP; (4) blank assays (testing compounds in absence of wt p53DBD and in the absence and presence of 1mM DTT and TCEP). In all assays 5% of DMSO was included. The compound MQ was used as a positive control according to the assay conditions described in the literature [45].

## 4. Conclusions

In this work, the pharmacokinetic profile of hit compounds SLMP53-1 (**6**) and SLMP53-2 (**7**) was studied. Moreover, a biomimetic synthesis of the SLMP53-1 phase I metabolites, identified in HLM incubations, was performed using a nonheme-containing iron(II) complex for the selective oxidation of the indole moiety. Whereas this class of catalysts was reported to promote oxidation of a wide range of chemically diverse substrates [30], this constitutes the first report on their efficient oxidation of indole rings and on their ability to mimic CYP-promoted indole ring metabolic degradation. Furthermore, based on the metabolic liabilities identified in SLMP53-1 (**6**), 16 enantiopure tryptophanol-derived isoindolinones were prepared. The antiproliferative activity of these compounds was evaluated in HCT116 cell lines with and without p53. Compared with hit compound SLMP53-2 (**7**), derivative **13d** was 2-fold more active in HCT116 p53^+/+^ cells, while derivative **13k** was 2-fold selective for the p53 pathway. PK experiments showed that insertion of a bromine at the C2 indole minimizes the formation of catabolic products of C-2 oxidation and indole ring opening, which were shown to be associated with decreased activity. However, a metabolic switch towards the formation of potential phenolic metabolites at the indole moiety was observed. While the toxicology/activity impacts of these phenolic metabolites should be evaluated before more improvements in this scaffold, the C2-bromine per se did not constitute a metabolic lability, being inert towards the insertion of bionucleophiles. Whereas potential bioactivation pathways were identified following Phase I metabolism of these halogen-enriched compounds, **13k** was suggested to be less prone to this type of biotransformation. Finally, DSF experiments showed that lead compound **13d** interacts directly with wt-p53DBD promoting protein p53 thermostability, increasing wt-p53DBD melting temperature with ΔTm 10.4 °C. Together, this study highlights the potential of the tryptophanol-derived isoindolinone scaffold as p53 activators in CRC.

## Figures and Tables

**Table 1:** Antiproliferative activity of Phase I metabolites of SLMP53-1 (**6**) in human colon adenocarcinoma HCT116 cells expressing p53 (HCT116 p53^+/+^) and HCT116 cells with p53 expression knocked out (HCT116 p53^−/−^). DMSO was used as control. Assays were performed upon 48 h treatment. Results are presented as Mean ± SEM of 5 independent experiments.

Metabolite	HCT116 p53^+/+^ GI_50_ (µM)	HCT116 p53^−/−^ GI_50_ (µM)
SLMP53-1_M5	48.8 ± 2.3	44.0 ± 1.8
SLMP53-1_M3a	>50	>50
SLMP53-1_M3b	>50	>50
SLMP53-1 [24]	15.6 ± 1.6	34.0 ± 3.5

**Table 2:** Tryptophanol-derived isoindolinones **13a** and **13c-o** screened in HCT116 human colorectal carcinoma cells expressing the p53 (HCT116 p53^+/+^) and with p53 knocked out (HCT116 p53^−/−^). Growth inhibitory effect (GI_50_) was determined after 48 h treatment; growth obtained with control (DMSO) was set as 100%. Data are mean ± SEM of 3–5 independent experiments. N.A.—not applicable. S.I.—selectivity index.

Compound	HCT116 p53^+/+^ GI_50_ (µM)	HCT116 p53^−/−^ GI_50_ (µM)	S.I.
13a	14.6 ± 0.4	10.5 ± 2.6	0.7
13c	25.6 ± 1.4	10.8 ± 2.0	0.4
13d	4.00 ± 0.50	7.45 ± 0.95	1.9
13g	6.95 ± 0.6	6.7 ± 1.6	1.0
13h	5.8 ± 0.3	5.8 ± 0.4	1.0
13i	23.3 ± 0.9	22.2 ± 1.2	1.0
13j	13.0 ± 1.01	16.0 ± 2.00	1.2
13k	8.09 ± 2.01	30.5 ± 2.10	3.8
13l	23.15 ± 1.15	>50	N.A.
13m	13.05 ± 0.05	22.5 ± 2.50	1.7
13n	12.50 ± 0.50	13.50 ± 0.50	1.1
13o	34.00 ± 4.00	20.35 ± 1.15	0.6
SLMP53-2	8.4 ± 1.1	17.7 ± 2.3	2.1

**Table 3:** Half-life and intrinsic clearance values obtained for **13d**, **13k** and **7** determined upon in vitro incubation in HLM.

Compound	Half-Life (min)	Intrinsic Clearance (mL min^−1^ Kg^−1^)
13d	92	5.5
13k	147	3.4
SLMP53-2 (7)	147	2.8

**Table 4:** Melting temperatures (*T*_m_) of the wt p53DBD in the absence (control) and presence of tested compounds and using different reducing agents (DTT and TCEP).

	*T*_m_ (°C)	
	DTT (1 mM)	TCEP (1 mM)
Control (5% DMSO)	40.11 ± 0.28	40.99 ± 0.07
MQ	42.22 ± 0.30	43.27 ± 1.15
13d	40.51 ± 0.09	51.38 ± 0.38

Note: compounds were tested in 5% DMSO; data represent the mean ± standard deviation (SD) from three independent assays.

## Data Availability

Not applicable.

## Supplementary Materials

The following supporting information can be downloaded at: https://www.mdpi.com/article/10.3390/ph16020146/s1, Figure S1: Stability of compound SLMP53-1 (6) and SLMP53-2 (7) in human plasma at 37 °C; Figure S2: (a) Selected HRMS-ESI(+) spectrum of SLMP53-1 (6) HLM incubations; (b) Compound SLMP53-1 (6) depletion plot; Figure S3: (a) Selected full scan ion chromatogram, obtained by LC-ESI-(+)-MS, for SLMP53-2 (7) incubation in human liver microsomes; (b) SLMP53-2 (7) depletion plot; Figure S4: (a) LC-ESI(+)-HRMS total ion chromatogram obtained for SLMP53-1 (6) HLM incubations, and extracted ion chromatograms at: (b) *m*/*z* 319.1441, corresponding to SLMP53-1 (6), in pink; (c) *m*/*z* 335.1390, corresponding to SLMP53-1_M1, SLMP53-1_M2 and SLMP53-1_M3, in blue; (d) *m*/*z* 351.1339, corresponding to SLMP53-1_M4 and SLMP53-1_M5 in orange; (e) *m*/*z* 323.1390, corresponding to SLMP53-1_M6, in green; Figure S5: (a) LC-ESI(+)-HRMS/MS spectra obtained for SLMP53-1_M1, SLMP53-1_M2 and SLMP53-1_M3; and (b) Proposed fragmentation pattern; Figure S6: (a) LC-ESI(+)-HRMS/MS spectra obtained for SLMP53-1_M4 and SLMP53-1_M5; and (b) proposed fragmentation patterns; Figure S7: (a) LC-ESI(+)-HRMS/MS spectrum obtained for SLMP53-1_M6; and (b) Proposed fragmentation pattern; Figure S8: LC-ESI(+)-HRMS/MS spectrum obtained for SLMP53-2_M1 and proposed fragmentation pattern; Figure S9: LC-ESI(+)-HRMS/MS spectrum obtained for SLMP53-2_M2 and proposed fragmentation pattern; Figure S10: LC-ESI(+)-HRMS/MS spectrum obtained for SLMP53-2_M3 and proposed fragmentation pattern; Figure S11: LC-ESI(+)-HRMS/MS spectrum obtained for SLMP53-2_M4 and proposed fragmentation pattern; Figure S12: LC-ESI(+)-HRMS/MS spectrum obtained for SLMP53-2_M5 and proposed fragmentation pattern; Figure S13: LC-ESI(+)-HRMS/MS spectrum obtained for SLMP53-2_M6 and proposed fragmentation pattern; Figure S14: Plots of the relative abundance over incubation time for SLMP53-1 (6) and its Phase I metabolites; Figure S15: Plots of the relative abundance over incubation time for SLMP53-2 (7) and its Phase I metabolites; Figure S16: LC-ESI(-)-HRMS/MS spectrum obtained for SLMP53-1_Glu1 and proposed fragmentation mechanism; Figure S17: LC-ESI(-)-HRMS/MS spectrum obtained for SLMP53-1_Glu2 and proposed fragmentation mechanism; Figure S18: LC-ESI(+)-HRMS extracted ion chromatogram at *m*/*z* 323.1390, corresponding to SLMP53-1_M5: (a) in the 24h biomimetic oxidation reaction; and (b) in HLM incubations; Figure S19: LC-ESI(+)-HRMS/MS spectrum, obtained for SLMP53-1_M5: (a) in the 24h biomimetic oxidation reaction; and (b) in HLM incubations; Figure S20: LC-ESI(+)-HRMS extracted ion chromatogram at *m*/*z* 351.1339, corresponding to SLMP53-1_M6: (a) in the 24h biomimetic oxidation reaction; and (b) in HLM incubations; Figure S21: LC-ESI(+)-HRMS/MS spectra, obtained for SLMP53-1_M6: (a) in the biomimetic oxidation; and (b) in the HLM incubations; Figure S22: LC-ESI(+)-HRMS extracted ion chromatogram at *m*/*z* 335.1390, corresponding to: (a) SLMP53-1_M3a and (b) SLMP53-1_M3b, obtained in the 5h biomimetic oxidation of SLMP53-1 (6); and (c) SLMP53-1_M1, SLMP53-1_M2 and SLMP53-1_M3 detected in HLM incubations. Products of biomimetic oxidation SLMP53-1_M3a and SLMP53-1_M3b show compatible retention times with one major Phase I metabolite SLMP53-1_M3 observed in the HLM incubations; Figure S23: LC-ESI(+)-HRMS/MS spectra, obtained for: (a) SLMP53-1_M3a; and b) SLMP53-1_M3b in the biomimetic oxidation reaction; and (c) SLMP53-1_M3 detected in the HLM incubations; Figure S24: LC-ESI(+)-HRMS/MS extracted ion chromatogram at *m*/*z* 351.1339, corresponding to (a) SLMP53-1_M4 and, SLMP53-1_M5 detected in HLM incubations; and (b) SLMP53-1_M4 detected in the biomimetic oxidation of hit compound SLMP53-1 (6); Figure S25: LC-ESI(+)-HRMS/MS spectra obtained for SLMP53-1_M4 in: (a) the biomimetic oxidation reaction; and (b) the HLM incubations; Figure S26: ^1^H NMR spectrum, obtained in CDCl_3_, of SLMP53-1_M5; (a) Expansion between 8.8 ppm and 7.1 ppm; and (b) Expansion of 1H NMR spectrum between 4.8 ppm and 3.4 ppm; Figure S27: ^13^C NMR spectrum, obtained in CDCl_3_, of SLMP53-1_M5; (a) Expansion of ^13^C NMR spectrum between 138 ppm and 116 ppm; Figure S28: Expansion between 4.6 and 2.0 ppm, obtained in CDCl_3_, of the diastereomers mixture SLMP53-1_M3a and SLMP53-1_M3b; Figure S29: Expansion between 8.1 and 6.8 ppm of ^1^H NMR spectrum, obtained in CDCl_3_, for the diastereomers mixture SLMP53-1_M3a and SLMP53-1_M3b; Figure S30: ^13^C NMR spectrum, obtained in CDCl_3_ for the diastereomers mixture SLMP53-1_M3a and SLMP53-1_M3b; (a) ^13^C NMR spectrum expansion between 55 ppm and 30 ppm; (b) ^13^C NMR spectrum expansion between 130 ppm and 122 ppm; c) ^13^C NMR spectrum expansion between 135 ppm and 130 ppm; Figure S31: Expansion between 8.5 and 1.5 ppm of ^1^H NMR spectrum obtained in CDCl_3_ for SLMP53-1_M4; (a) Expansion between 4.70 and 4.40 ppm; (b) Expansion between 8.2 and 7.0 ppm; Figure S32: ^13^C NMR spectrum obtained in CDCl_3_ for SLMP53-1_M4; (a) Expansion from 79.0 to 74.0 ppm; (b) Expansion from 125.5 ppm and 122.0 ppm; (c) Expansion from 134.0 ppm to 130.0 ppm; Figure S33: (a) LC-ESI(+)-HRMS/MS spectrum obtained for SLMP53-1-GS1; and (b) Proposed fragmentation pattern; Figure S34: LC-ESI(+)-HRMS/MS spectra obtained for SLMP53-1-GS2 and proposed fragmentation pattern; Figure S35: ^1^H NMR spectrum of compound 13d, obtained in CDCl_3_; Figure S36: ^13^C APT NMR spectrum of compound 13d, obtained in CDCl_3_; Figure S37: ^1^H NMR spectrum of compound 13k, obtained in CDCl_3_; Figure S38: ^13^C APT NMR spectrum of compound 13k, obtained in in CDCl_3_; Figure S39: HPLC-DAD spectrum of compound 13d at concentration 10^-3^ M; Figure S40: HPLC spectrum of compound 13d at the concentration 10^-3^ M; Figure S41: LC-ESI(+)-HRMS/MS spectra obtained for 13d and proposed fragmentation pattern; Figure S42: LC-ESI(+)-HRMS/MS spectra obtained for 13k and proposed fragmentation pattern; Figure S43: Extracted ion HRMS-ESI(+) chromatogram at *m*/*z* 517.1121, obtained for 13d HLM incubations and LC-ESI(+)-HRMS/MS spectra obtained for (a) 13d_M1, (b) 13b_M2, and (c) 13d_M3. Proposed fragmentation pattern; Figure S44: Extracted ion HRMS-ESI(+) chromatogram at *m*/*z* 565.1121 corresponding to 13k_M1-M3 in HLM incubations. HRMS/MS spectrum and proposed fragmentation pattern; Figure S45: LC-ESI(+)-HRMS/MS spectra obtained for 13d_M4 and 13k_M4, and proposed fragmentation pattern; Figure S46: LC-ESI(+)-HRMS/MS spectra obtained for 13d_M5 and 13k_M5, and proposed fragmentation pattern; Figure S47: LC-ESI(+)-HRMS/MS spectra obtained for 13d_M6 and proposed fragmentation pattern; Figure S48: LC-ESI(+)-HRMS/MS spectra obtained for 13d_M7 and proposed fragmentation pattern; Figure S49: Plots of the relative abundances over incubation time for 13d and its Phase I metabolites; Figure S50: Plots of the relative abundancies over incubation time for 13k and its Phase I metabolites; Figure S51: Extracted ion HRMS-ESI(+) chromatogram at *m*/*z* 822.1802. LC-ESI(+)-HRMS/MS spectra obtained for 13d-GS1 and proposed fragmentation pattern; Figure S52: (a) Extracted ion HRMS-ESI(+) chromatograms at *m*/*z* 764.1384 of 13d and 13k HLM incubations; (b) LC-ESI(+)-HRMS/MS spectra obtained for 13k-GS1, 13k-GS2, 13d-GS2 and 13d-GS3; and (c) proposed fragmentation mechanisms; Figure S53: Extracted ion HRMS-ESI(+) chromatogram at *m*/*z* 780.1378. LC-ESI(+)-HRMS/MS spectra obtained for 13d-GS4 and proposed fragmentation mechanisms; Figure S54: Thermal denaturation curves obtained by DSF assay of the wt p53DBD in the absence and presence of compounds. (Black) 1 mM TCEP; (green) 1 mM TCEP and 2 mM of MQ; (red) 1 mM TCEP and 2.5 mM of compound 13d. The DSF assays were performed with the fluorophore SYPRO Orange. The presence of compound 13d led to an increase in the wt p53DBD Tm of 10.35 °C, while in MQ afforded an increase of 2.11 °C.
