# Supplementary material for: Metabolism-Guided Optimization of Tryptophanol-Derived Isoindolinone p53 Activators
Source: Pharmaceuticals (Basel). 2023 Jan 19;16(2):146. doi: 10.3390/ph16020146 (PMC9967700; doi:10.3390/ph16020146)
Supplement: Supplementary file 1 [file pharmaceuticals-16-00146-s001.zip › pharmaceuticals-2136622-supplementary.pdf]

# Supporting Information

**Metabolism-guided optimization of tryptophanol-derived isoindolinone p53 activators**

Valentina Barcherini [1], Joana B. Loureiro [2], Ana Sena [3], Catarina Madeira [1], Paula Leandro [1], Lucília Saraiva [2], Alexandra M. M. Antunes [3], and Maria M. M. Santos [1]

[1] Research Institute for Medicines (iMed.ULisboa), Faculty of Pharmacy, Universidade de Lisboa, Lisboa, Portugal

[2] LAQV/REQUIMTE, Laboratório de Microbiologia, Departamento de Ciências Biológicas, Faculdade de Farmácia, Universidade do Porto, Porto, Portugal

[3] Centro de Química Estrutural (CQE), Institute of Molecular Sciences, Departamento de Engenharia Química, Instituto Superior Técnico (IST), Universidade de Lisboa, Lisboa, Portugal

**Table of contents:**

| | |
|---|---|
| Materials and Methods General Information | 1 |
| Depletion plots obtained for plasma and HLM incubation of SLMP53-1 (**6**) and SLMP53-2 (**7**) | 3 |
| LC-HRMS/MS data of SLMP53-1 Phase I metabolites | 5 |
| LC-HRMS/MS data of SLMP53-2 Phase I metabolites | 9 |
| Plots of relative abundance over HLM incubation time for SLMP53-1, SLMP53-2 and their Phase I metabolites | 12 |
| LC-HRMS/MS data of SLMP53-1 Phase II metabolites | 13 |
| LC-HRMS/MS data for the biomimetic reaction of SLMP53-1 | 14 |
| $^{1}$H NMR and $^{13}$C NMR spectra of SLMP53-1 Phase I metabolites | 17 |
| LC-HRMS/MS data of SLMP53-1 glutathione adducts | 24 |
| $^{1}$H NMR and $^{13}$C NMR spectra of **13d** and **13k** | 25 |
| HPLC data of compounds **13d** and **13k** | 28 |
| LC-HRMS/MS data of **13d** and **13k** and their Phase I metabolites | 30 |
| Plots of relative abundance over HLM incubation time of **13d** and **13k** and their Phase I metabolites | 36 |
| LC-HRMS/MS data of **13d** and **13k** glutathione adducts | 37 |
| Thermostability of the wt p53DBD in the presence of studied compounds as monitored by differential scanning fluorimetry (DSF) | 40 |

# Materials and Methods General Information

Thin layer chromatography (TLC) was carried out on normal phase Merck silica gel 60 F254 aluminium sheets and visualized by UV light ($\lambda_{max}$=254/360 nm). TLC plates were saturated with ninhydrin or potassium permanganate solutions when necessary. Flash column chromatography was performed on normal phase Merck Silica Gel (200-400 mesh). Preparative TLC was performed on Merck Silica Gel 60 GF254 over glass plates with 0.5 and 1 mm thickness.

The separation of Phase I metabolites SLMP53-1_M3a and SLMP53-1_M3a was performed on a LaPrep VWR system constituted by a P110 pump and UV detector P314 set at λ = 250 nm. A Luna® 100 C18 [5 µm, 100 Å, 250×4.6 mm], with a constant flow rate of 5.0 mLmin$^{-1}$ and an isocratic solvents system of ACN:$H_2O$ 55:45 for 80 min.

All compounds showed purity ≥ 95% when eluted in a LaChrom HPLC constituted of a Merck Hitachi pump L-7100, Merck Hitachi autosampler L-7250, and a Merck Hitachi UV detector L-7400. Analyses were performed with a LiChrospher®100 RP-C8 (5 µm, 100 Å) LiChroCART® 250-4 column at room temperature, using a mobile phase solution constituted of 65% acetonitrile and 35% Milli-Q water with a constant flow of 1.0 mL min$^{-1}$. Peaks were detected at λ = 254 nm.

MS experiments were performed on Micromass® Quattro Micro triple quadrupole (Waters®, Ireland) with an electrospray in positive ion mode (ESI+), ion source at 120 °C, capillary voltage of 3.0 kV and source voltage of 30 V, at the Liquid Chromatography and Mass Spectrometry Laboratory, Faculty of Pharmacy, University of Lisbon.

$^{1}$H NMR spectra were recorded on a Bruker Biospin Fourier 300, a Avance II 400, or a Avance III 500 Bruker NMR spectrometers, operating at 300, 400 and 500 MHz, respectively. $^{13}$C NMR spectra were recorded on the same instruments, operating at 75 MHz, 101, 126 MHz, respectively. $^{1}$H and $^{13}$C NMR chemical shifts are reported as $\delta_H$ and $\delta_C$, respectively, expressed in parts per million (ppm). The spectra were referenced to the deuterated solvent peak and coupling constants (*J*) are reported in Hertz (Hz). Resonance and structural assignments were based on the analysis of coupling patterns, including the $^{13}$C-$^{1}$H coupling profiles obtained in bidimensional heteronuclear multiple quantum coherence (HMQC) or heteronuclear single quantum coherence (HSQC) and heteronuclear multiple bond correlation (HMBC) experiments, performed with Bruker standard pulse programs. Whenever necessary, APT and $^{1}$H-$^{1}$H bidimensional correlation spectroscopy (COSY) experiments were also performed to assist the structural assignments. Multiplicities in $^{1}$H NMR spectra are indicated by s (singlet), d (doublet), dd (doublet of doublets), ddd (doublet of doublets of doublets), dt (doublet of triplets). t (triplet), tdd (triplet of doublets of doublets), q (quartet), m (multiplet) or br s (broad singlet). The number of protons (n) are given as nH. Multiplicity in $^{13}$C NMR spectra can be given as d (doublet), q (quartet) or septet in the case of compounds with C-F bonds.

Specific optical rotations (α) were measured using a P-200 high-accuracy digital polarimeter JASCO or a Perkin Elmer Model 241 polarimeter, with a sodium lamp at 589 nm. The concentration (c) is given in g/100 mL.

Melting points were determined using a Kofler camera Bock monoscope M or a Leica Galen III hot stage microscope.

HPLC-DAD analyses were conducted on an Ultimate 3000 Dionex system, consisting of an LPG-3400A quaternary gradient pump equipped with a diode array spectrophotometric detector (Dionex Co., Sunnyvale, CA) and a Rheodyne model 8125 injector (Rheodyne, Rohnert Park, CA). HPLC analyses were performed with a Luna C18 (2) column (250 mm x 4.6 mm; 5 µm; Phenomenex, Torrance, CA), at a flow of 1 mL/min. A 30-min linear gradient from 5 to 95% acetonitrile in 0.1% aqueous formic acid, followed by a 2-min linear gradient to 100% acetonitrile and an 8-min isocratic elution with acetonitrile was used in all instances. The UV absorbance was monitored at 268 nm.

LC-ESI-HRMS/MS analyses were performed in an ultra-high-performance liquid chromatography (Elute UHPLC, Bruker, Bremen, Germany) interfaced with a Bruker Impact II quadrupole time-of-flight (QTOF) mass spectrometer equipped with an electrospray ESI source (Bruker Daltoniks, Bremen, Germany). Chromatographic separation was carried out on a Luna C18 column (2.0 mm x 150 mm, 3.0

µm particle size; Phenomenex). The mobile phase consisted of water containing 0.1% formic acid (A) and acetonitrile (B), at a flow rate of 170 µL/min. The elution conditions were as follows: 5% B for 1.5 min; 5-100% B for 8.0 min; 100% B for 3.5 min; 100-5% B for 0.25 min; and to end 5% B for 1.75 min. Injection volume was 10 µL. The column and the autosampler were maintained at 40 °C and 8 °C, respectively. The high-resolution mass spectra were acquired in both positive and negative ion modes. The mass spectrometric parameters were set as follows: end plate offset 500 V; capillary voltage: 4.5 and 2.5 kV (positive and negative mode, respectively); nebulizer: 40 psi; dry gas: 8 L/min; heater temperature: 200 °C. Internal calibration was achieved with a sodium formate/acetate solution introduced to the ion source *via* a 20 µL loop at the beginning of each analysis using a six-port valve. Calibration was then performed using high-precision calibration mode (HPC). Acquisition was performed in full scan mode for the testing compounds and their metabolites in the *m/z* 50-1000 range in a data-dependent MS/MS mode with an isolation window of 0.5, acquisition rate of 3 Hz using a dynamic method with a fixed cycle of time of 3 s. Precursor ions were selected for auto MS/MS at an absolute threshold of 153, with the active exclusion mode set at three spectra and released after 1 min, but precursor ions with intensities in the range of 5x the previous intensities were reconsidered.

## Depletion plots obtained for plasma and HLM incubation of SLMP53-1 (6) and SLMP53-2 (7)

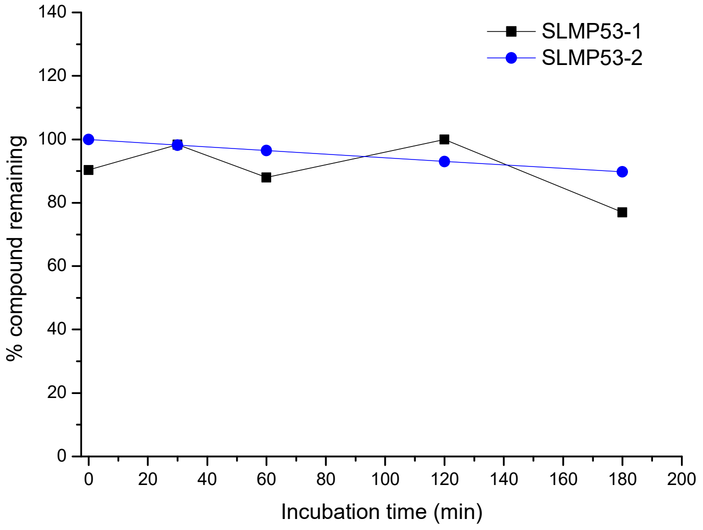


**Figure S1:** Stability of compounds SLMP53-1 (**6**) and SLMP53-2 (**7**) in human plasma at 37 °C.

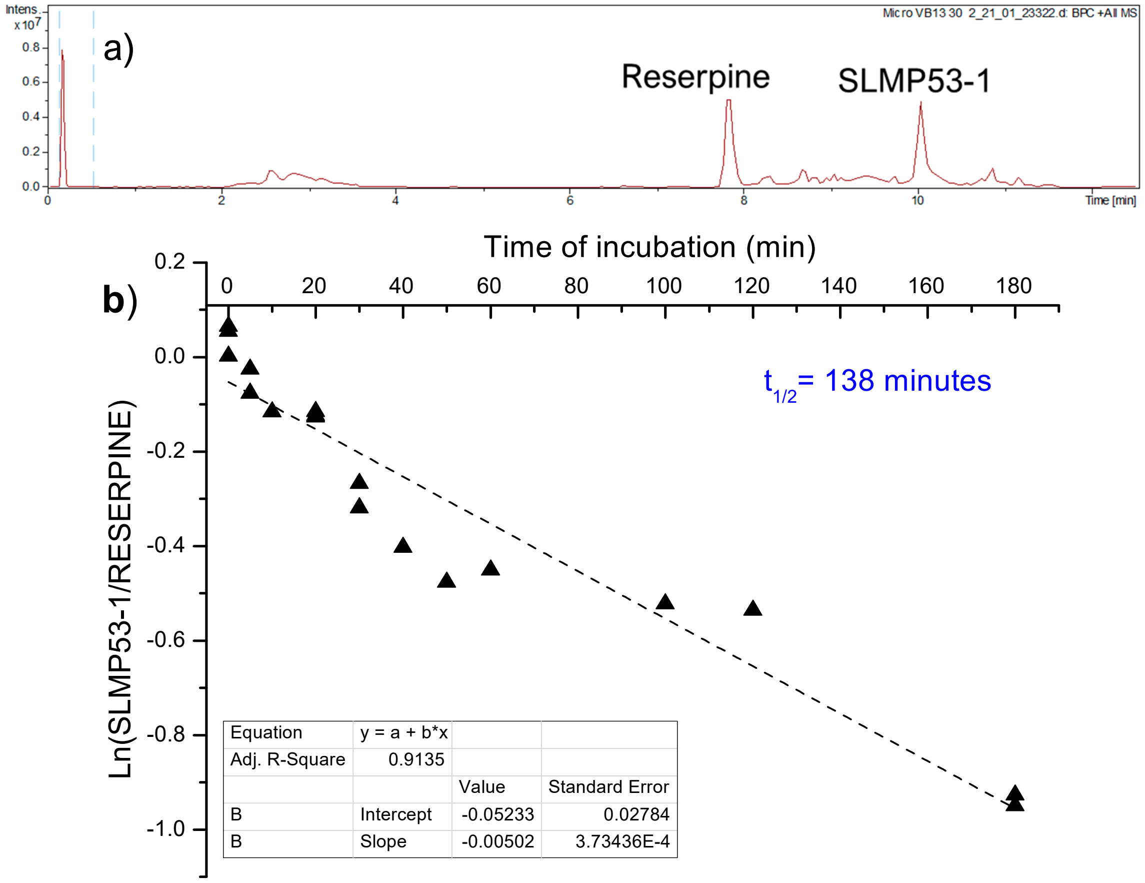


**Figure S2:** a) Selected HRMS-ESI-(+) spectrum of SLMP53-1 (**6**) HLM incubations; b) Compound SLMP53-1 (**6**) depletion plot.

a)

b)

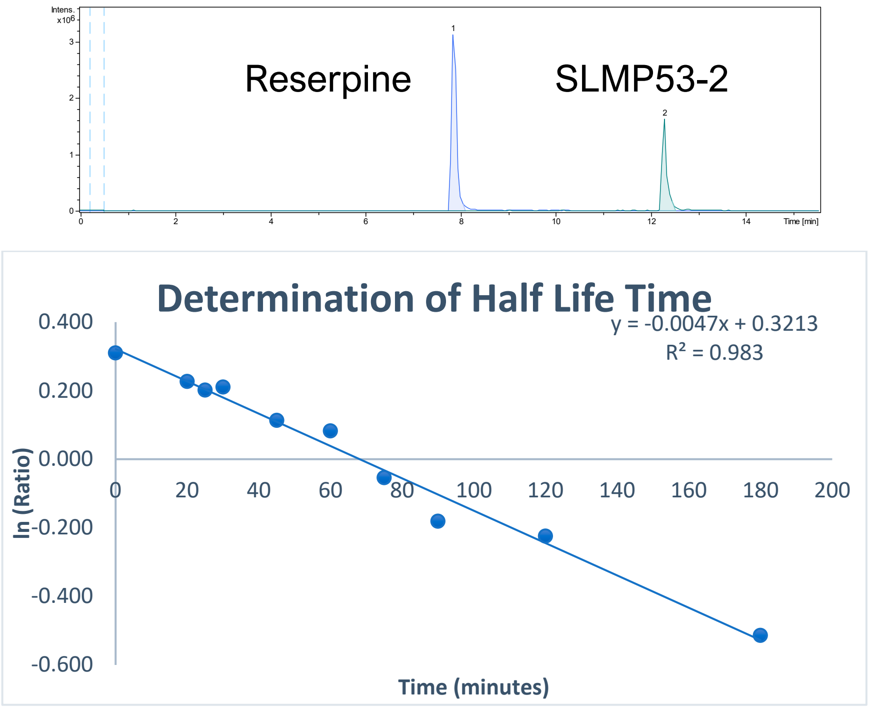


**Figure S3:** a) Selected full scan ion chromatogram, obtained by LC-ESI-(+)-MS, for SLMP53-2 (**7**) incubation in human liver microsomes; b) SLMP53-2 (**7**) depletion plot.

## LC-HRMS/MS data of SLMP53-1 Phase I metabolites

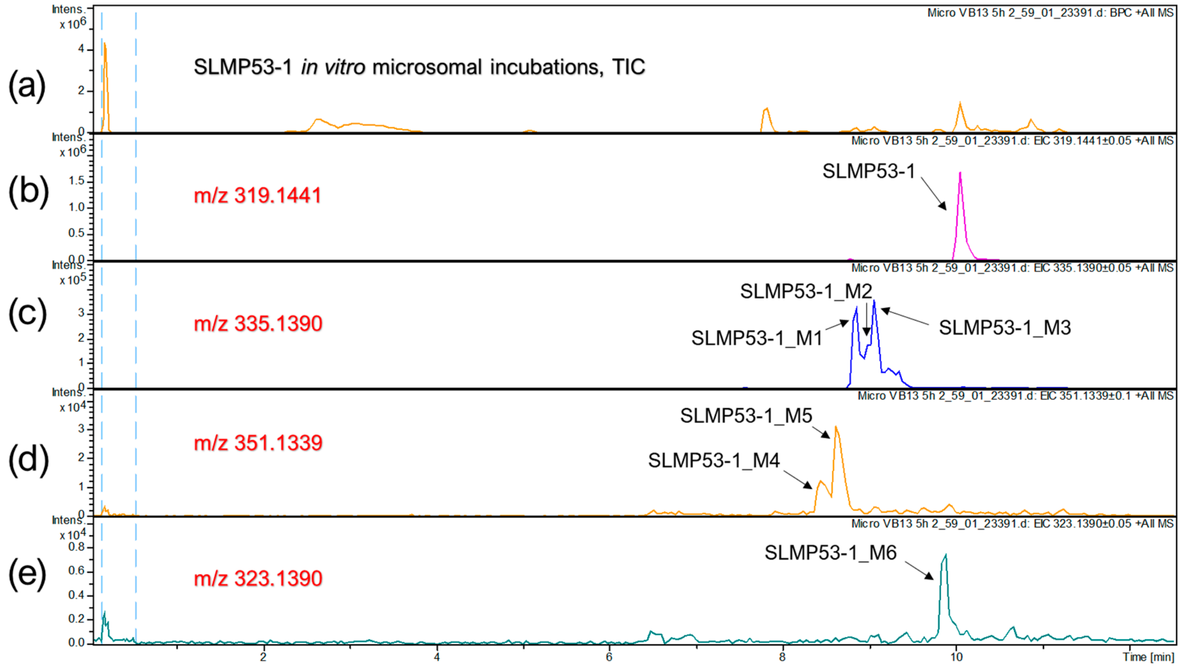


**Figure S4:** (a) LC-ESI(+)-HRMS total ion chromatogram obtained for SLMP53-1 (**6**) HLM incubations, and extracted ion chromatograms at: (b) *m/z* 319.1441, corresponding to SLMP53-1 (**6**), in pink; (c) *m/z* 335.1390, corresponding to **SLMP53-1_M1**, **SLMP53-1_M2** and **SLMP53-1_M3**, in blue; (d) *m/z* 351.1339, corresponding to **SLMP53-1_M4** and **SLMP53-1_M5** in orange; (e) *m/z* 323.1390, corresponding to **SLMP53-1_M6**, in green.

.

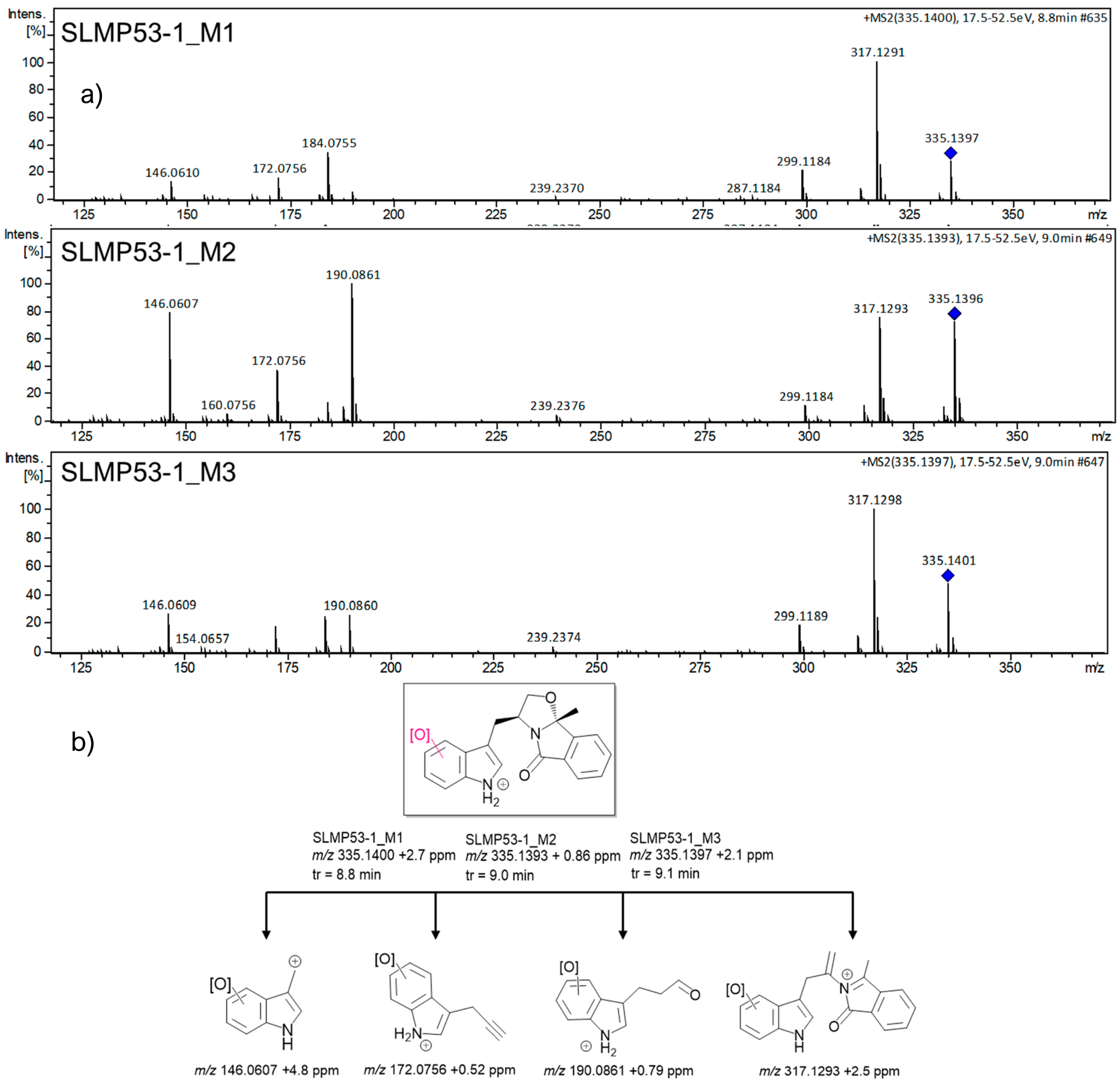


**Figure S5:** a) LC-ESI(+)-HRMS/MS spectra obtained for **SLMP53-1_M1**, **SLMP53-1_M2** and **SLMP53-1_M3**; and b) Proposed fragmentation pattern.

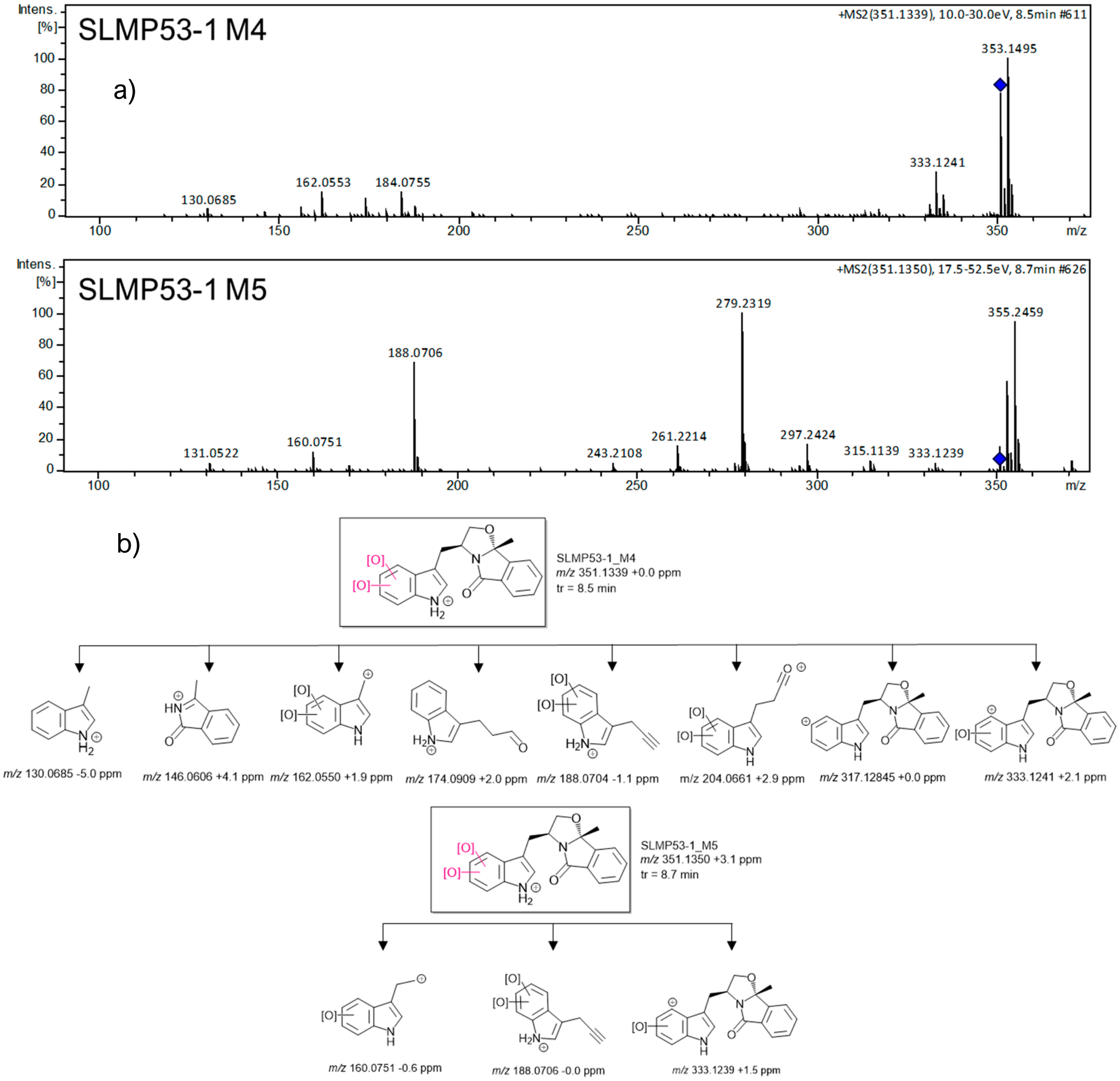


**Figure S6:** a) LC-ESI(+)-HRMS/MS spectra obtained for **SLMP53-1_M4** and **SLMP53-1_M5;** and b) proposed fragmentation patterns.

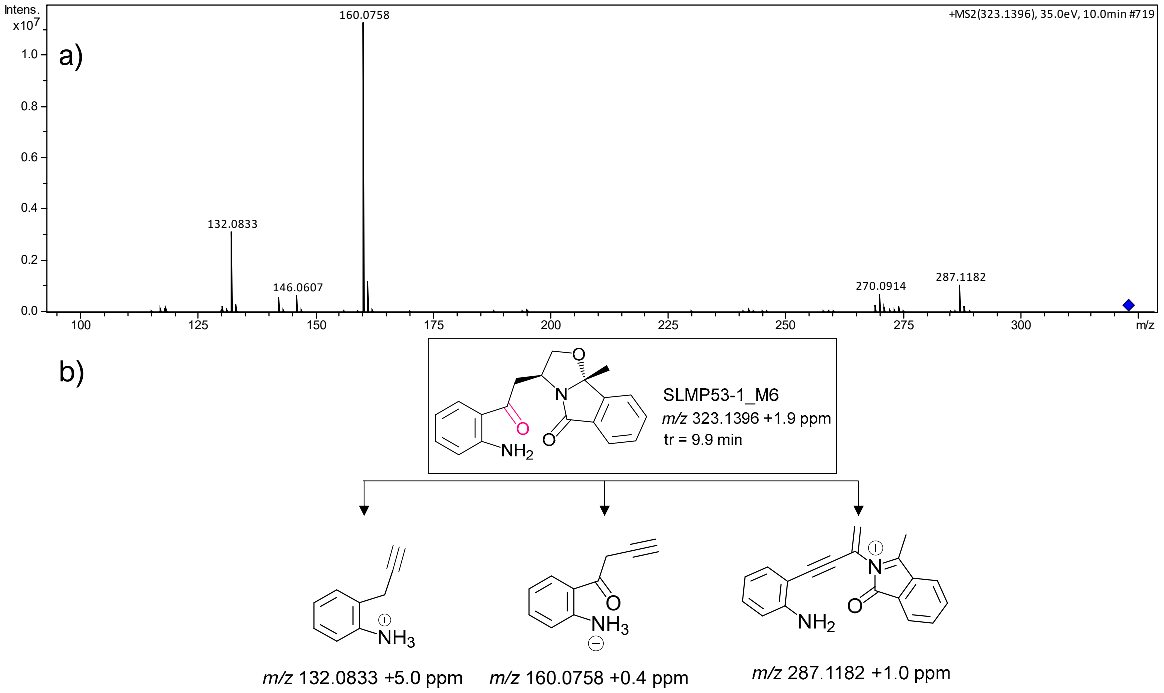


**Figure S7:** a) LC-ESI(+)-HRMS/MS spectrum obtained for **SLMP53-1_M6**; and b) Proposed fragmentation pattern.

# LC-HRMS/MS data of SLMP53-2 Phase I metabolites

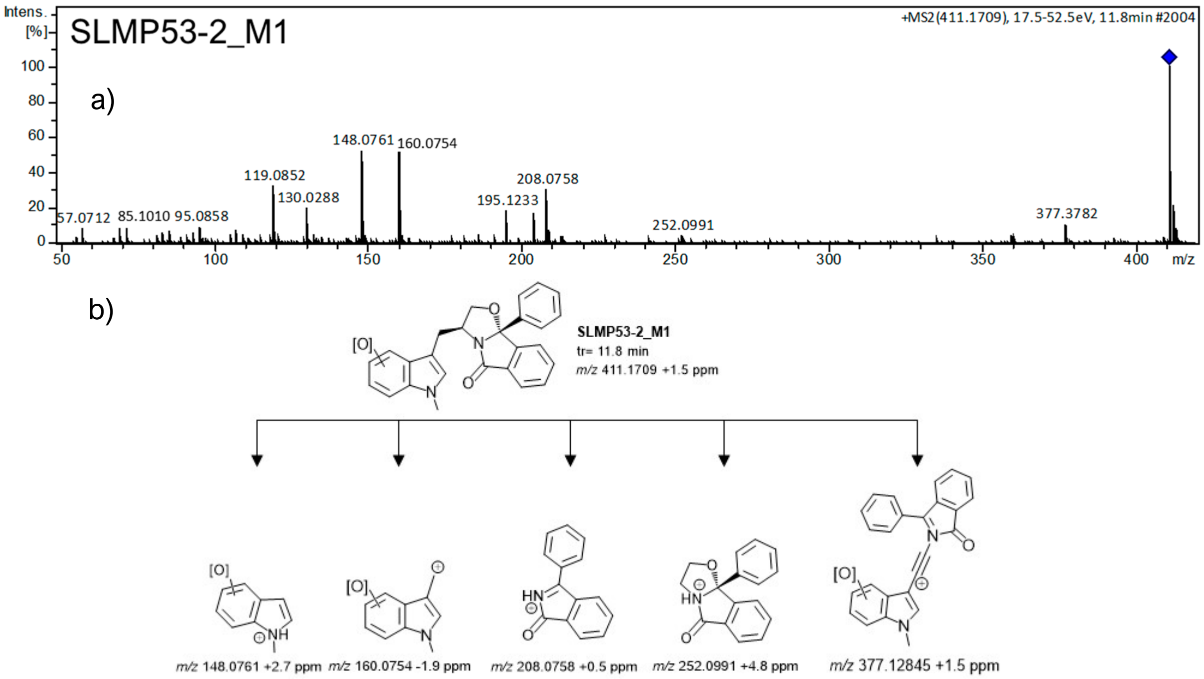


**Figure S8:** LC-ESI(+)-HRMS/MS spectrum obtained for **SLMP53-2_M1** and proposed fragmentation pattern.

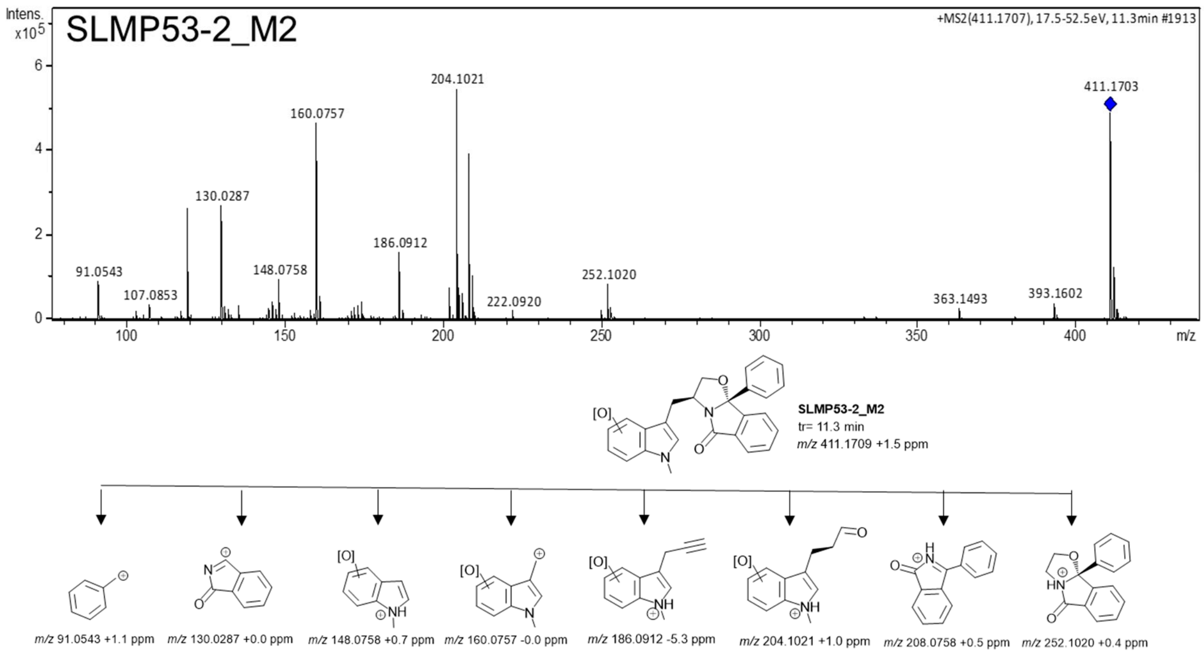


**Figure S9:** LC-ESI(+)-HRMS/MS spectrum obtained for **SLMP53-2_M2** and proposed fragmentation pattern.

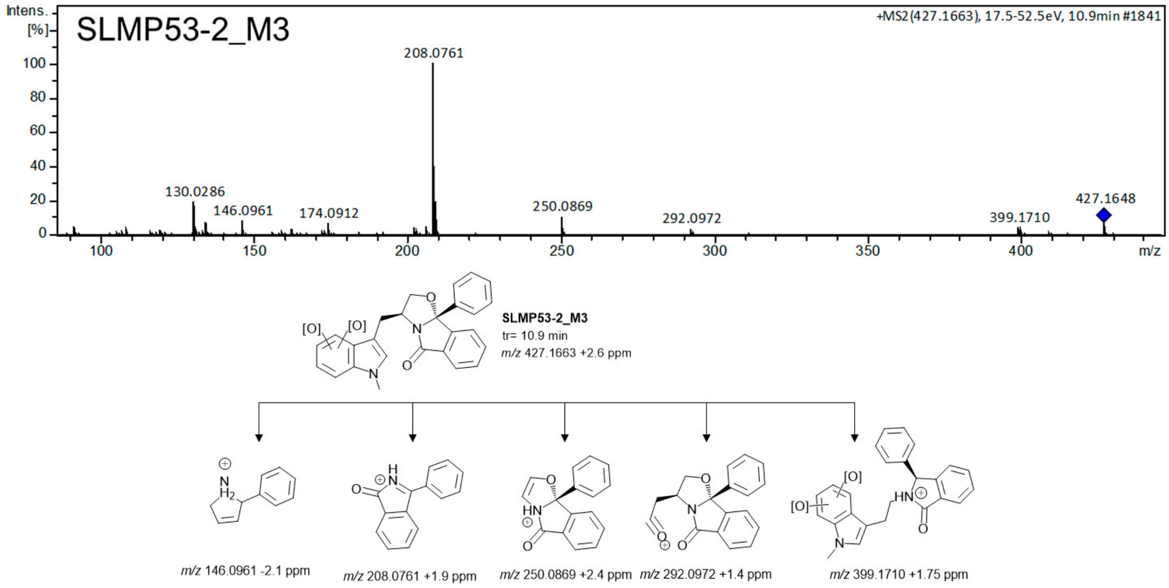


**Figure S10:** LC-ESI(+)HRMS/MS spectrum obtained for **SLMP53-2_M3** and proposed fragmentation pattern.

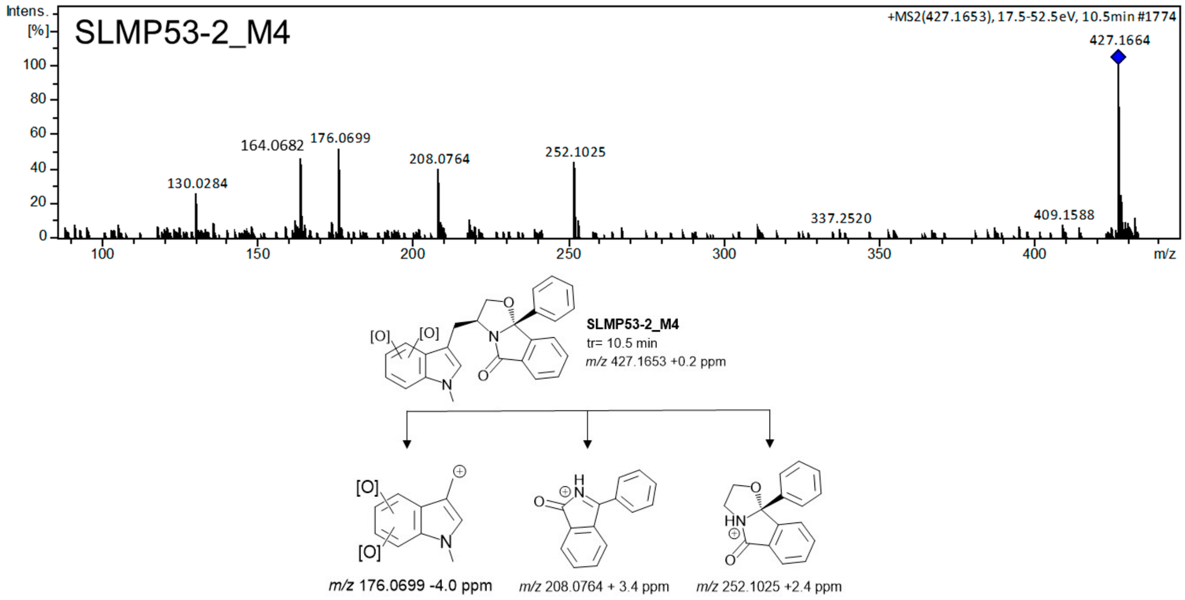


**Figure S11:** LC-ESI(+)-HRMS/MS spectrum obtained for **SLMP53-2_M4** and proposed fragmentation pattern.

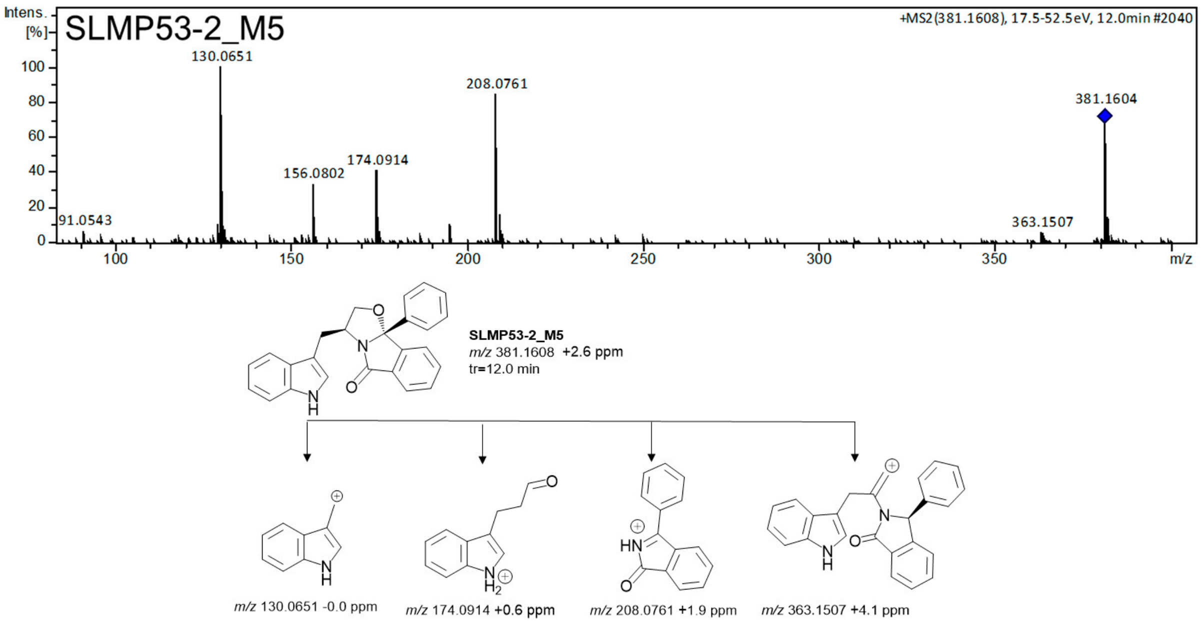


**Figure S12**: LC-ESI(+)-HRMS/MS spectrum obtained for **SLMP53-2_M5** and proposed fragmentation pattern.

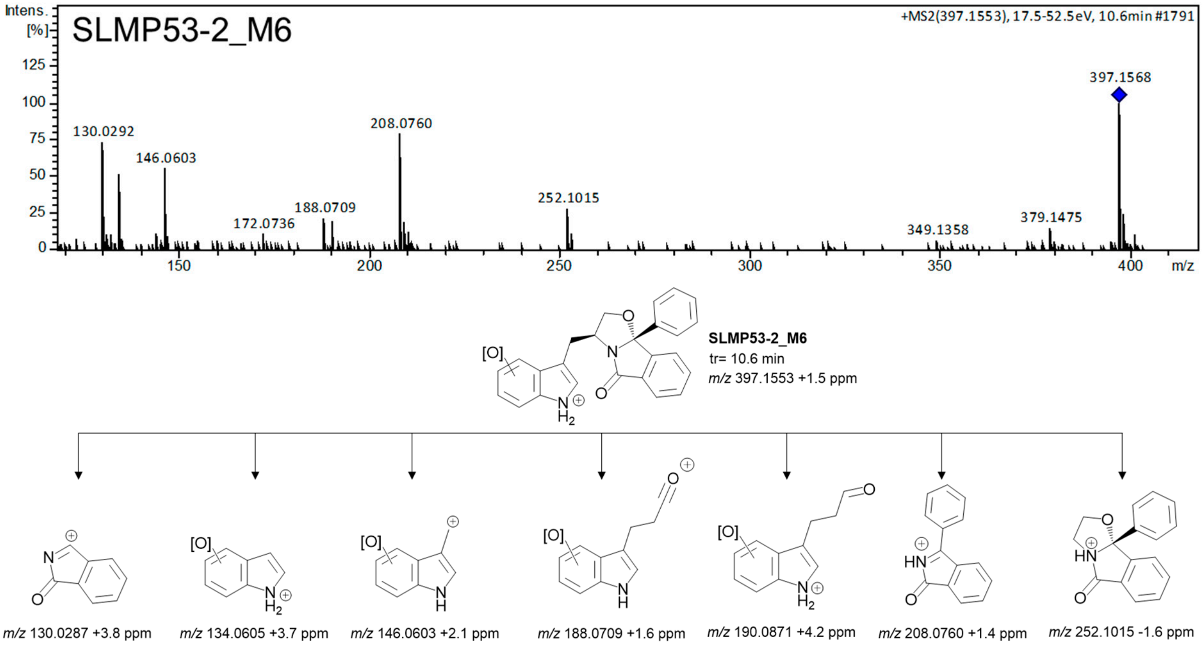


**Figure S13:** LC-ESI(+)-HRMS/MS spectrum obtained for **SLMP53-2_M6** and proposed fragmentation pattern.

# Plots of relative abundance over HLM incubation time for SLMP53-1, SLMP53-2 and their Phase I metabolites

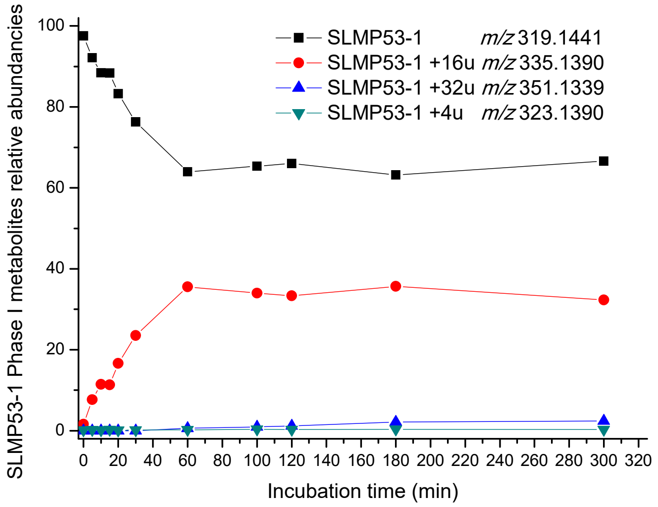


**Figure S14:** Plots of the relative abundance over incubation time for SLMP53-1 (**6**) and its Phase I metabolites.

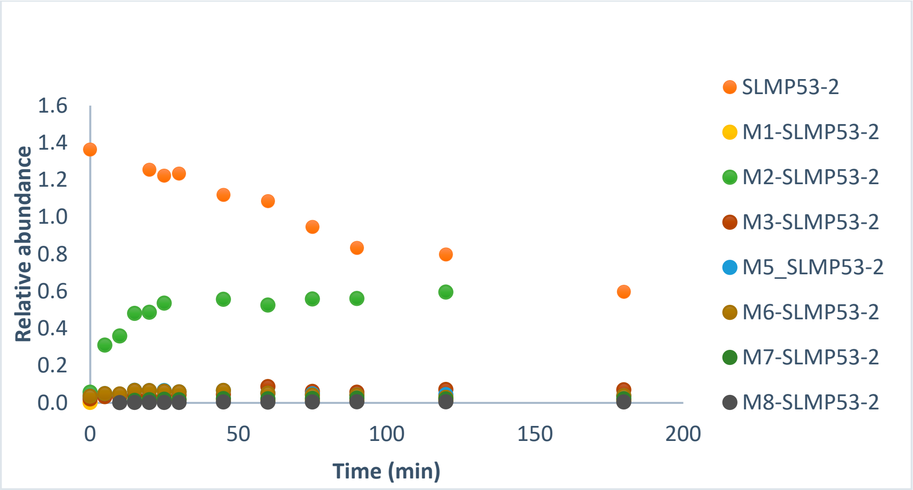


**Figure S15:** Plots of the relative abundance over incubation time for SLMP53-2 (**7**) and its Phase I metabolites.

# LC-HRMS/MS data of SLMP53-1 Phase II metabolites

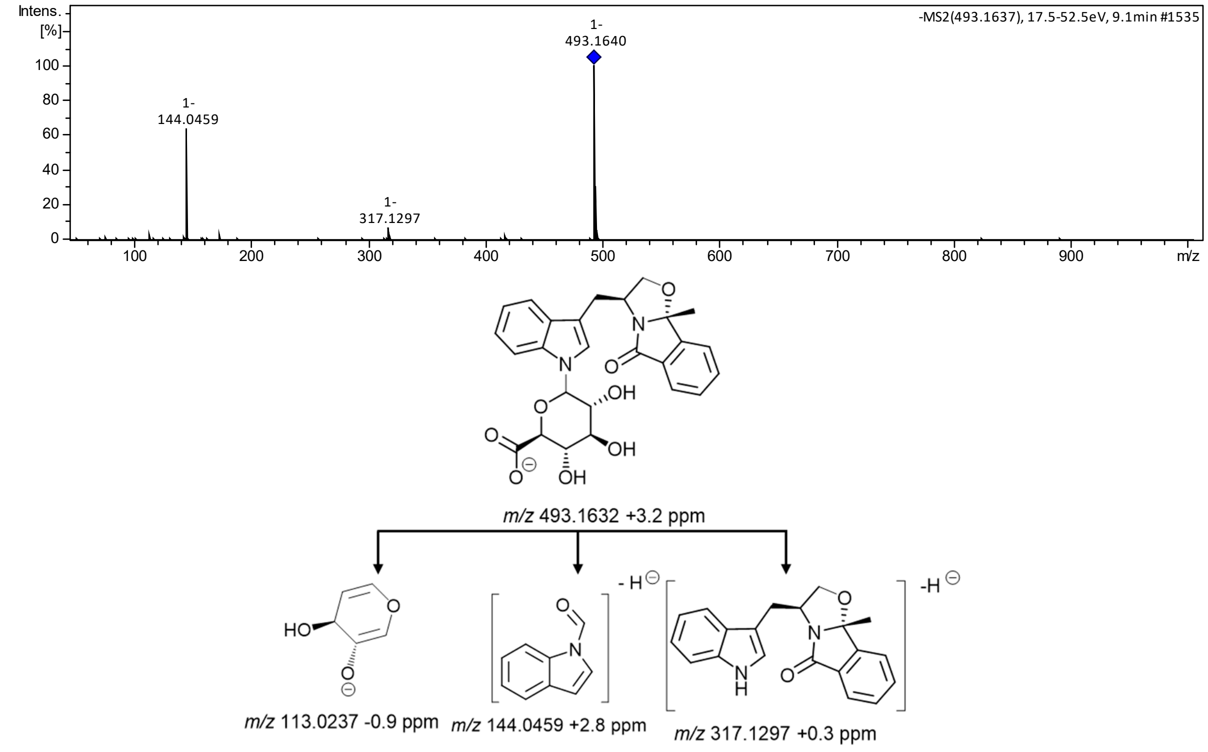


**Figure S16:** LC-ESI(-)-HRMS/MS spectrum obtained for **SLMP53-1_Glu1** and proposed fragmentation mechanism.

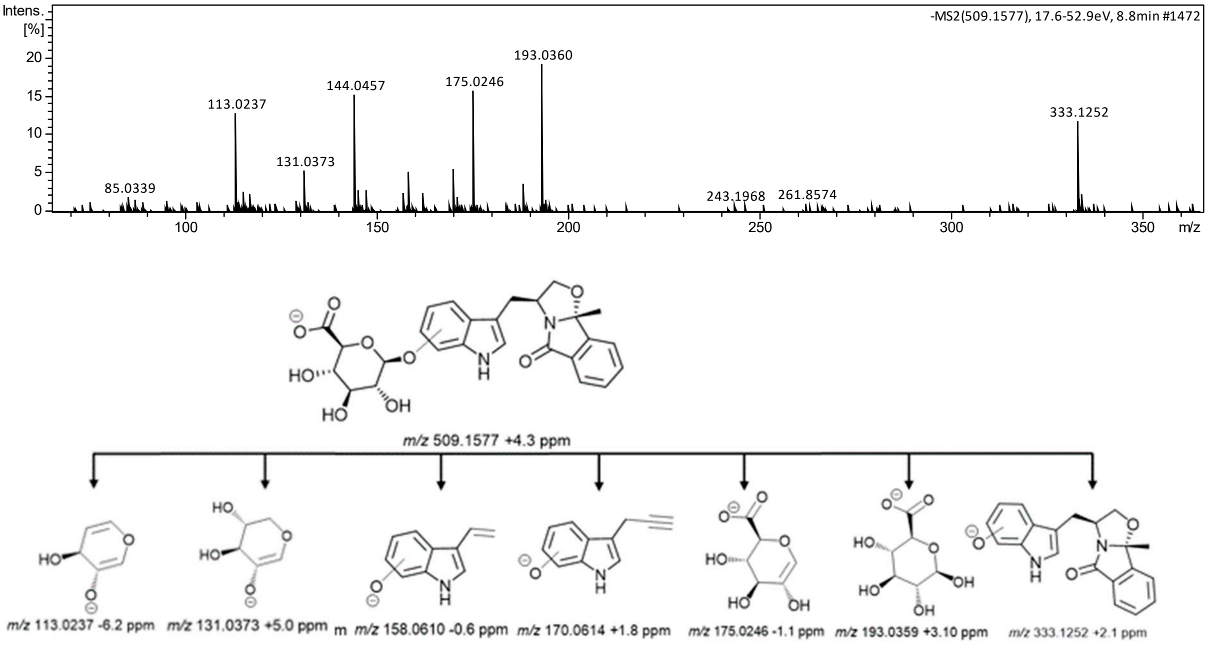


**Figure S17:** LC-ESI(-)-HRMS/MS spectrum obtained *for* **SLMP53-1_Glu2** and proposed fragmentation mechanism.

# LC-HRMS/MS data for the biomimetic reaction of SLMP53-1

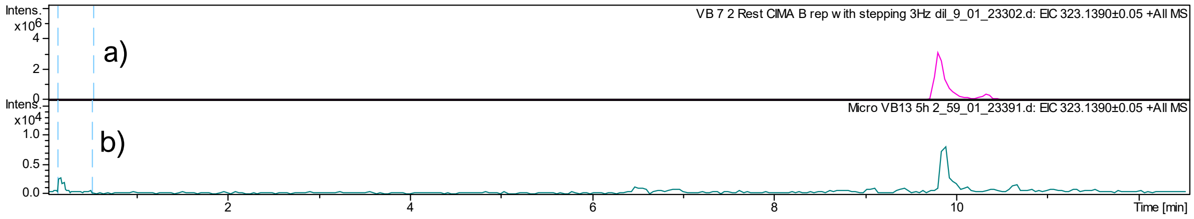


**Figure S18**: LC-ESI(+)-HRMS extracted ion chromatogram at *m/z* 323.1390, corresponding to **SLMP53-1_M5**: a) in the 24h biomimetic oxidation reaction; and b) in HLM incubations.

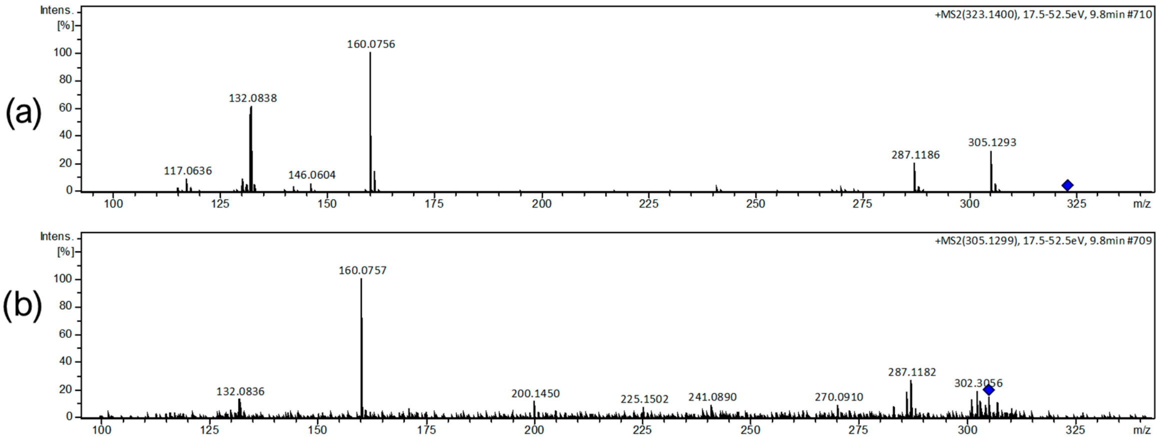


**Figure S19:** LC-ESI(+)-HRMS/MS spectrum, obtained for **SLMP53-1_M5**: a) in the 24h biomimetic oxidation reaction; and b) in HLM incubations.

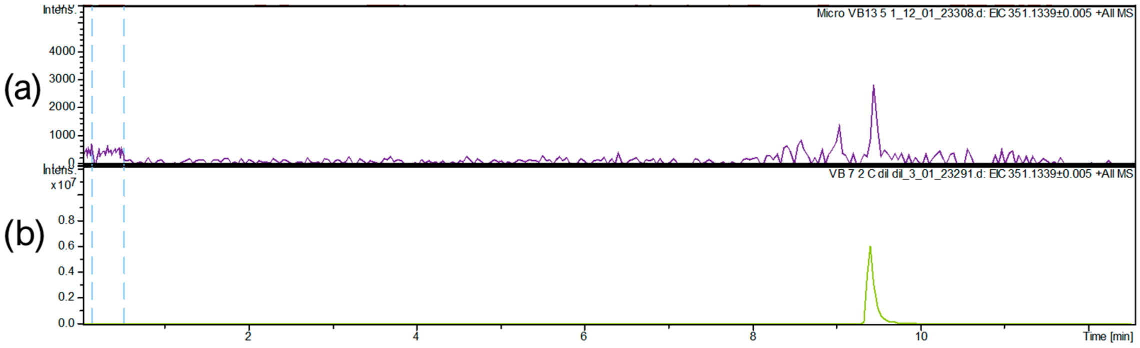


**Figure S20:** LC-ESI(+)-HRMS extracted ion chromatogram at *m/z* 351.1339, corresponding to **SLMP53-1_M6**: a) in the 24h biomimetic oxidation reaction; and b) in HLM incubations.

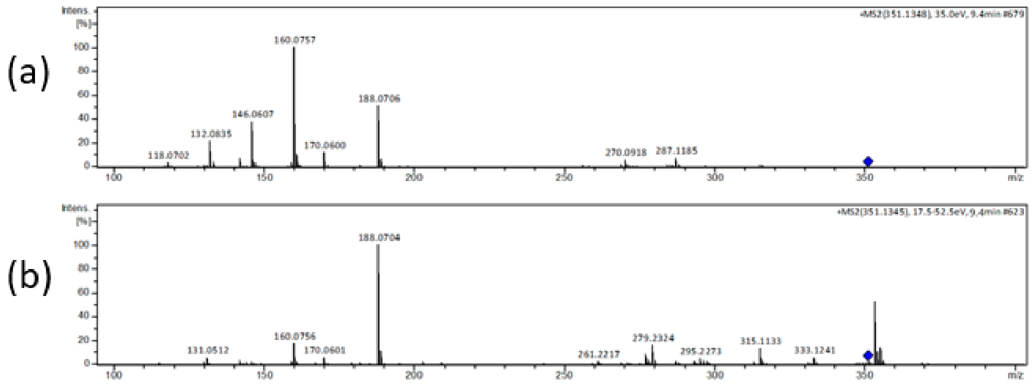


**Figure S21:** LC-ESI(+)-HRMS/MS spectra, obtained for **SLMP53-1_M6**: a) in the biomimetic oxidation; and b) in the HLM incubations.

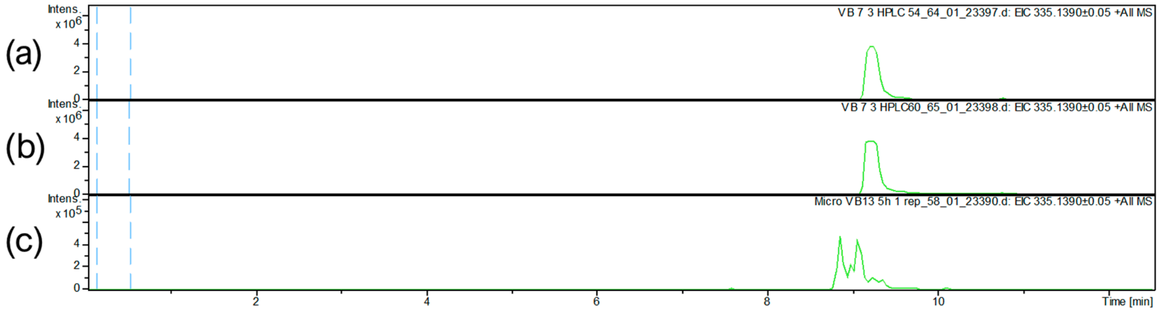


**Figure S22:** LC-ESI(+)-HRMS extracted ion chromatogram at *m/z* 335.1390, corresponding to: a) **SLMP53-1_M3a** and b) **SLMP53-1_M3b**, obtained in the 5h biomimetic oxidation of SLMP53-1 (**6**); and c) **SLMP53-1_M1**, **SLMP53-1_M2** and **SLMP53-1_M3** detected in HLM incubations. Products of biomimetic oxidation SLMP53-1_M3a and SLMP53-1_M3b show compatible retention times with one major Phase I metabolite SLMP53-1_M3 observed in the HLM incubations.

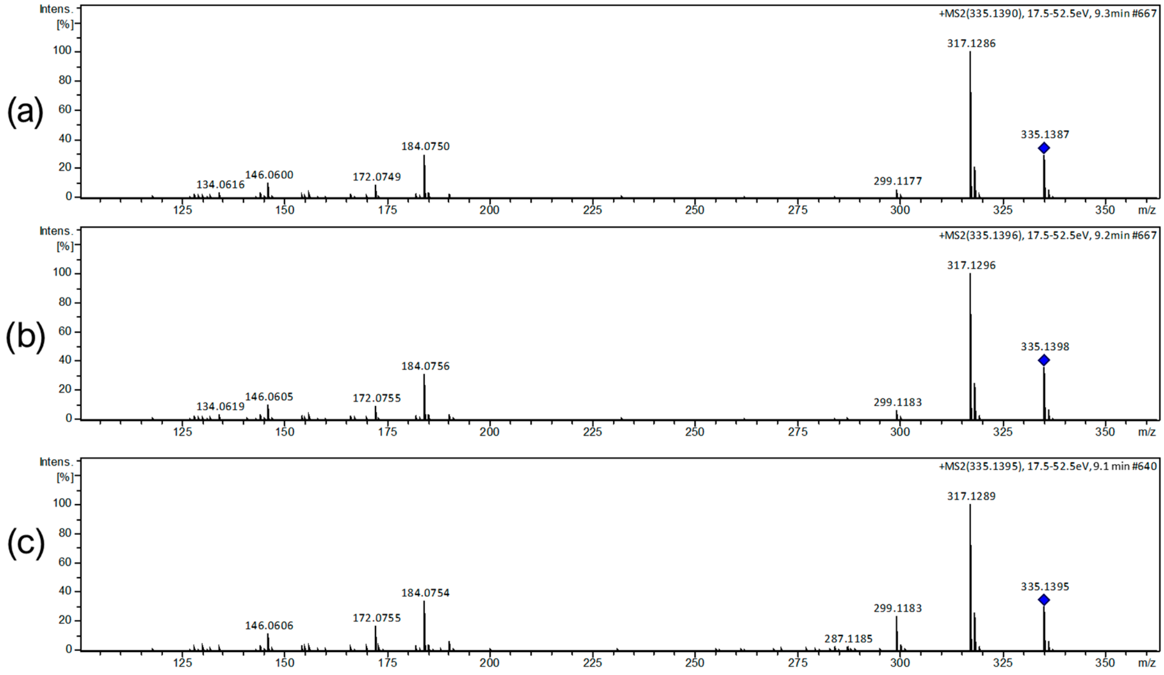


**Figure S23:** LC-ESI(+)-HRMS/MS spectra, obtained for: a) **SLMP53-1_M3a**; and b) **SLMP53-1_M3b** in the biomimetic oxidation reaction; and c) **SLMP53-1_M3** detected in the HLM incubations.

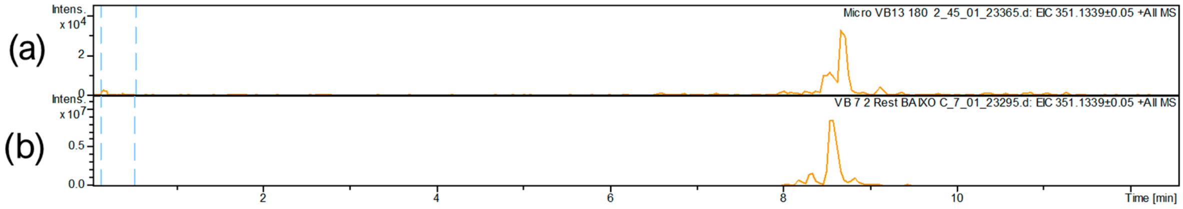


**Figure S24:** LC-ESI(+)-HRMS extracted ion chromatogram at *m/z* 351.1339, corresponding to a) **SLMP53-1_M4** and, **SLMP53-1_M5** detected in HLM incubations; and b) **SLMP53-1_M4** detected in the biomimetic oxidation of hit compound SLMP53-1 (**6**).

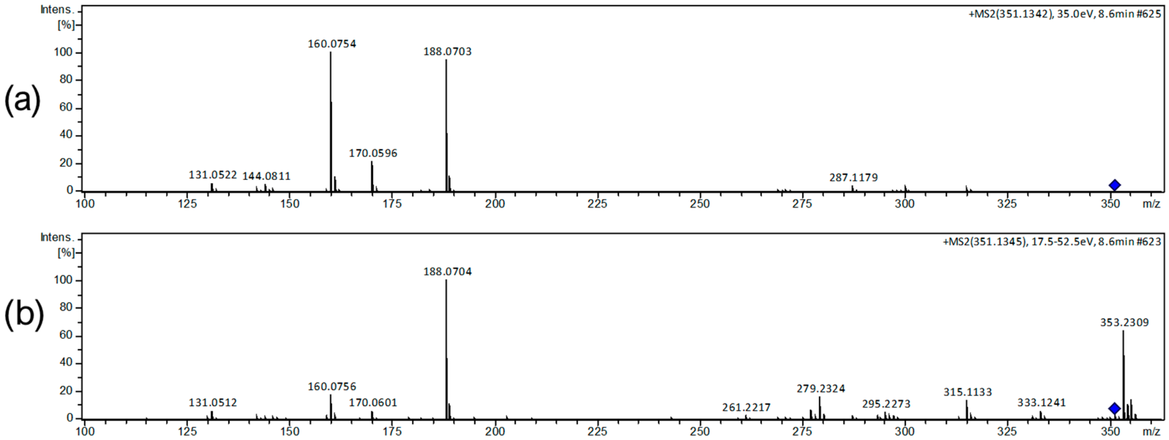


**Figure S185:** LC-ESI(+)-HRMS/MS spectra obtained for **SLMP53-1_M4** in: a) the biomimetic oxidation reaction; and b) the HLM incubations.

# $^{1}$H NMR and $^{13}$C NMR spectra of SLMP53-1 Phase I metabolites

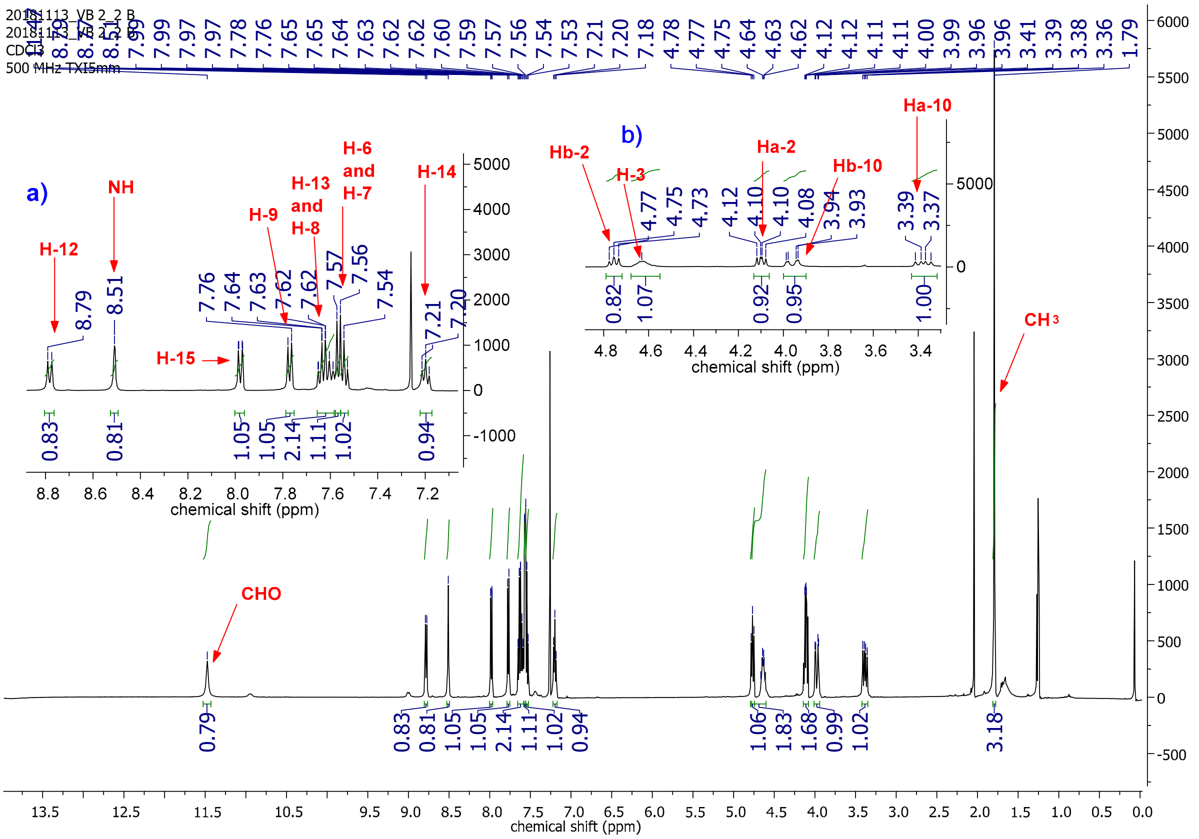


**Figure S26:** $^{1}$H NMR spectrum, obtained in $CDCl_3$, of **SLMP53-1_M5**; a) Expansion between 8.8 ppm and 7.1 ppm; and b) Expansion of $^{1}$H NMR spectrum between 4.8 ppm and 3.4 ppm.

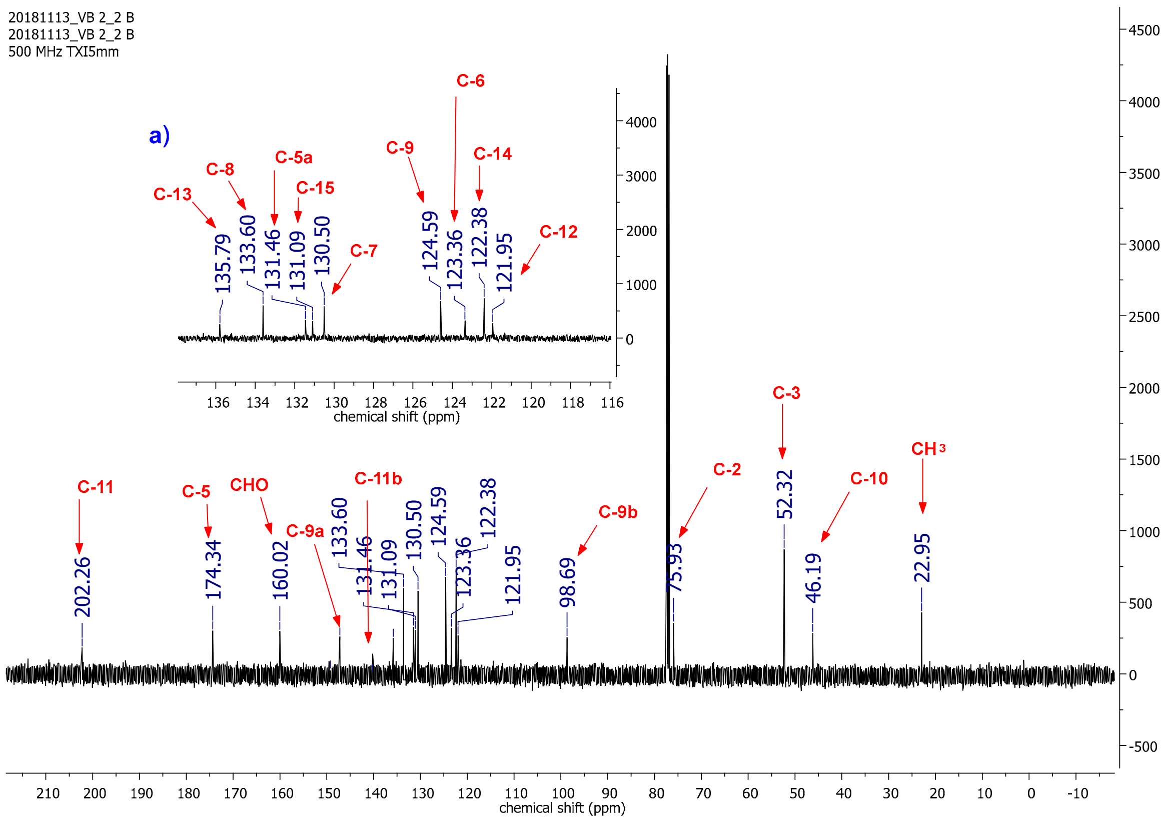


**Figure S27:** $^{13}$C NMR spectrum, obtained in $CDCl_3$, of **SLMP53-1_M5**; a) Expansion of $^{13}$C NMR spectrum between 138 ppm and 116 ppm.

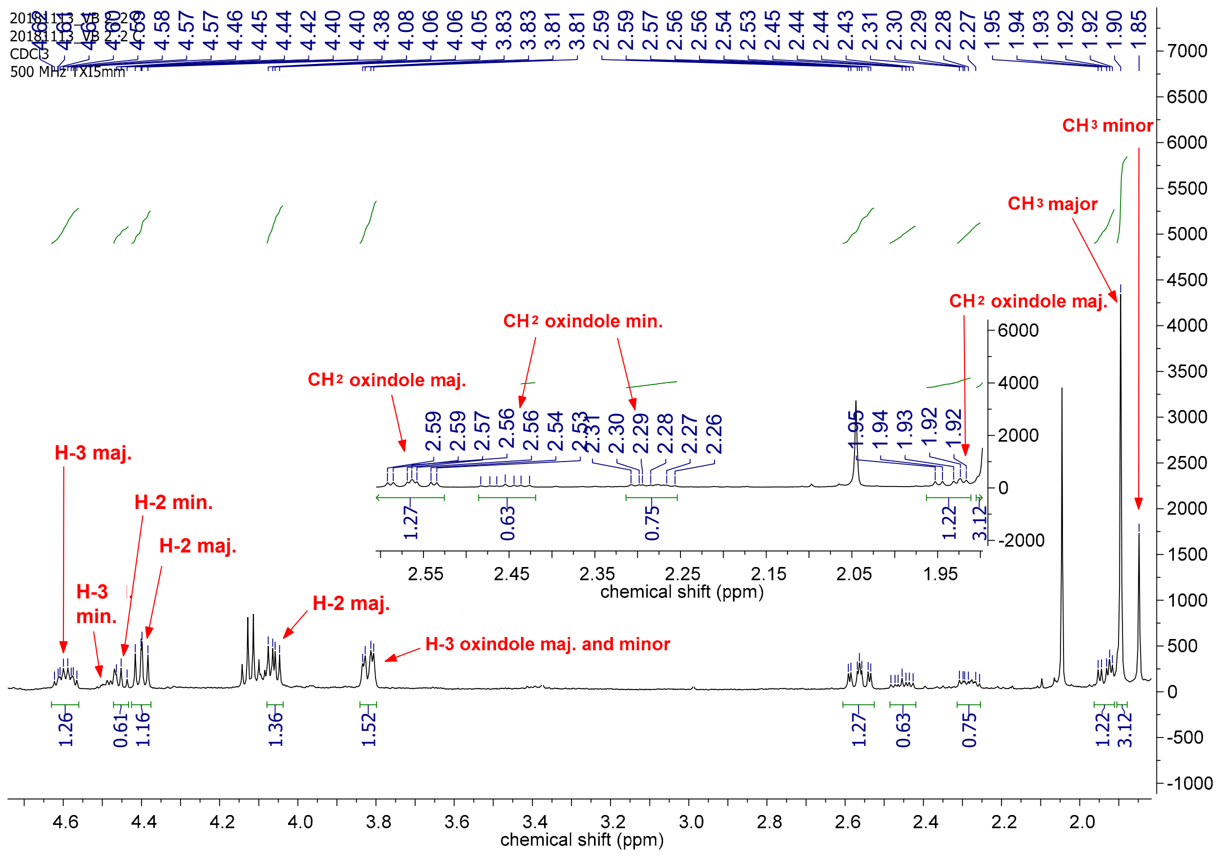


**Figure S28:** Expansion between 4.6 and 2.0 ppm, obtained in $CDCl_3$, of the diastereomers mixture **SLMP53-1_M3a** and **SLMP53-1_M3b**.

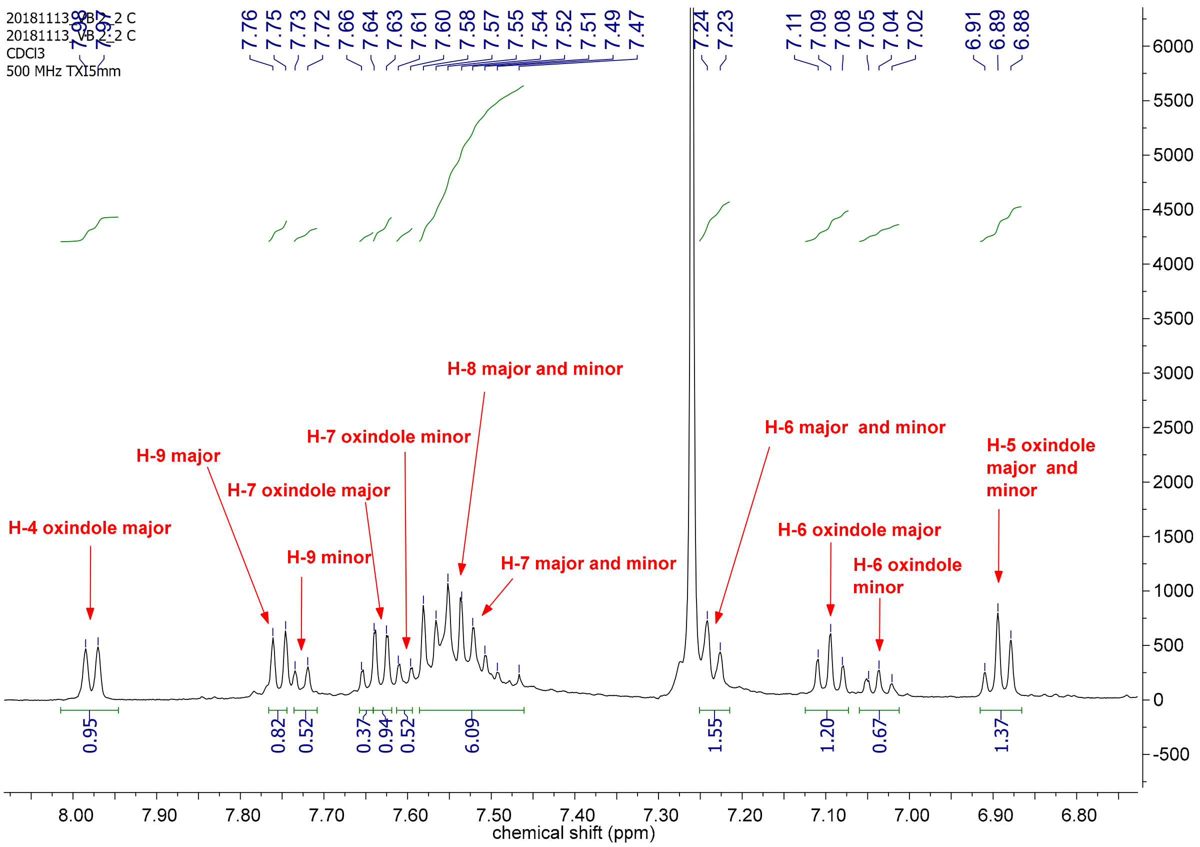


**Figure S29:** Expansion between 8.1 and 6.8 ppm of $^{1}$H NMR spectrum, obtained in $CDCl_3$, for the diastereomers mixture **SLMP53-1_M3a** and **SLMP53-1_M3b**.

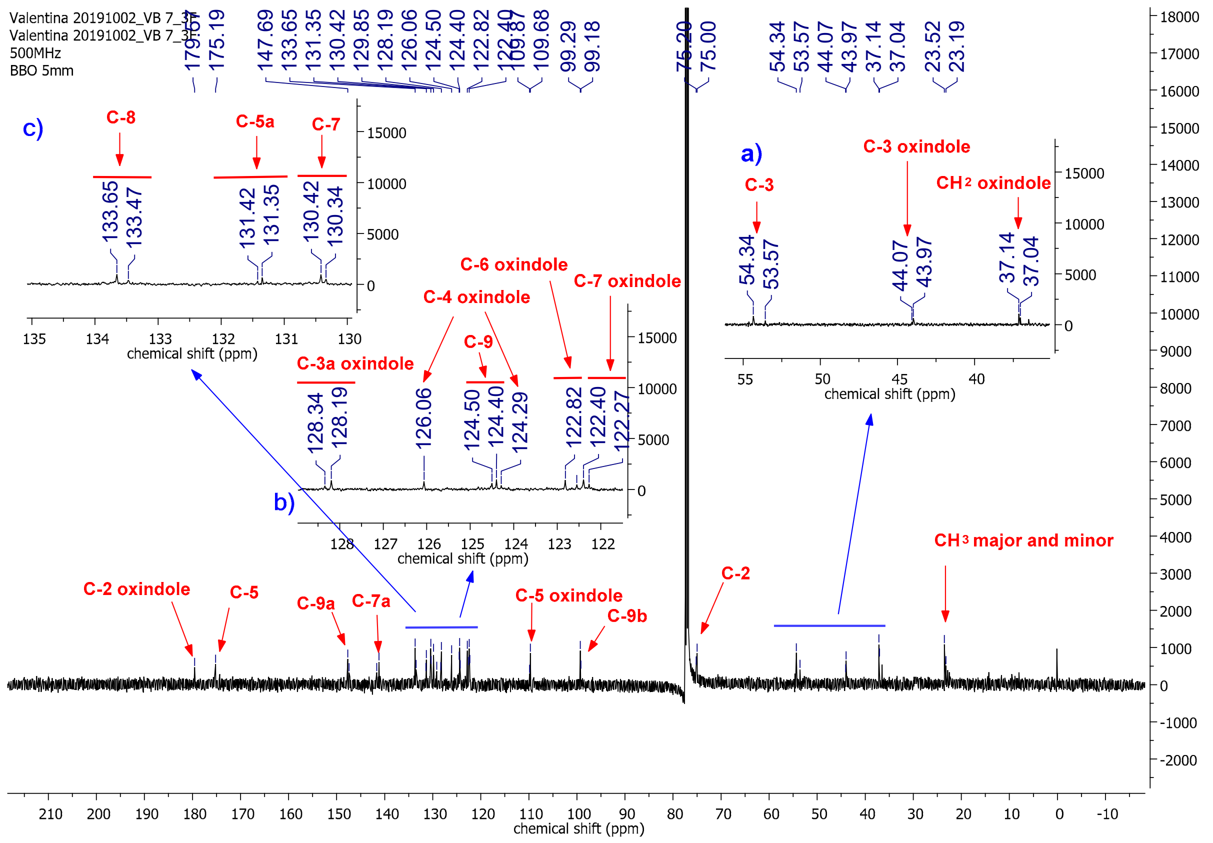


**Figure S30:** $^{13}$C NMR spectrum, obtained in $CDCl_3$ for the diastereomers mixture **SLMP53-1_M3a** and **SLMP53-1_M3b**; a) $^{13}$C NMR spectrum expansion between 55 ppm and 30 ppm; b) $^{13}$C NMR spectrum expansion between 130 ppm and 122 ppm; c) $^{13}$C NMR spectrum expansion between 135 ppm and 130 ppm.

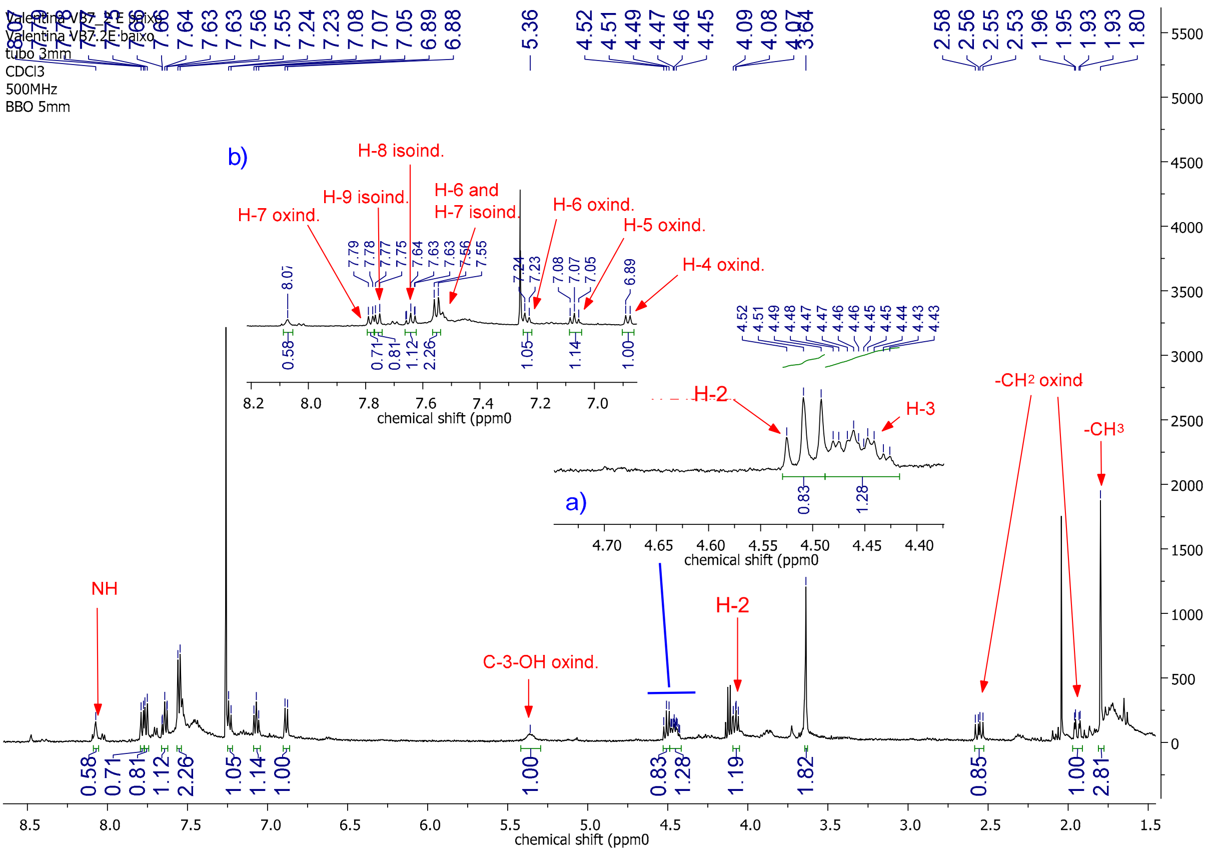


**Figure S31:** Expansion between 8.5 and 1.5 ppm of $^{1}H$ NMR spectrum obtained in $CDCl_3$ for **SLMP53-1_M4**; a) Expansion between 4.70 and 4.40 ppm; b) Expansion between 8.2 and 7.0 ppm.

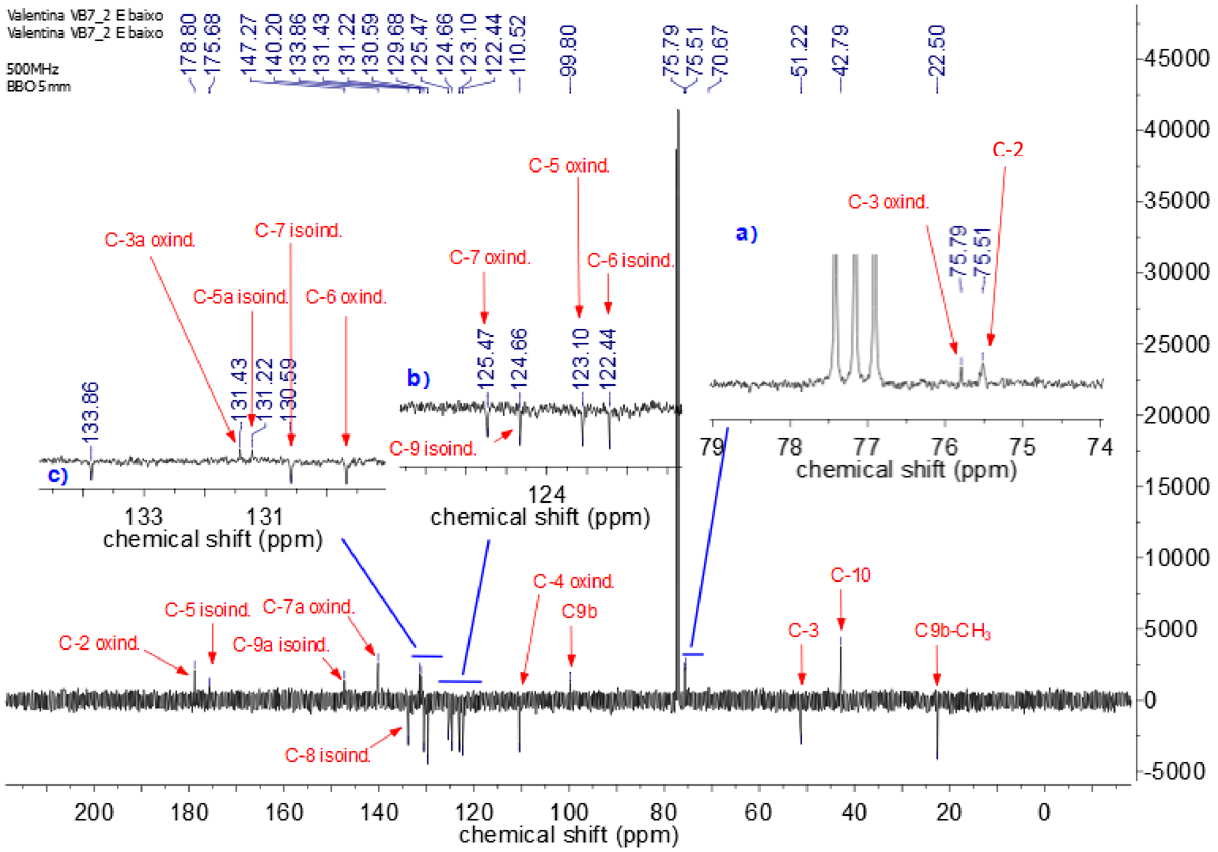


**Figure S32:** $^{13}$C NMR spectrum obtained in $CDCl_3$ for **SLMP53-1_M4**; a) Expansion from 79.0 to 74.0 ppm; b) Expansion from 125.5 ppm and 122.0 ppm; c) Expansion from 134.0 ppm to 130.0 ppm.

# LC-HRMS/MS data of SLMP53-1 glutathione adducts

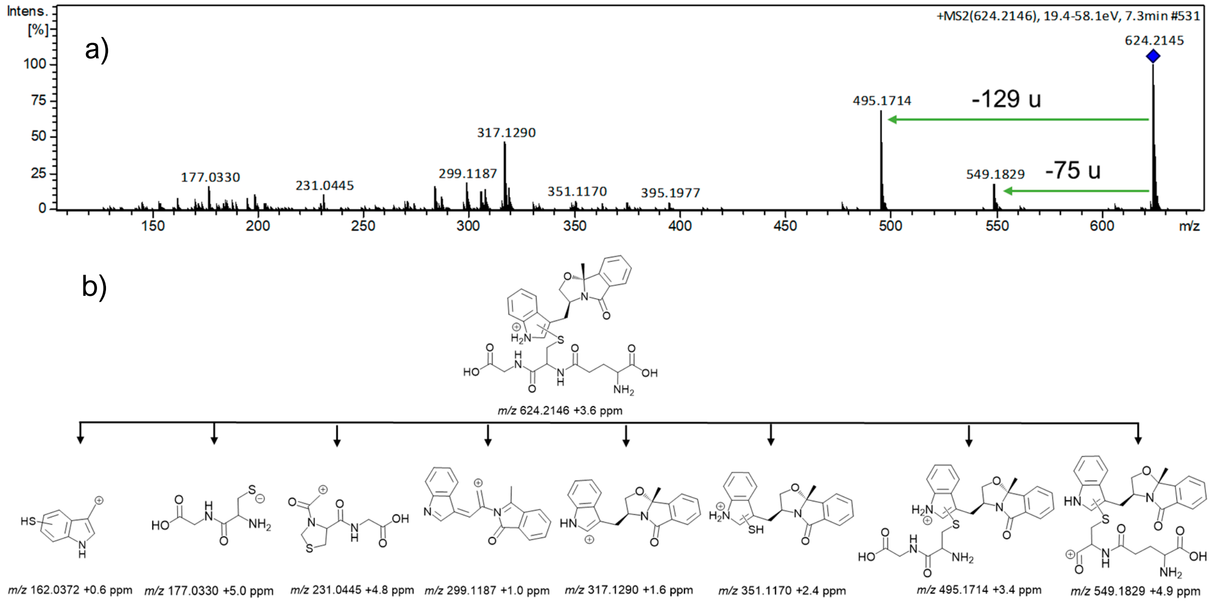


**Figure S33:** a) LC-ESI(+)-HRMS/MS spectrum obtained for **SLMP53-1-GS1**; and b) Proposed fragmentation pattern.

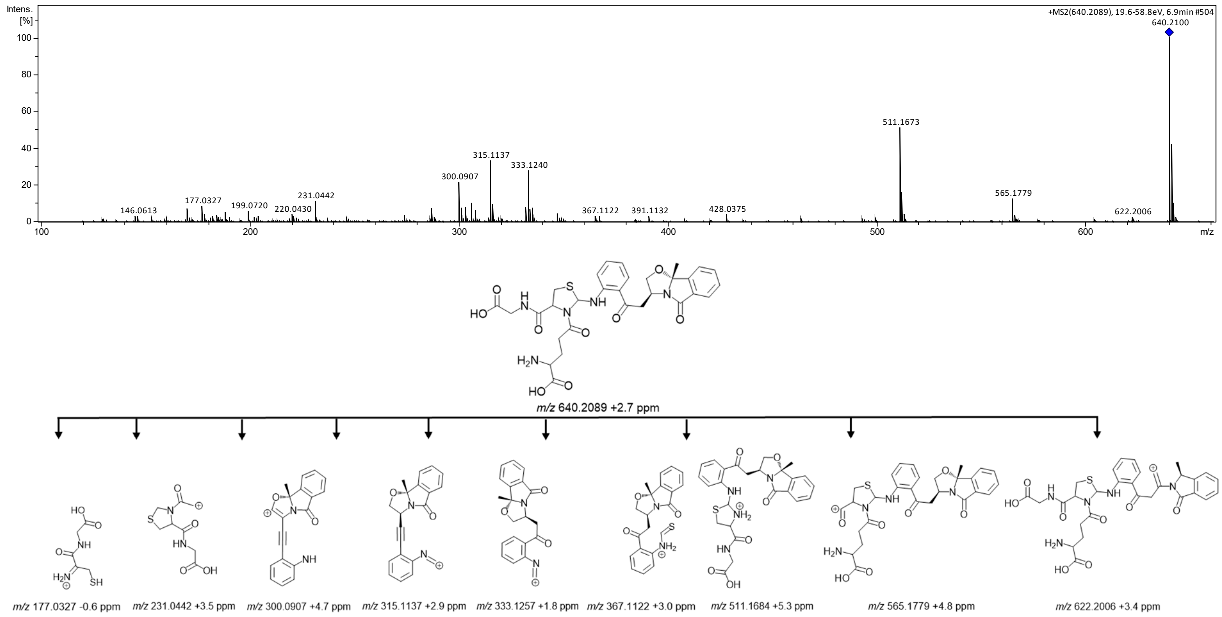


**Figure S34:** LC-ESI(+)-HRMS/MS spectra obtained for **SLMP53-1-GS2** and proposed fragmentation pattern.

# $^1H$ NMR and $^{13}C$ NMR spectra of 13d and 13k

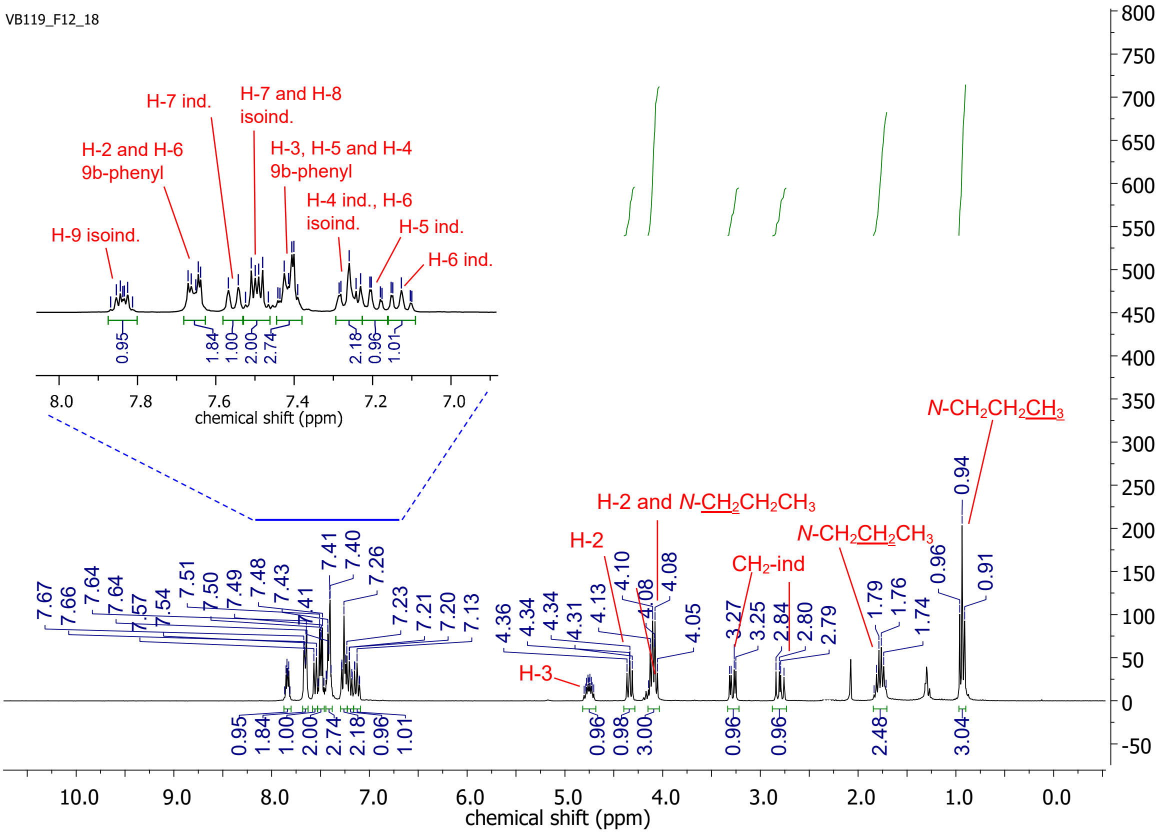


**Figure S35**: $^1H$ NMR spectrum of compound **13d**, obtained in $CDCl_3$.

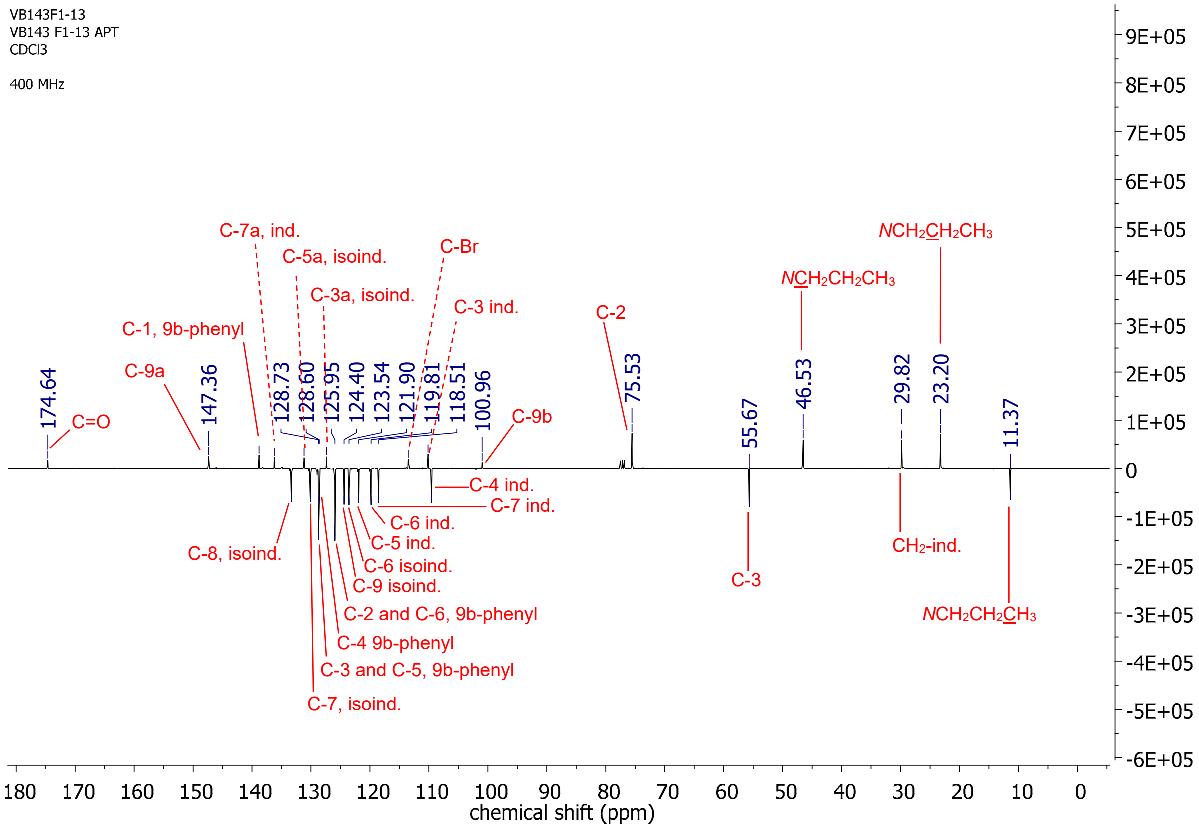


**Figure S36:** $^{13}C$ APT NMR spectrum of compound **13d**, obtained in $CDCl_3$.

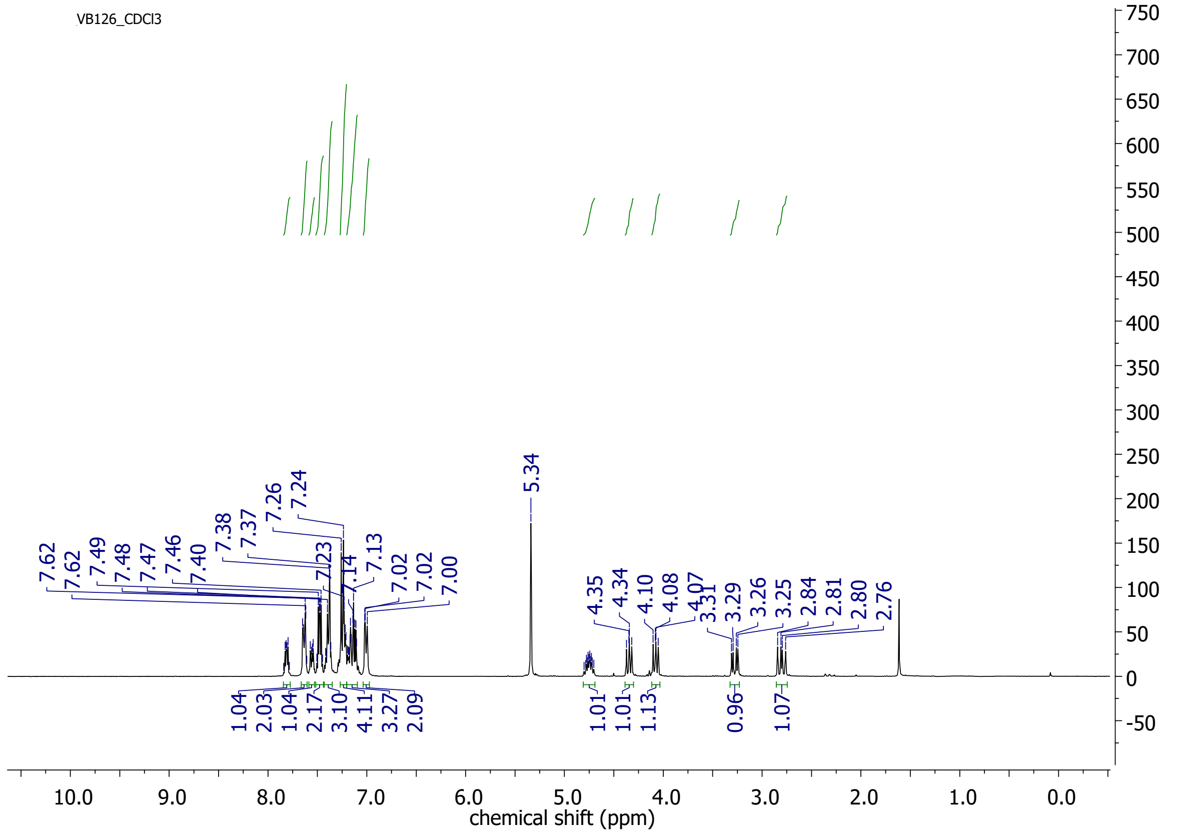


**Figure S37:** $^{1}H$ NMR spectrum of compound **13k,** obtained in $CDCl_3$.

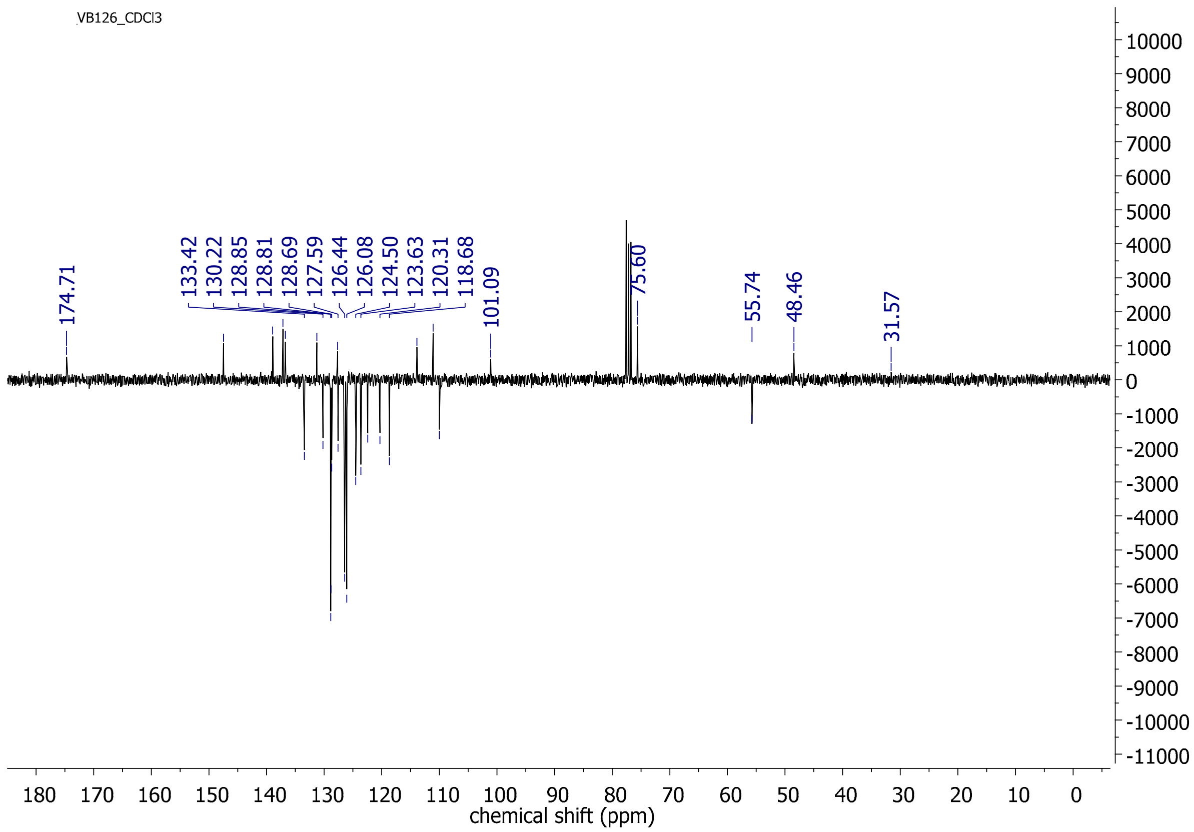


**Figure S38:** $^{13}C$ APT NMR spectrum of compound **13k,** obtained in in $CDCl_3$.

# HPLC data of compounds 13d and 13k

Page 1 of 1

**Area % Report**

Data File: C:\Documents and Settings\hplc\Desktop\Valentina\VB119 e 126\VB119 10-3M
Method: C:\EZChrom Elite\Enterprise\Projects\Default\Method\untitled.met
Acquired: 23-07-2020 18:42:45
Printed: 12-12-2022 14:29:18

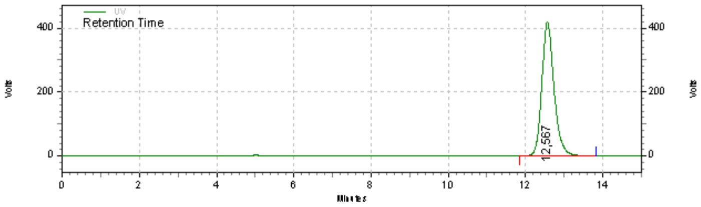


**UV Results**

| Retention Time | Area | Area % | Height | Height % |
|---|---|---|---|---|
| 12,567 | 36751300 | 100,00 | 1677826 | 100,00 |
| Totals | 36751300 | 100,00 | 1677826 | 100,00 |

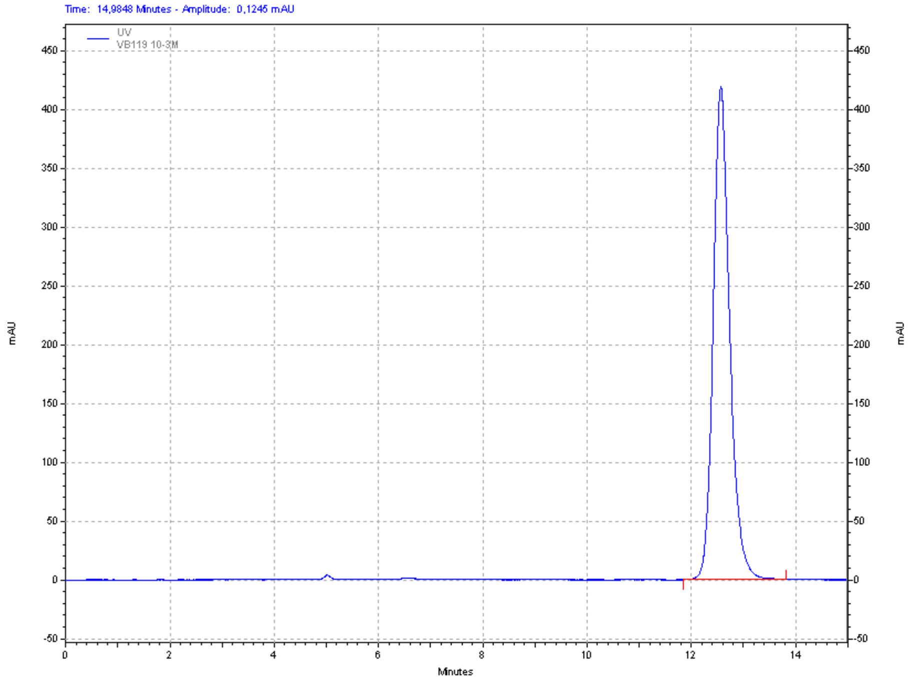


**Figure S39:** HPLC-DAD spectrum of compound **13d** at concentration $10^{-3}$ M.

Page 1 of 1

**Area % Report**

Data File: C:\Documents and Settings\hplc\Desktop\Valentina\VB119 e 126\VB126E-3M.dat
Method: C:\EZChrom Elite\Enterprise\Projects\Default\Method\untitled.met
Acquired: 03-08-2020 19:21:42
Printed: 12-12-2022 14:40:53

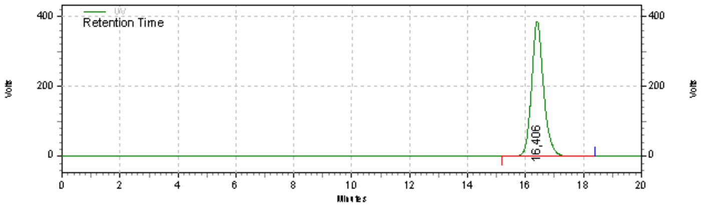


**UV Results**

| Retention Time | Area | Area % | Height | Height % |
|---|---|---|---|---|
| 16,406 | 44383749 | 100,00 | 1541953 | 100,00 |
| Totals | 44383749 | 100,00 | 1541953 | 100,00 |

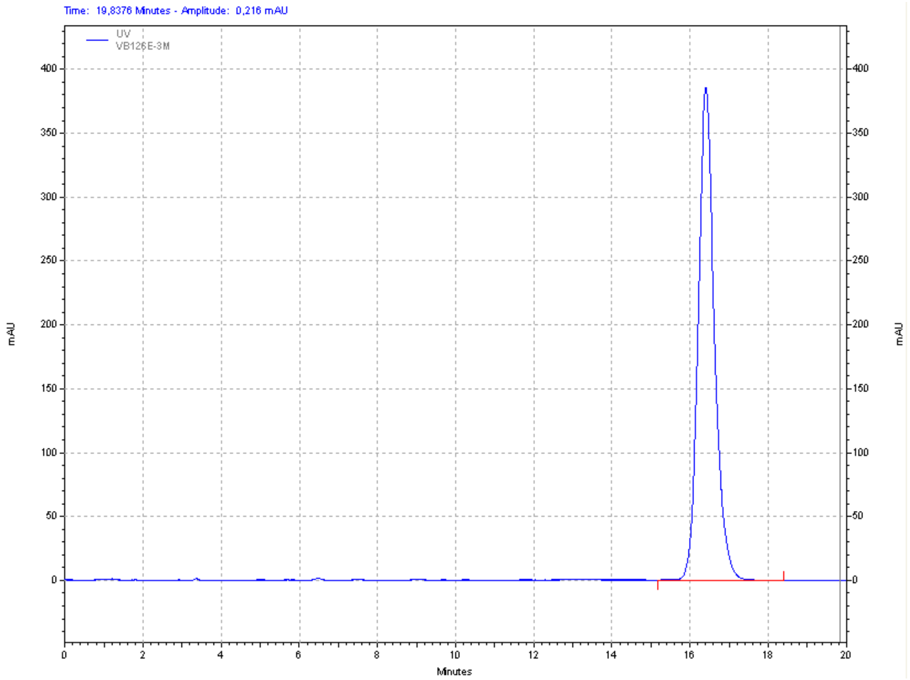


**Figure S40**: HPLC spectrum of compound **13d** at concentration $10^{-3}$ M.

# LC-HRMS/MS data of compounds 13d and 13k and their Phase I metabolites

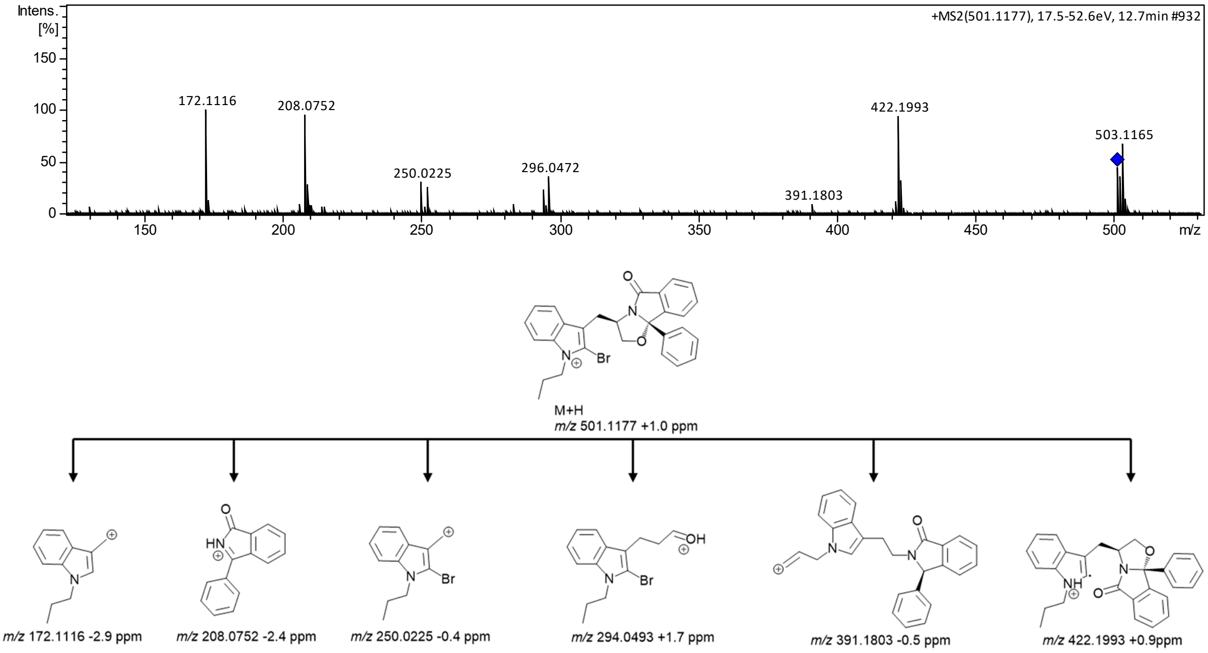


**Figure S41:** LC-ESI(+)-HRMS/MS spectra obtained for **13d** and proposed fragmentation pattern.

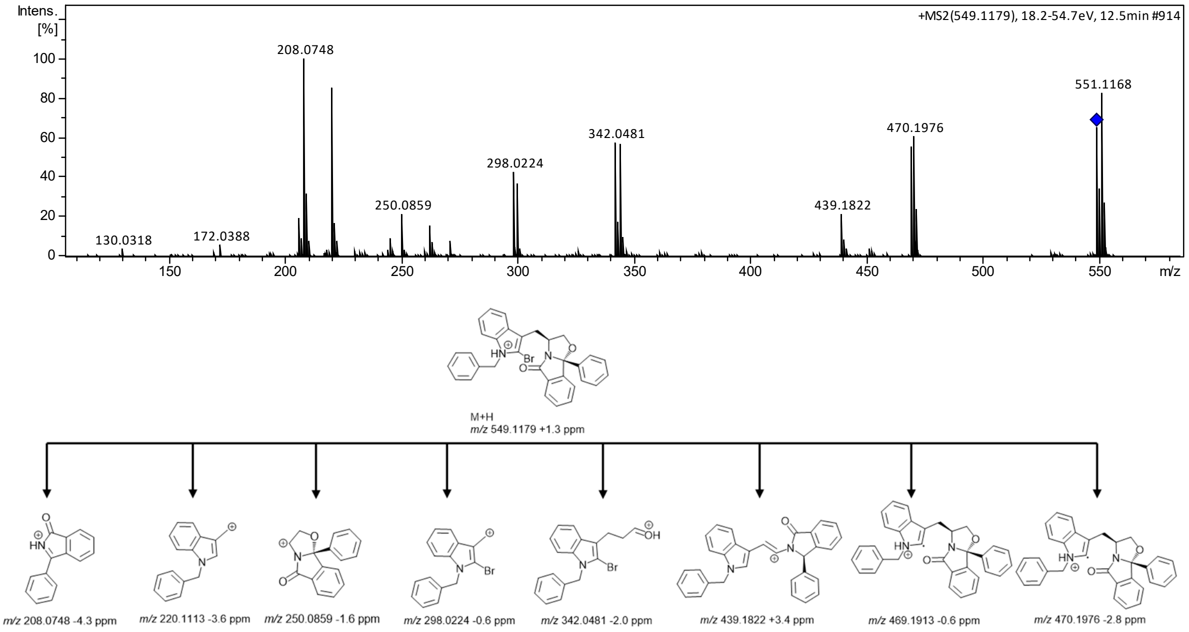


**Figure S42:** LC-ESI(+)-HRMS/MS spectra obtained for **13k** and proposed fragmentation pattern.

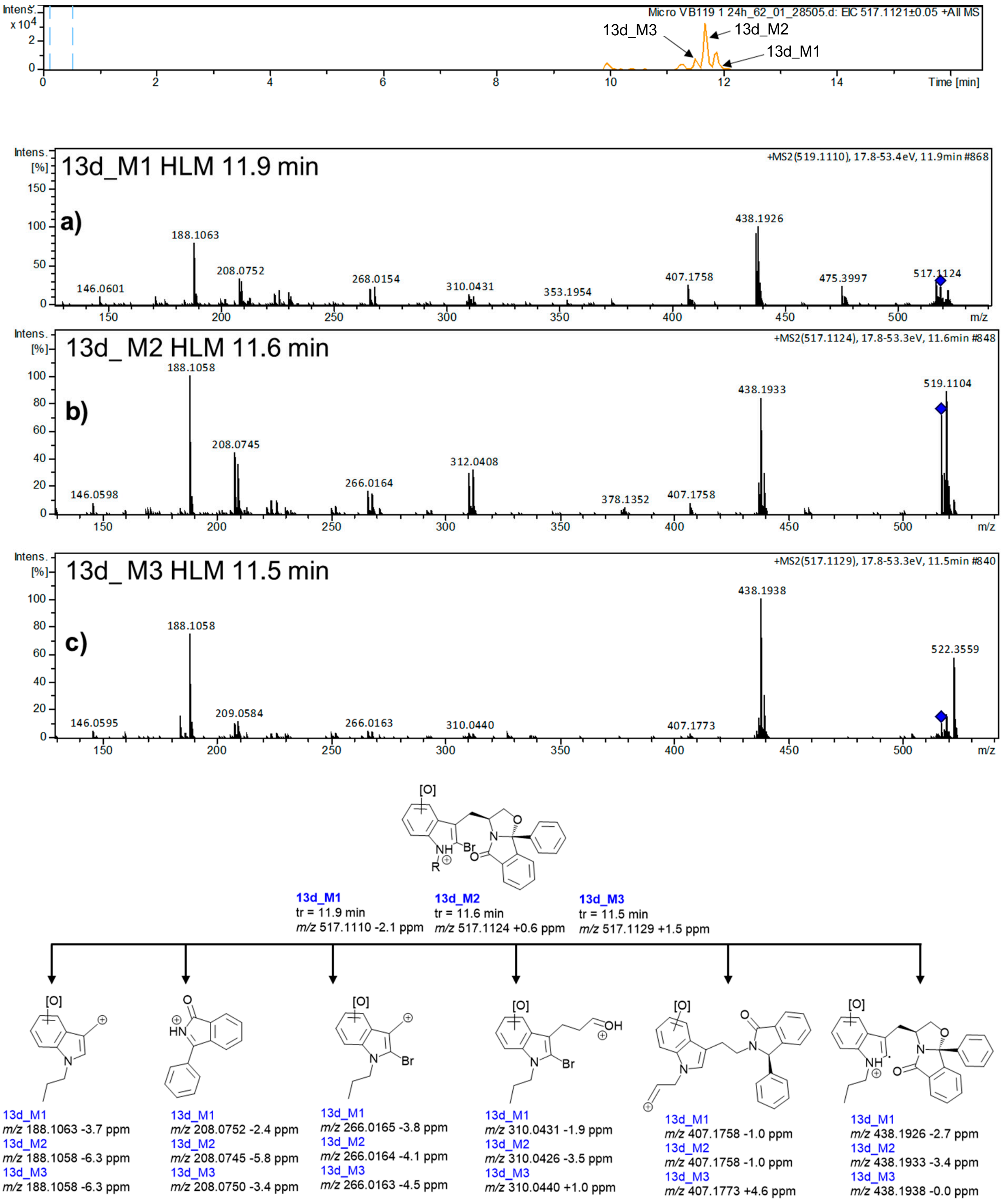


**Figure S43:** Extracted ion HRMS-ESI(+) chromatogram at *m/z* 517.1121, obtained for **13d** HLM incubations and LC-ESI(+)-HRMS/MS spectra obtained for a) **13d_M1**, b) **13d_M2**, and c) **13d_M3**. Proposed fragmentation pattern.

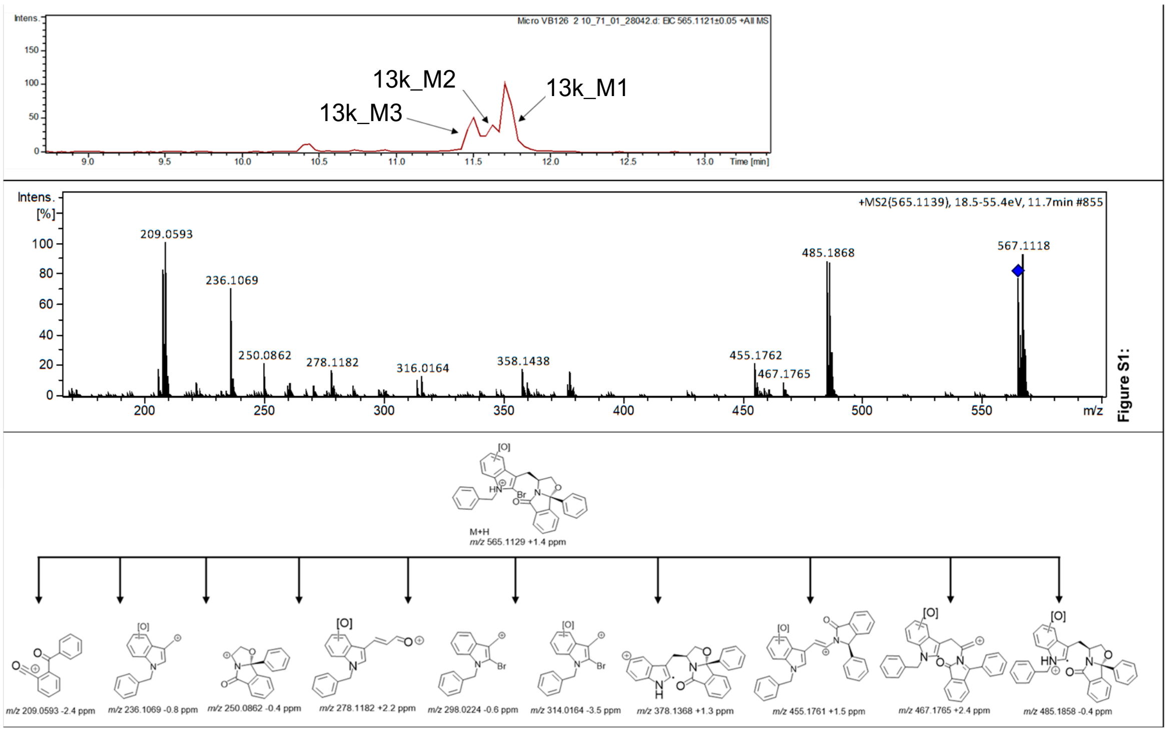


**Figure S44:** Extracted ion HRMS-ESI(+) chromatogram at *m/z* 565.1121 corresponding to **13k_M1-M3** in HLM incubations. HRMS/MS spectrum and proposed fragmentation pattern.

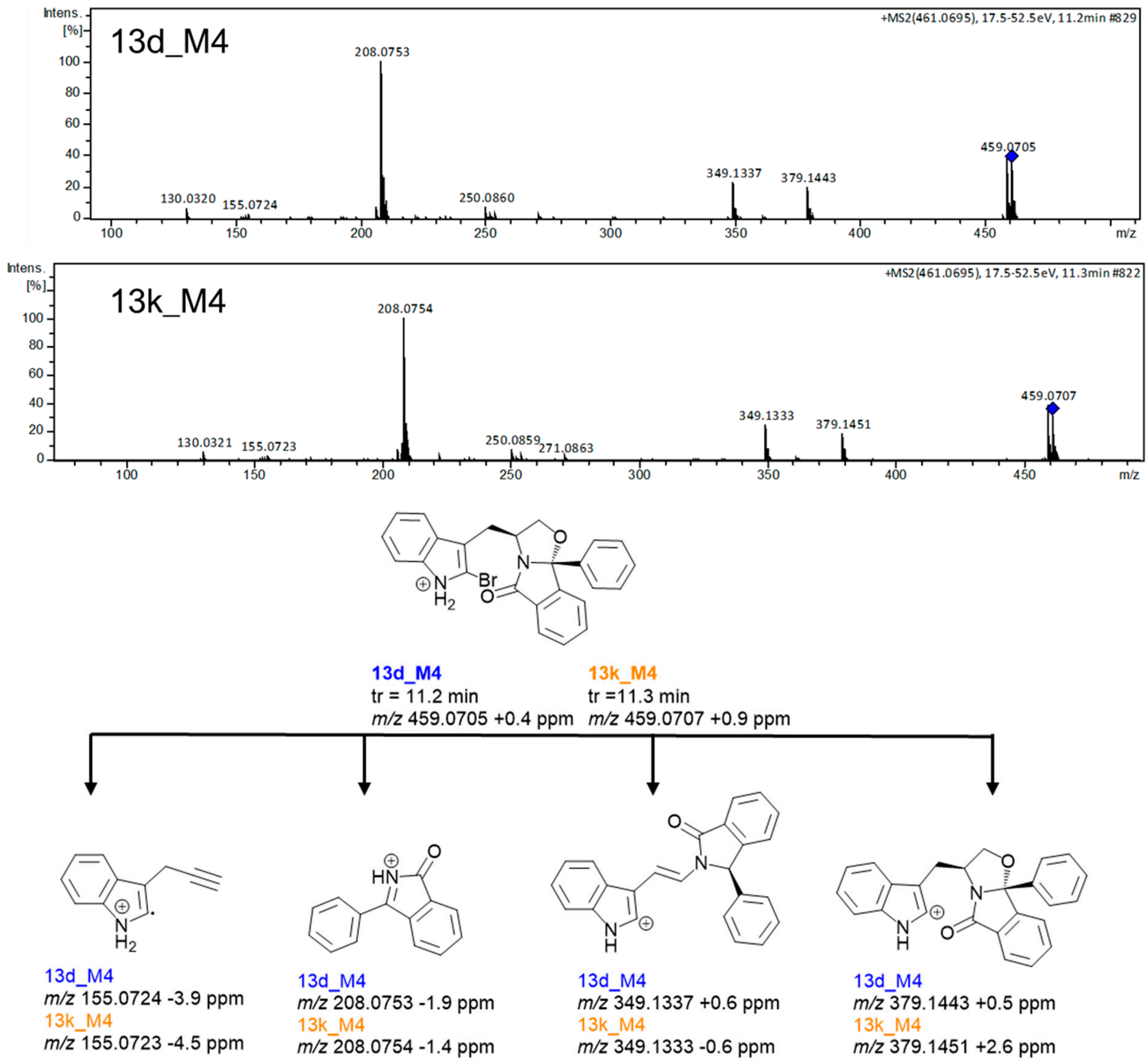


**Figure S45:** LC-ESI(+)-HRMS/MS spectra obtained for **13d_M4** and **13k_M4**, and proposed fragmentation pattern.

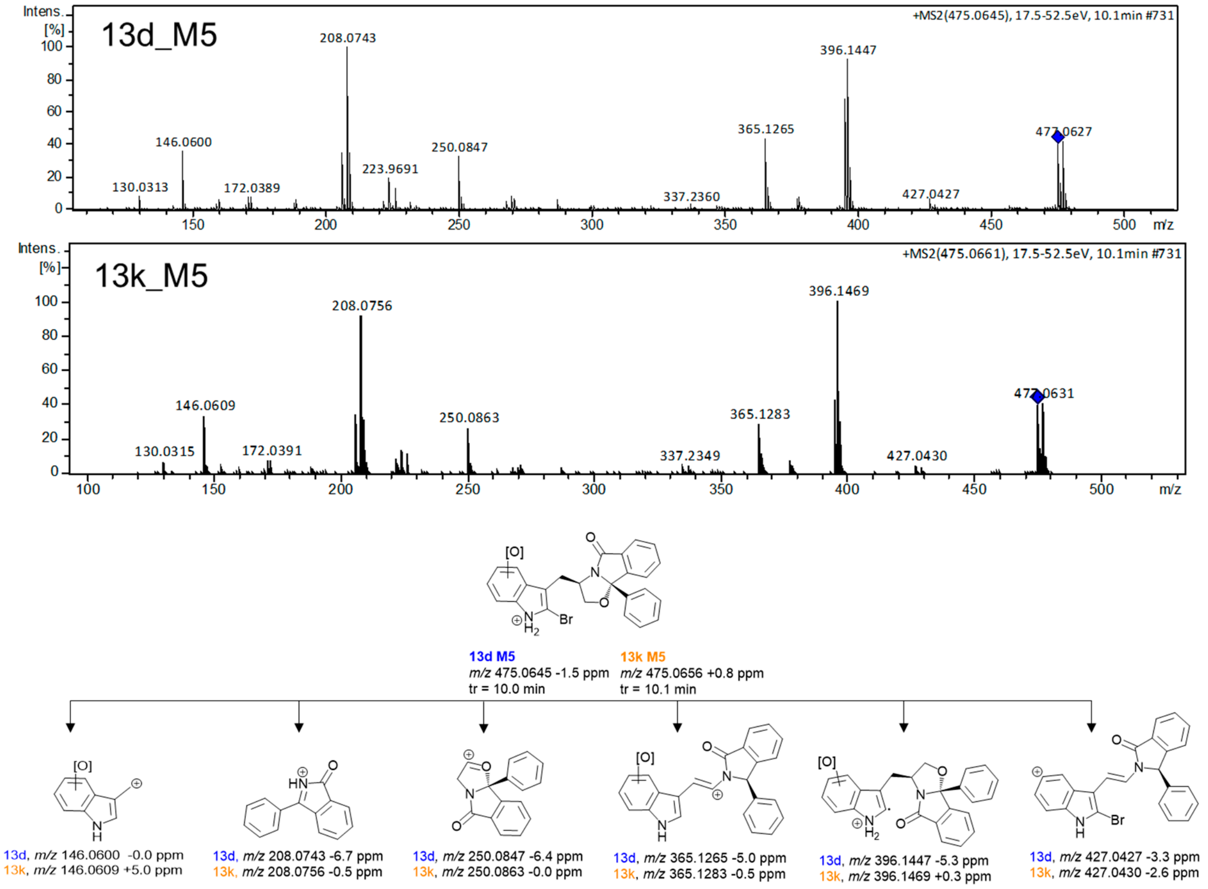


**Figure S19:** LC-ESI(+)-HRMS/MS spectra obtained for **13d_M5** and **13k_M5**, and proposed fragmentation pattern.

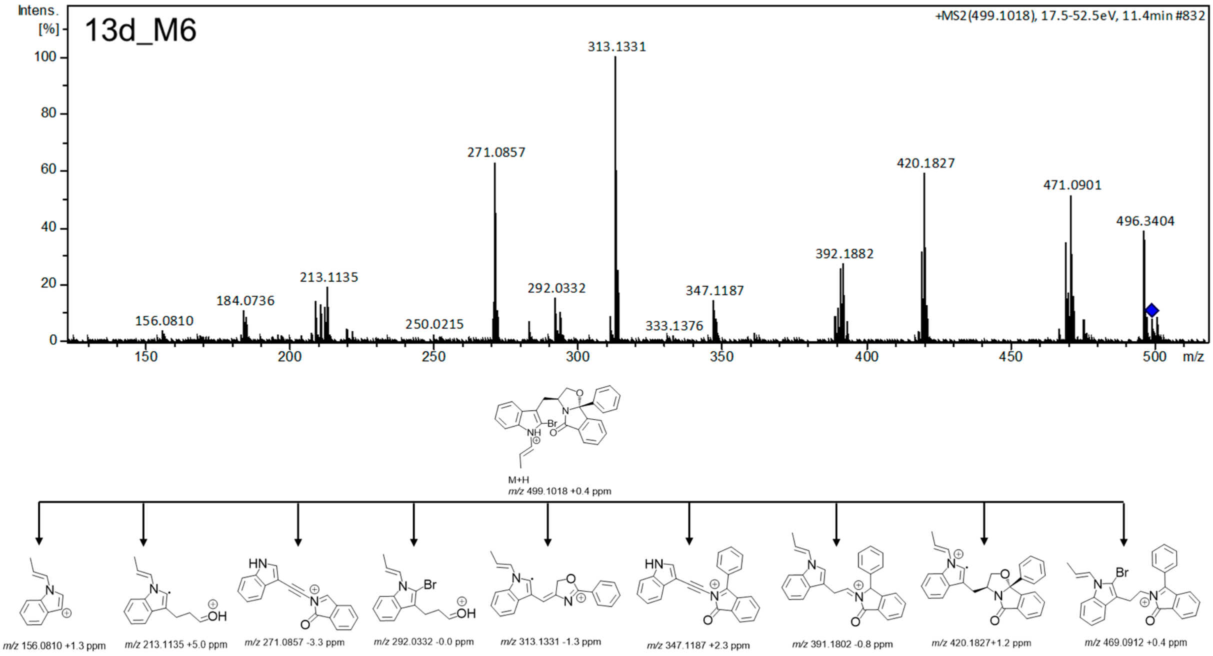


**Figure S47:** LC-ESI(+)-HRMS/MS spectra obtained for **13d_M6** and proposed fragmentation pattern.

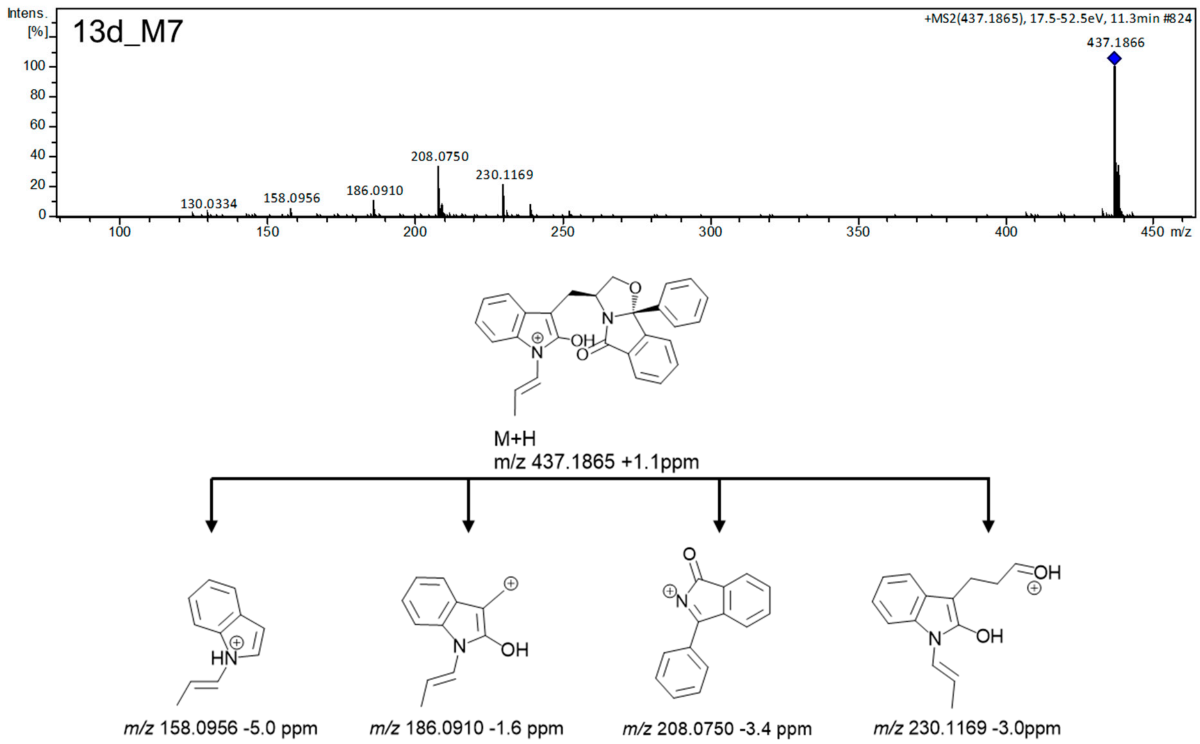


**Figure S48:** LC-ESI(+)-HRMS/MS spectra obtained for **13d_M7** and proposed fragmentation pattern.

# Plots of the relative abundance over HLM incubation time for 13d and 13k and their Phase I metabolites

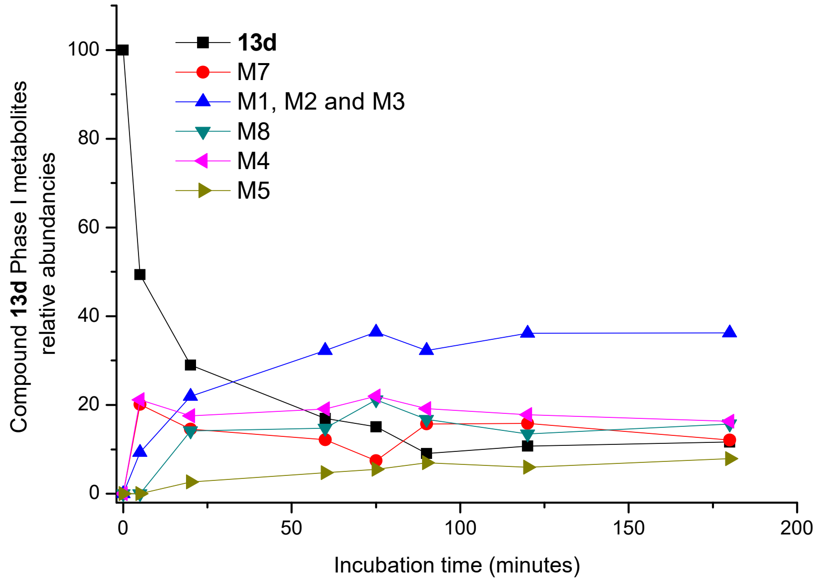


**Figure S49:** Plots of the relative abundances over incubation time for **13d** and its Phase I metabolites.

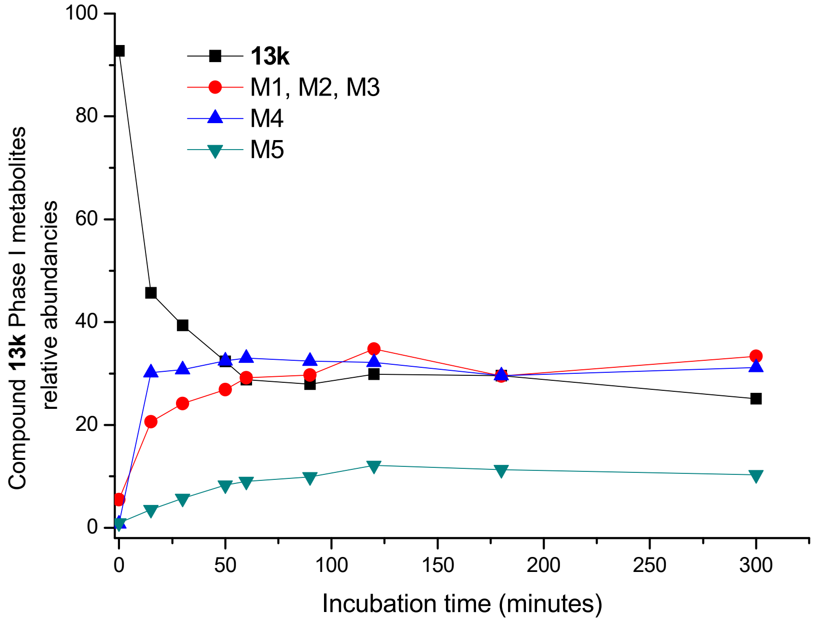


**Figure S50:** Plots of the relative abundances over incubation time for **13k** and its Phase I metabolites.

# LC-HRMS/MS data of 13d and 13k glutathione adducts

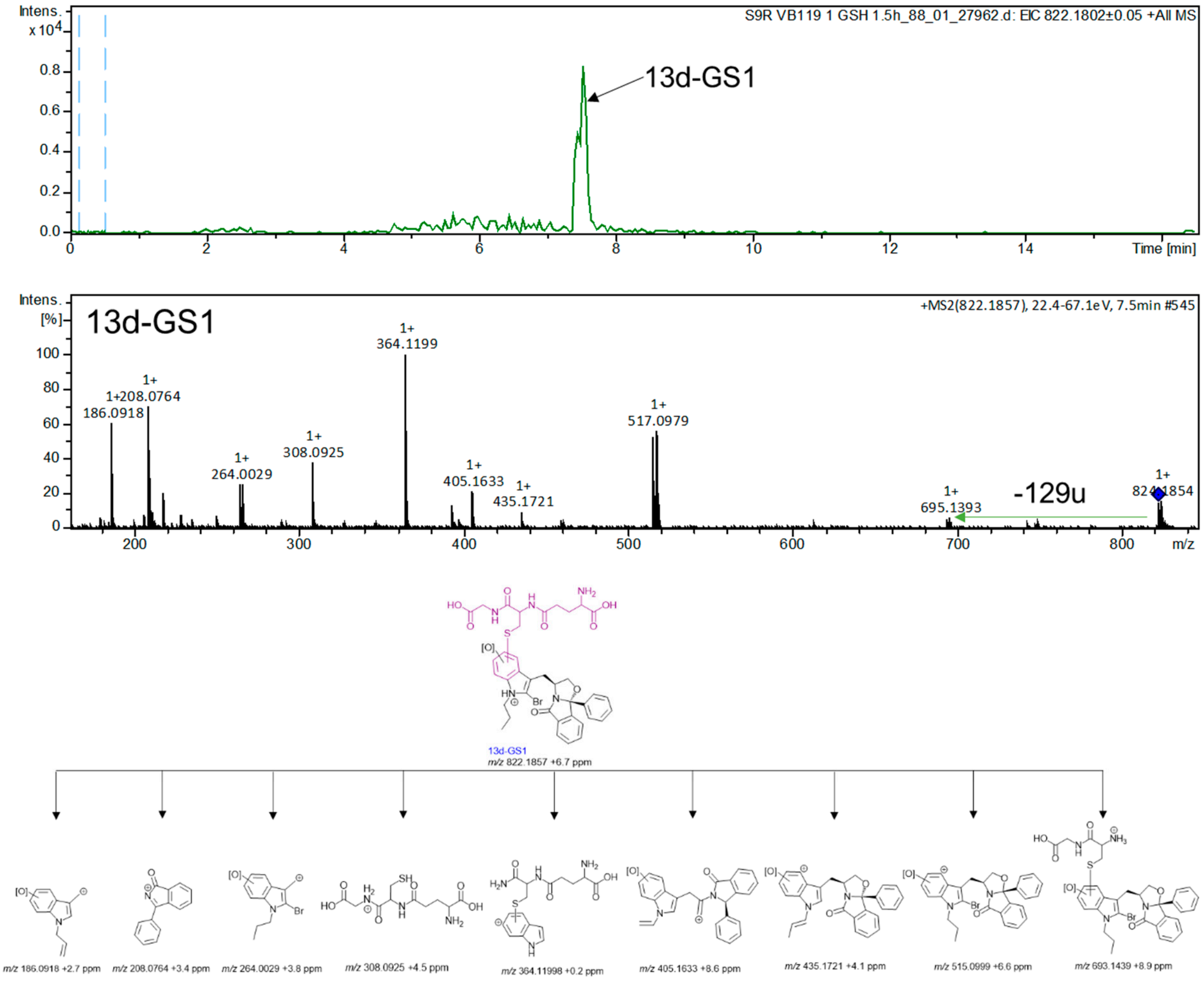


**Figure S51:** Extracted ion HRMS-ESI(+) chromatogram at *m/z* 822.1802. LC-ESI(+)-HRMS/MS spectra obtained for **13d-GS1** and proposed fragmentation pattern.

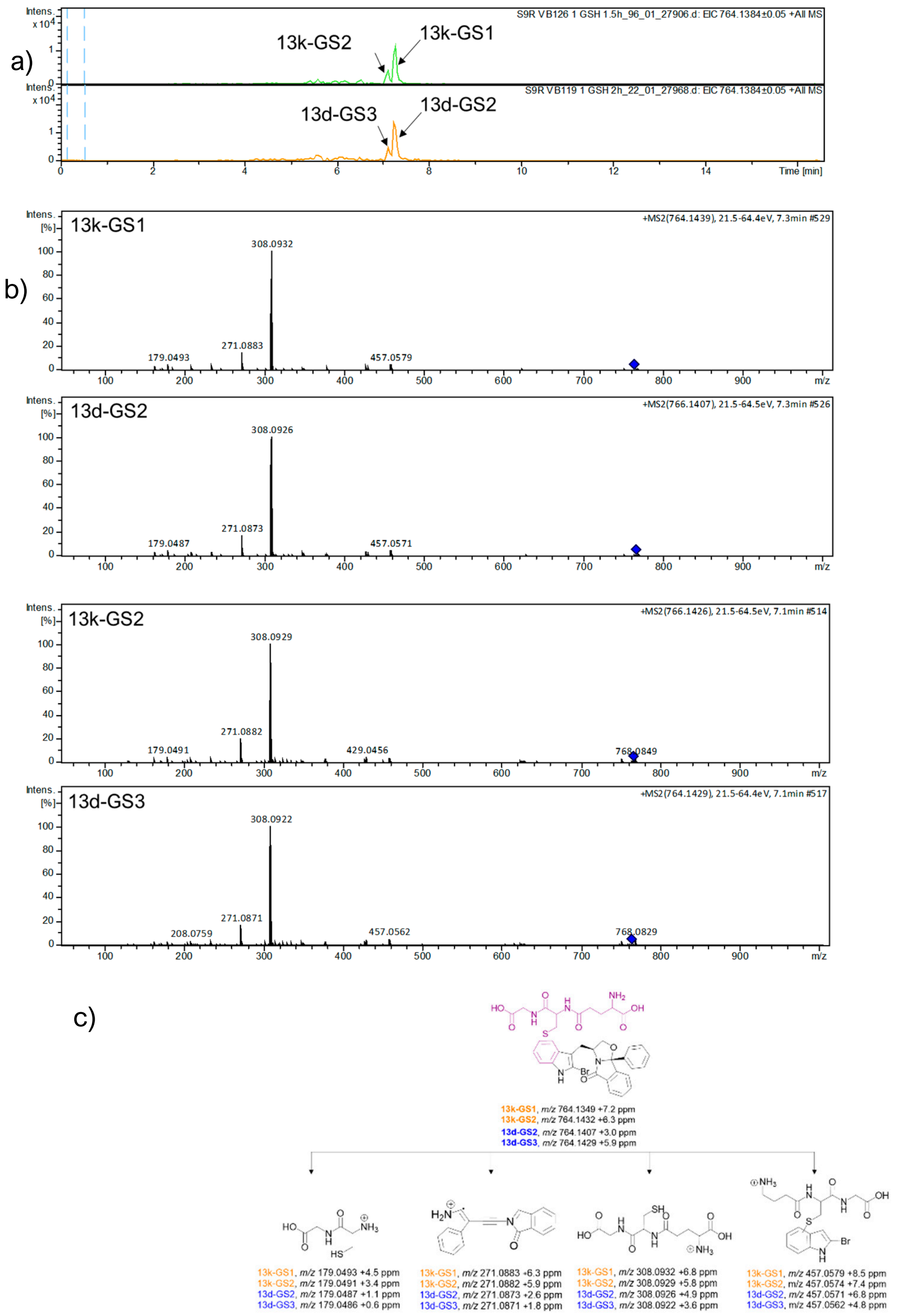

**Figure S52:** a) Extracted ion HRMS-ESI(+) chromatograms at *m/z* 764.1384 of **13d** and **13k** HLM incubations*;* b) LC-ESI(+)-HRMS/MS spectra obtained for **13k-GS1**, **13k-GS2**, **13d-GS2** and **13d-GS3;** and c) proposed fragmentation mechanisms.

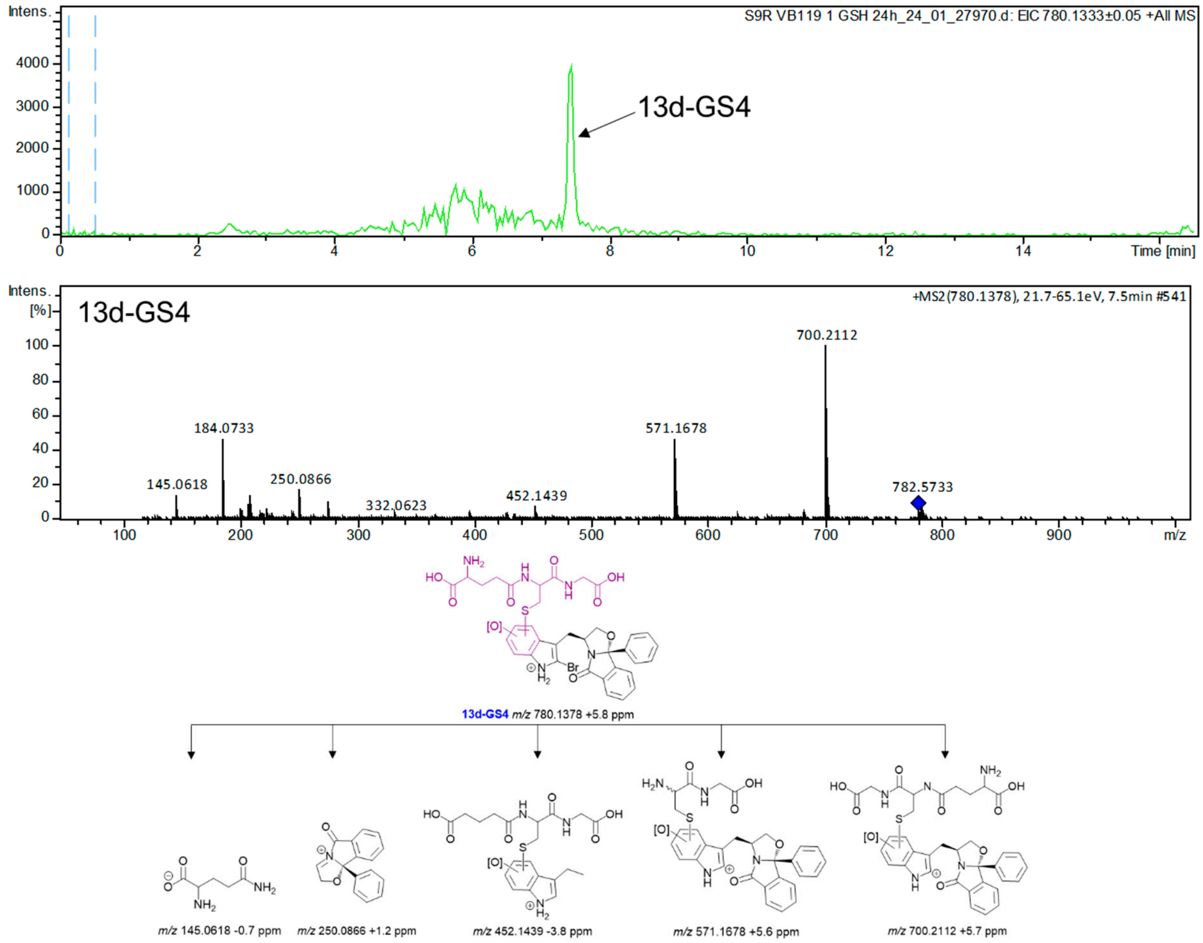


**Figure S53:** Extracted ion HRMS-ESI(+) chromatogram at *m/z* 780.1378. LC-ESI(+)-HRMS/MS spectra obtained for **13d-GS4** and proposed fragmentation mechanisms.

## Thermostability of the wt p53DBD in the presence of studied compounds as monitored by differential scanning fluorimetry (DSF)

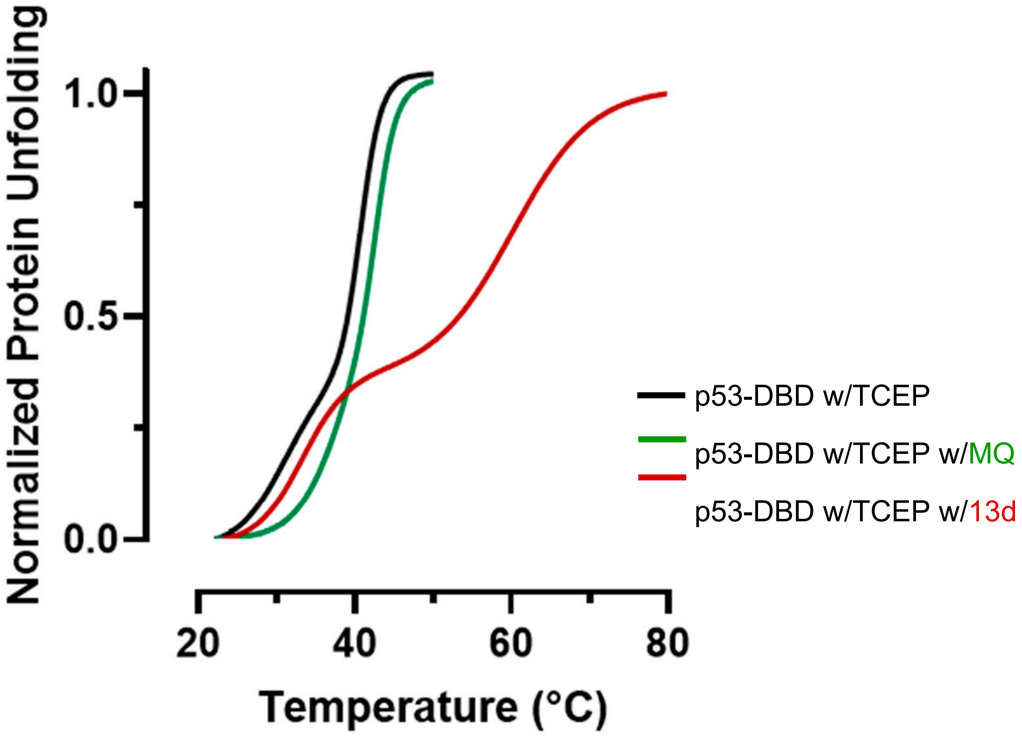


**Figure S54:** Thermal denaturation curves obtained by DSF assay of the wt p53DBD in the absence and presence of compounds. (Black) 1 mM TCEP; (green) 1 mM TCEP and 2 mM of MQ; (red) 1 mM TCEP and 2.5 mM of compound **13d**. The DSF assays were performed with the fluorophore SYPRO Orange. The presence of compound **13d** led to an increase in the wt p53DBD Tm of 10.35 ºC, while in MQ afforded an increase of 2.11 ºC.
